# Efficiently red-emitting dimethoxybenzothiadiazoles for organic doping-free devices enabling stable warm-white electroluminescence

**DOI:** 10.1039/d6tc01121g

**Published:** 2026-07-16

**Authors:** Stepan Kutsiy, Lyudmyla Kanibolotska, Alexander Kanibolotsky, Mykhailo Shchetinin, Khrystyna Ivaniuk, Pavlo Stakhira, Asta Dabuliene, Dmytro Volyniuk, Joseph Cameron, Juozas V. Grazulevicius, Iryna Yaremchuk, Peter J. Skabara

**Affiliations:** a Department of Electronic Engineering, Lviv Polytechnic National University, Sviatoho Yura sq. 1 Lviv 79013 Ukraine stepan.a.kutsii@lpnu.ua; b School of Chemistry, University of Glasgow Glasgow G12 8QQ UK peter.skabara@glasgow.ac.uk; c Institute of Physical-Organic Chemistry and Coal Chemistry 02160 Kyiv Ukraine; d Department of Polymer Chemistry and Technology, Kaunas University of Technology, Barsausko 59, LT-51423 Kaunas Lithuania dmytro.volyniuk@ktu.lt juozas.grazulevicius@ktu.lt

## Abstract

The study provides guidance for developing doping-free white organic light-emitting diodes (OLEDs) with single or double light-emitting layers and offers insights into molecular structure–property relationships for highly efficient dimethoxybenzothiadiazole-based emitters. To achieve white electroluminescence, newly synthesised dimethoxybenzothiadiazole derivatives were used as red fluorescent emitters in combination with emitters exhibiting efficient thermally activated delayed fluorescence (TADF), such as a sky-blue exciplex emitter or a conventional green emitter. The developed compounds exhibit photoluminescence quantum efficiencies of up to 98% in solutions, hole mobilities exceeding 1 × 10^−4^ cm^2^ V^−1^ s^−1^ at electric fields above 1 × 10^6^ V cm^−1^, ionisation energies of *ca.* 5.6 eV, and electron affinities of *ca.* 3.4 eV. These properties enabled us to fabricate doping-free OLEDs with an external quantum efficiency (EQE) of up to 3.34% and red electroluminescence with CIE1931 (*x*, *y*) colour coordinates (0.54, 0.36). The sky-blue exciplex-based TADF emitter tunes the colour coordinates and enhances EQE by up to 8.5%. The green TADF emitter enabled us to achieve stable warm-white electroluminescence in OLEDs with double, fully doping-free light-emitting layers, exhibiting EQE up to 8.23%, and colour coordinates of *x* up to 0.43, and *y* up to 0.38.

## Abstract 2

Novel materials featuring non-covalent interactions are designed for application in doping-free white organic light-emitting diodes.

## Graphical abstract

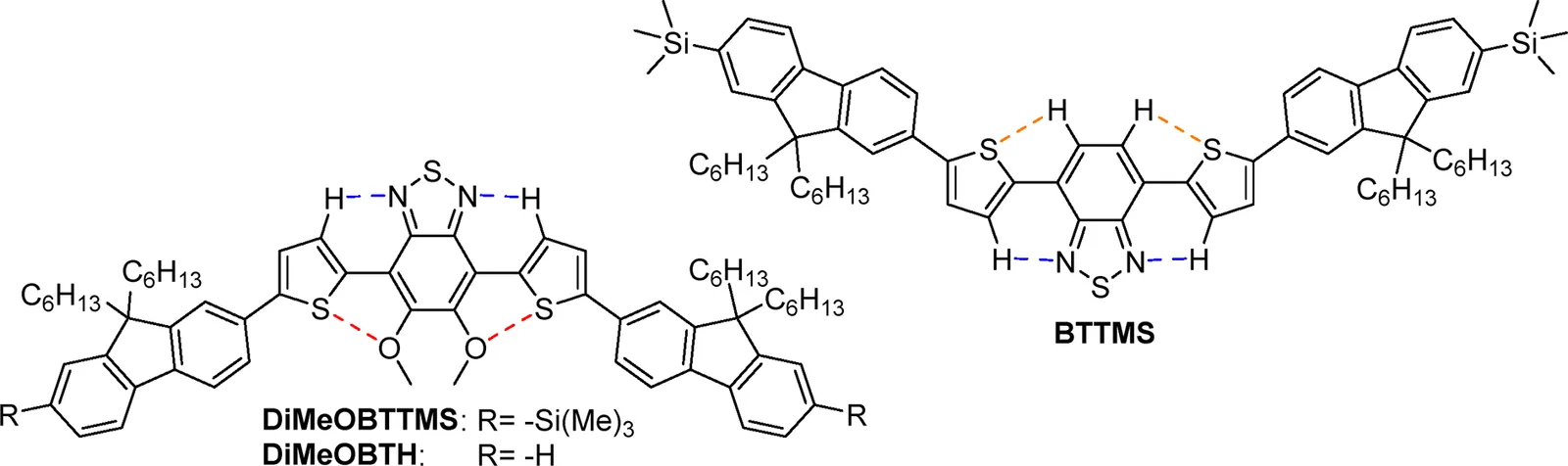

## 1 Introduction

The organic light-emitting diode (OLED) is a fundamental component of modern display technology. In contrast to lead-containing perovskite light-emitting diodes or other hybrid light-emitting systems,^1–6^ low-molecular-weight materials-based OLEDs are characterised by high mechanical flexibility, low fabrication costs, and typically involve environmentally friendly materials.^7,8^ However, OLEDs still have limited applications in the lighting industry.^9–15^ Numerous high-performance white OLEDs (WOLEDs) have been developed utilising multilayer light-emitting structures rooted in a host–guest approach.^16–19^ When the recombination zones of holes and electrons are distributed across several light-emitting layers, white OLEDs frequently demonstrate unstable white electroluminescence colours under varying applied voltages.^20,21^ The host–guest energy transfer process may also differ, further contributing to the instability of white electroluminescence colours across different voltages.^22,23^ Nonetheless, the development of WOLEDs based on a single light-emitting layer without a host is quite limited.^24^ Our objective was to address this issue by employing a device-structural design methodology and molecular-structure engineering approach to identify efficient red-emitting electroactive compounds for WOLEDs with stable white electroluminescence under different electrical power excitations.

The development of multifunctional organic emitters is a key focus in modern OLED research.^18^ Such materials allow for versatile device architectures, improve performance and enable devices of different colours using different architectural solutions.^25–27^

Achieving multifunctionality typically requires careful molecular design, allowing a single compound to operate effectively under different photophysical and process conditions. Advanced host–guest engineering, including the use of exciplex hosts, further extends the functionality of emitters by improving exciton control and enabling mechanisms such as hyperfluorescence.^28,29^ In addition, structures combining fluorescent and thermally activated delayed fluorescence (TADF) emitters provide effective strategies for optimising energy transfer pathways and tuning emission spectra. Together, these technological approaches provide a robust platform for implementing multifunctional materials into high-performance colour-tuneable OLEDs.^25,30,31^

The introduction of functionalities capable of non-covalent interactions around the conjugated backbone of organic semiconductors is a well-known strategy for the rigidification of a conjugated molecule.^32,33^ This rigidification is very important for a high degree of conjugation across the molecule, decreasing HOMO–LUMO gaps and altering their absorption and emission profiles.^34^ The planarisation of the conjugated system also leads to a decrease in charge reorganisation energy, which provides more efficient emission and higher charge mobility.^35,36^

Non-covalent interactions can completely change the behaviour of organic emitters by introducing aggregation-induced emission (AIE),^37^ which has been exploited for facilitating TADF^38^ and tuning the packing mode of D–A–D host materials for high performance blue PhOLEDs.^39^

**Fig. 1:**
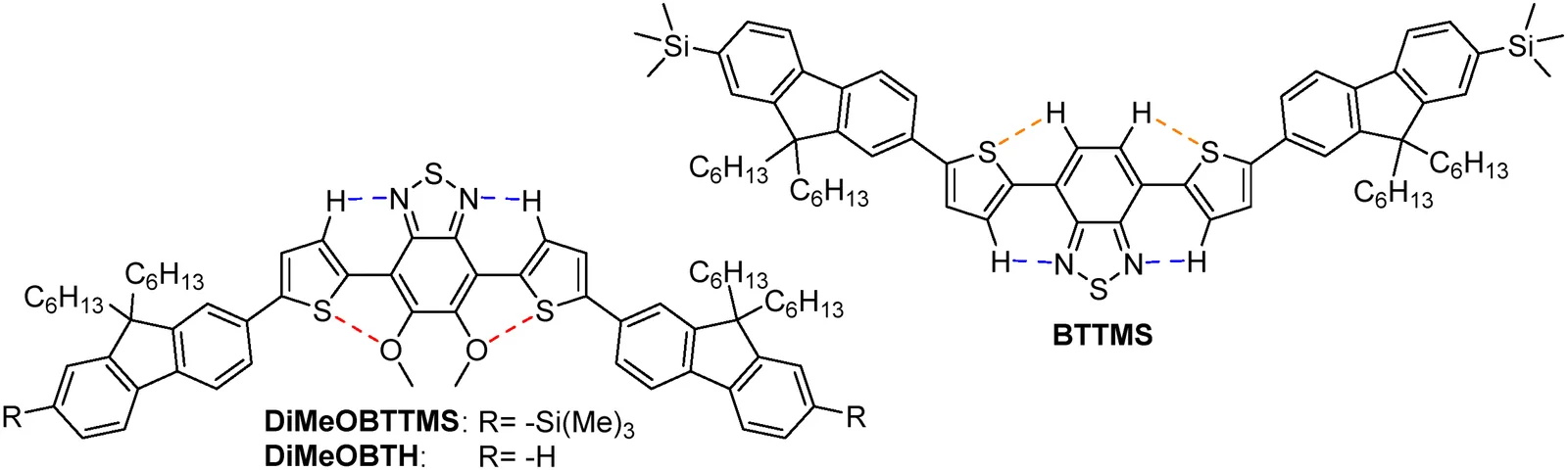
The structure of the target compounds DiMeOBTTMS and DiMeOBTH with the dotted lines showing S⋯O (red), S⋯H (orange) and N⋯H (blue) non-covalent interactions and the structure of the parent compound BTTMS without S⋯O non-covalent interactions reported previously.^41^

In this paper, we present novel dimethoxybenzothiadiazole-based organic emitters, 4,7-bis[5-(9,9-dihexyl-7-(trimethylsilyl)-9*H*-fluoren-2-yl)-2-thienyl]-5,6-dimethoxy-2,1,3-benzothiadiazole (DiMeOBTTMS) and 4,7-bis[5-(9,9-dihexyl-9*H*-fluoren-2-yl)-2-thienyl]-5,6-dimethoxy-2,1,3-benzothiadiazole (DiMeOBTH), featuring S⋯O and N⋯H non-covalent interactions (Fig. 1). These non-covalent interactions are classified as short interatomic contacts, in which the distance between the corresponding atoms is less than the sum of the van der Waals radii for the constituent atoms. They were studied as molecules with red fluorescence, components for hyperfluorescent OLEDs and as emissive materials in warm-WOLEDs based on double-emission-layer device architectures. The commercially available green TADF emitter 9-[4-(4,6-diphenyl-1,3,5-triazine-2-yl)phenyl]-*N*,*N*,*N*′,*N*′-tetraphenyl-9*H*-carbazole-3,6-diamine (DACT-II)^40^ was selected to develop hyperfluorescence light-emitting systems. The combination of the green TADF emitter and the red fluorescent emitter enables full-colour OLEDs with relatively high efficiencies because the TADF component efficiently harvests both singlet and triplet excitons to produce high-intensity green emission, while the red fluorescent emitter provides complementary long-wavelength emission. Together, these emitters generate a broad, balanced emission spectrum spanning the visible region, enabling the device to achieve high-quality full-colour output. Furthermore, the warm-white electroluminescence spectra of the devices remained essentially unchanged across a broad range of applied electrical power.

## 2 Results and discussion

### 2.1 Synthesis

The synthesis of compounds DiMeOBTTMS and DiMeOBTH is presented in Scheme S1 (SI, section “The synthesis of diMethoxyRedBT compounds”). The nitration of veratrole 1^42^ and the subsequent reduction of 1,2-dimethoxy-4,5-dinitrobenzene 2^43^ proceed with excellent yields of 95% and 98%, respectively. The reaction with thionyl chloride in the presence of pyridine as a base was employed to convert 1,2-diamino-4,5-dimethoxybenzene 3 into 5,6-dimethoxybenzo[*c*][1,2,5]thiadiazole 4, and subsequent bromination of 4 with molecular bromine gave 4,7-dibromo-5,6-dimethoxybenzo[*c*][1,2,5]thiadiazole 5, with the yields of 4 and 5 being 55% and 61%, respectively.^44^ Using microwave-initiated Stille coupling of 5 with tributylstannyl-thiophene and subsequent bromination afforded products 6 and 7 in excellent yields of 90–99% and 95–99%, respectively.^45^ The modified Suzuki coupling of the compound 7 with boronic ester, mediated by barium hydroxide, provided the first target compound, DiMeOBTTMS, in a moderate yield of 45–70%. The second target compound, DiMeOBTH, was obtained by protodesilylation of DiMeOBTTMS with a very good yield of 85–90%.

### 2.2 Electrochemical properties and thermal properties

**Fig. 2:**
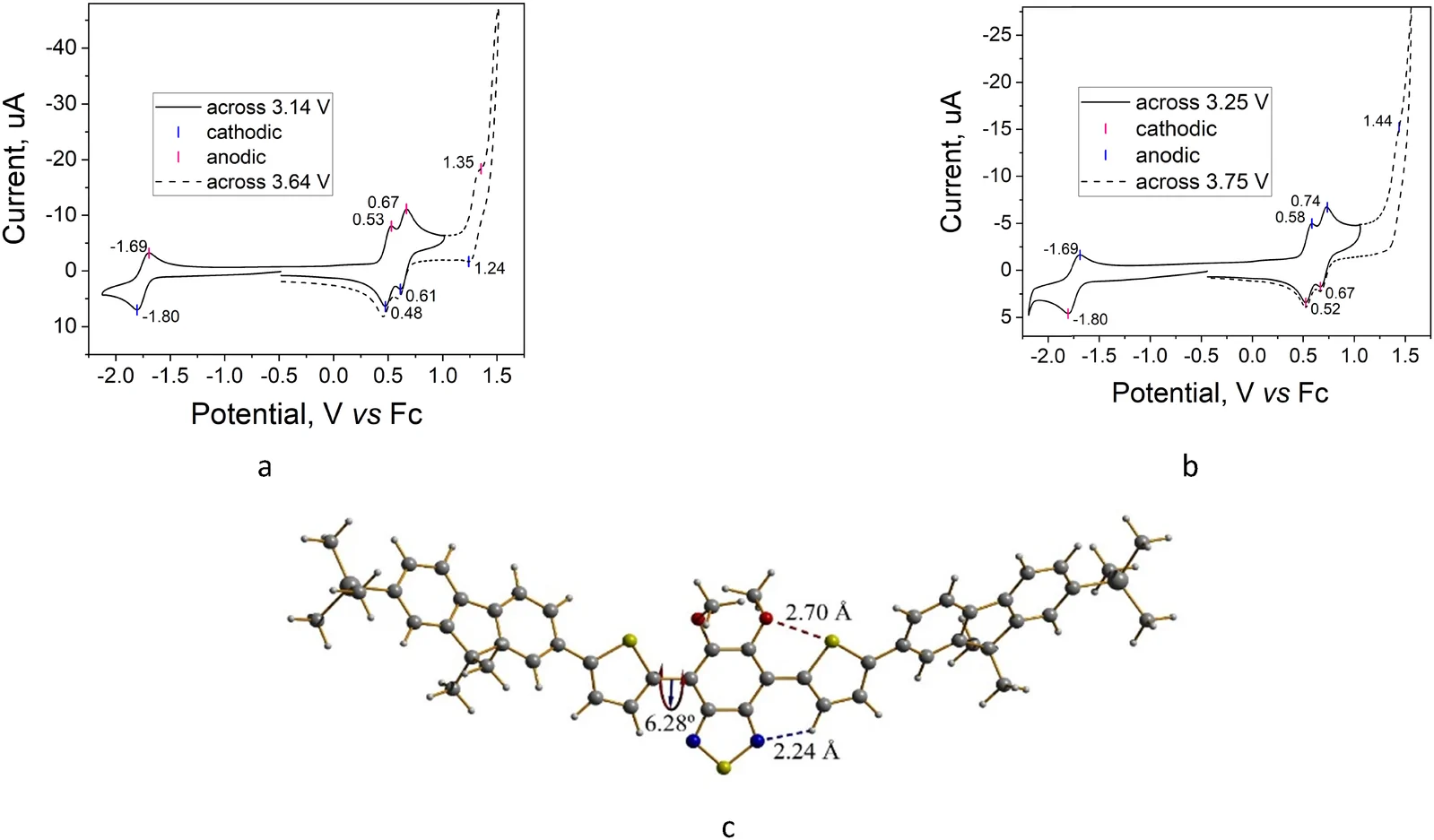
The cyclic voltammograms of (a) DiMeOBTTMS and (b) DiMeOBTH in 0.3 mM solution in CH_2_Cl_2_ with 0.1 M TBAPF_6_ as supporting electrolyte, glassy carbon disc as a working electrode, platinum wire as a counter electrode and silver wire as a quasi-reference electrode. The potential is referenced to the Fc/Fc^+^ redox couple. (c) The structure of the tetramethyl analogue of DiMeOBTTMS obtained by calculations. The C_3-Th_–C_2-Th_–C_4-BT_–C_3a-BT_ torsion angle is shown. The close contacts are shown by red (S⋯O) and blue (N⋯H) dashed lines.

Cyclic voltammetry showed one symmetric quasi-reversible/reversible reduction wave at *E*_1/2_ = −1.75 V for both dimethoxy-BT compounds, indicating that the TMS group in DiMeOBTTMS had no effect on the reduction potential and hence a negligible inductive effect (Fig. 2a, b and Table 1). The oxidation of the dimethoxy-BT compounds featured two reversible oxidation waves, corresponding to the removal of the electrons from the HOMO of the neutral molecule and the SOMO of the corresponding cation radical, respectively. In contrast to the reduction, the oxidation was affected by the TMS substituents and the *E*_1/2_ of these waves shifted to higher potentials by 50–60 mV for DiMeOBTH, compared to DiMeOBTTMS, which is consistent with an electron-donating effect of the TMS groups. The dimethoxy-BT compounds gave a third quasi-reversible and irreversible oxidation wave for the TMS-terminated and H-terminated systems, respectively, with the anodic waves revealed as inflection points at 1.35 V for DiMeOBTTMS and 1.44 V for DiMeOBTH on the overoxidation curve. The higher value of the anodic oxidation inflection point for DiMeOBTH is also in line with the donor effect of the terminal TMS groups. The HOMO–LUMO energy gap values estimated from the results of CV measurements, 2.25 eV for DiMeOBTTMS and 2.30 eV for DiMeOBTH, are significantly lower than the values obtained from DFT calculations, 2.72 and 2.71 eV. This can be explained by a non-specific solvation effect, which is not accounted for the DFT calculations. The hydrogen bonding between polarised C–H bonds of CH_2_Cl_2_ solvent molecules and the HOMO between the long pairs of the BT-nitrogen atoms as well as the oxygen atoms of methoxy groups can significantly stabilise the LUMO. Similar hydrogen bonding can lead to the destabilisation of the HOMO, though to a lesser extent. Both the destabilisation of the HOMO and in a higher extent stabilisation of the LUMO would lead to a significantly narrower HOMO–LUMO gap observed by CV measurements compared to that calculated by DFT.

**Table 1:** Results of electrochemistry and thermal analysis

Compound	*E*_1/2_ [V *vs*. Fc]/DE_p_, mV		Energy levels^a^, eV			HOMO–LUMO gap, eV		*T*_de-5%_, °C	*T*_g_, °C
	Oxidation	Reduction	IE	HOMO	LUMO	EC	Optical^b^		
DiMeOBTTMS	0.50/55, 0.64/57, 1.30^c^/114	−1.75/109	5.57	−5.30 (−5.13)^d^	−3.05 (−2.41)	2.25 (2.72)	2.18	395	75
DiMeOBTH	0.55/60, 0.70/66, 1.44^c^	−1.75/116	5.64	−5.35 (−5.12)	−3.05 (−2.41)	2.30 (2.71)	2.19	389	58
BTTMS	0.44/50, 0.62/44	−1.67/65, −2.20	—	−5.24 (−5.20)	−3.13 (−2.65)	2.12 (2.55)	—	—	—

a Orbital energy for HOMO/LUMO were calculated from *E*_1/2_ for the first oxidation/reduction wave using the formula HOMO/LUMO = –*e*·*E*^ox/red^_1/2_ − 4.8 eV,^46,47^ where *e* is an elementary charge. If the value of −5.1 eV^46^ is used for the HOMO of Fc then the HOMO levels of −5.60 and −5.65 eV are in good agreement with the IE values of 5.57 and 5.64 eV for DiMeOBTTMS and DiMeOBTH, respectively.

b Optical HOMO–LUMO gaps were calculated from the onset of the absorption, using the formula *E* = *h* (in [eV s])·*c* (in [nm s^−1^])/*λ*_onset_ (in [nm]) = 1239.84/*λ*_onset_.

c The inflection point on the over-potential part of the anodic scan is quoted.

d The brackets represent the energy value obtained by DFT calculations in the gas phase at the PBE0/6-31G(d,p) level of theory.

**Fig. 3:**
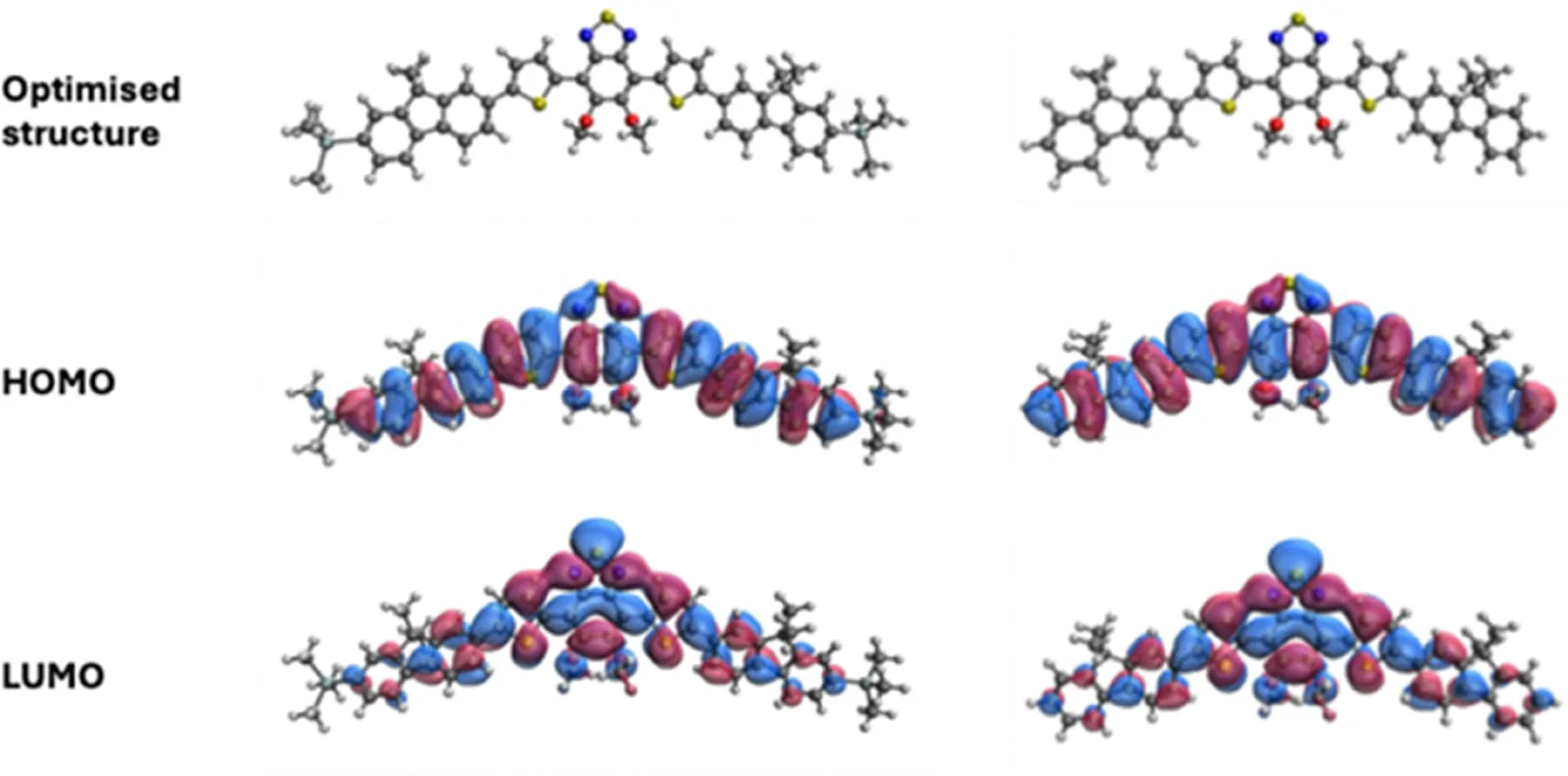
Structure and frontier orbitals (HOMO and LUMO) of 4,7-bis[5-(9,9-Dihexyl-7-(trimethylsilyl)-9*H*-fluoren-2-yl)-2-thienyl]-5,6-dimethoxy-2,1,3-benzothiadiazole (DiMeOBTTMS) (left) and 4,7-bis[5-(9,9-Dihexyl-9*H*-fluoren-2-yl)-2-thienyl]-5,6-dimethoxy-2,1,3-benzothiadiazole (DiMeOBTH) (right). Optimisations carried out in gas phase at the PBE0/6-31G(d,p) level of theory.

It is interesting to compare the TMS-terminated molecules featuring non-covalent interactions between the BT core and thiophenes (DiMeOBTTMS) with the corresponding system without substitution in the BT core (BTTMS), which has been reported previously (Fig. 1).^41^ The reduction potential of the first oxidation of BTTMS (*E*_1/2_ = −1.67 V) is shifted towards higher potentials compared to DiMeOBTTMS, and a second irreversible wave was observed at −2.20 V. No second reduction waves have been observed for the corresponding dimethoxy compound DiMeOBTTMS. The higher reduction potential of the first reduction and the presence of the second irreversible wave in the reduction CV of BTTMS is consistent with the donating effect of the methoxy groups, which makes it harder to reduce the BT acceptor core of the DiMeOBTTMS molecule. The BTTMS system also reveals two reversible oxidation waves, with the oxidation potentials being similar (within experimental error) to those observed for DiMeOBTTMS. According to DFT calculations (Fig. 3 and Tables 2, 3) the structure of the tetramethyl analogue of DiMeOBTTMS exhibits close S⋯O and N⋯H contacts which lead to a low value for the corresponding torsion angles between BT and thiophene units (Fig. 2c). However, there is little difference in spatial overlap of excitations determined by time-dependent density functional theory (TDDFT) (Table 3), whether methoxy groups are present or not. The calculated Δ*E*_ST_ turned out to be quite high (Table 3) which confirmed poor spatial separation (significant overlap) between HOMO and LUMO (Fig. 3).

**Table 2:** Notable bond lengths and dihedral angles from structures calculated using DFT. PBE0/6-31G(d,p), gas phase

	DiMeOBTTMS	DiMeOBTH	BTTMS	BTH
S–O, Å	2.70	2.71	—	—
S–O, Å	2.70	2.71	—	—
C–H–N, Å	2.23	2.24	2.32	2.31
C–H–N, Å	2.24	2.24	2.32	2.31
S–C–C–C dihedral *θ*_1_, °	7.6	8.3	−6.1	−4.8
S–C–C–C dihedral *θ*_2_, °	7.6	8.3	−6.1	−4.8

**Table 3:** Summary of calculated energies from TDDFT (Tamm–Dancoff approximation). PBE0/6-31G(d,p), gas phase

	HOMO, eV	LUMO, eV	HOMO–LUMO, eV	S_1_, eV	T_1_, eV	Δ*E*_ST_, eV	Orbital overlap S_1_	Orbital overlap T_1_
DiMeOBTTMS	−5.13	−2.41	2.72	2.28 (543 nm)	1.57 (791 nm)	0.71	0.67	0.57
DiMeOBTH	−5.12	−2.41	2.71	2.28 (543 nm)	1.56 (793 nm)	0.72	0.67	0.57

This can be explained by the fact that the thienyl-benzothiadiazole group already forms a conformational lock due to the presence of non-traditional hydrogen bonds.^48^ Therefore, the influence of the methoxy groups is maintaining a conformational lock through the S·O contact (and N·H contact), while inducing increased HOMO and LUMO energies relative to vacuum. As the methoxy group is situated on the acceptor part, this effect is more prominent on the LUMO energy, resulting in an increase in the HOMO–LUMO energy gap. The effect of the non-covalent interactions described here is quite different from that of the S⋯N non-covalent interactions between the non-coupled thiazole acceptor and tetrathiafulvalene (TTF) donor units observed by Taylor *et al.*, in which the donation from the nitrogen lone pair of the thiazole unit to the sulfur atoms of the TTF unit led to the destabilisation of the molecule's HOMO level.^49^ The calculated electrochemical HOMO–LUMO gaps of DiMeOBTTMS and DiMeOBTH are in good agreement with their optical gaps, estimated from the absorption onsets, and slightly higher than the latter by around ∼0.1 eV due to the exciton binding energy.^50^

The thermal stability and the thermal transition temperatures of compounds DiMeOBTTMS and DiMeOBTH were studied by thermogravimetric analysis (TGA) and differential scanning calorimetry (DSC). The obtained data are presented in Table 1. Both DiMeOBTTMS and DiMeOBTH turned out to be thermally stable, with decomposition temperatures representing five percent weight loss being 395 °C and 389 °C, respectively (Fig. S5a).

Both compounds DiMeOBTTMS and DiMeOBTH were isolated as amorphous substances after their synthesis. No peaks corresponding to crystallisation or melting appeared during the cooling and heating cycles between −40 °C and 330 °C. Only glass transition signals were observed in the heating and cooling scans (Fig. S5b and c). The glass-transition temperatures (*T*_g_) obtained from repeated heating scans were determined to be 75 °C and 58 °C, respectively, for compounds DiMeOBTTMS and DiMeOBTH (Table 1). It is evident that the trimethylsilyl groups do not significantly affect the thermal stability of the synthesised compounds, but they increase the glass transition temperature by more than 15 °C (comparing DiMeOBTTMS and DiMeOBTH), as their steric hindrance restricts segmental mobility and hinders rotation around chemical bonds.^51^ The effect of substituents on the value of the glass transition temperature for monodispersed macromolecular systems depends on how the substitution changes the steric hindrance of the molecule towards the thermal motions, compared to the total change of the molecular free volume. Substitutions at terminal positions normally increase *T*_g_ as they create more steric hindrance. This can be seen within a series of star-shaped oligofluorene-truxene systems while increasing the length of the arms in both hexyl,^52^ butyl and octyl series.^53^ However, comparison of the thermal properties of the olyfluorene-truxene systems with the same arm lengths but with different alkyl substituents reveals the systematic decrease in *T*_g_ in the series of butyl, hexyl, octyl oligomers due to the increase in the molecular free volume.^53^ The effect of the molecular free volume on *T*_g_ can be seen in the lower *T*_g_ of a DPP-Star oligofluorene compared to its linear analogue (DPP-Linear-nc).^54^ When implementing the change in both terminal substituents (*e.g.*, increasing the length of oligofluorene) and side chains (modification of alkyl groups in the 9,9′-positions of the fluorenylidene repeat unit) in the linear oligofluorene systems, the effect of substitution on *T*_g_ can be more complicated.^55^ In the case of DiMeOBTTMS the TMS groups don’t greatly affect free volume of the molecule compared to that of DiMeOBTH but increase the steric hindrance towards thermal motion, which leads to an increase in *T*_g_.

### 2.3 Photophysical properties

**Fig. 4:**
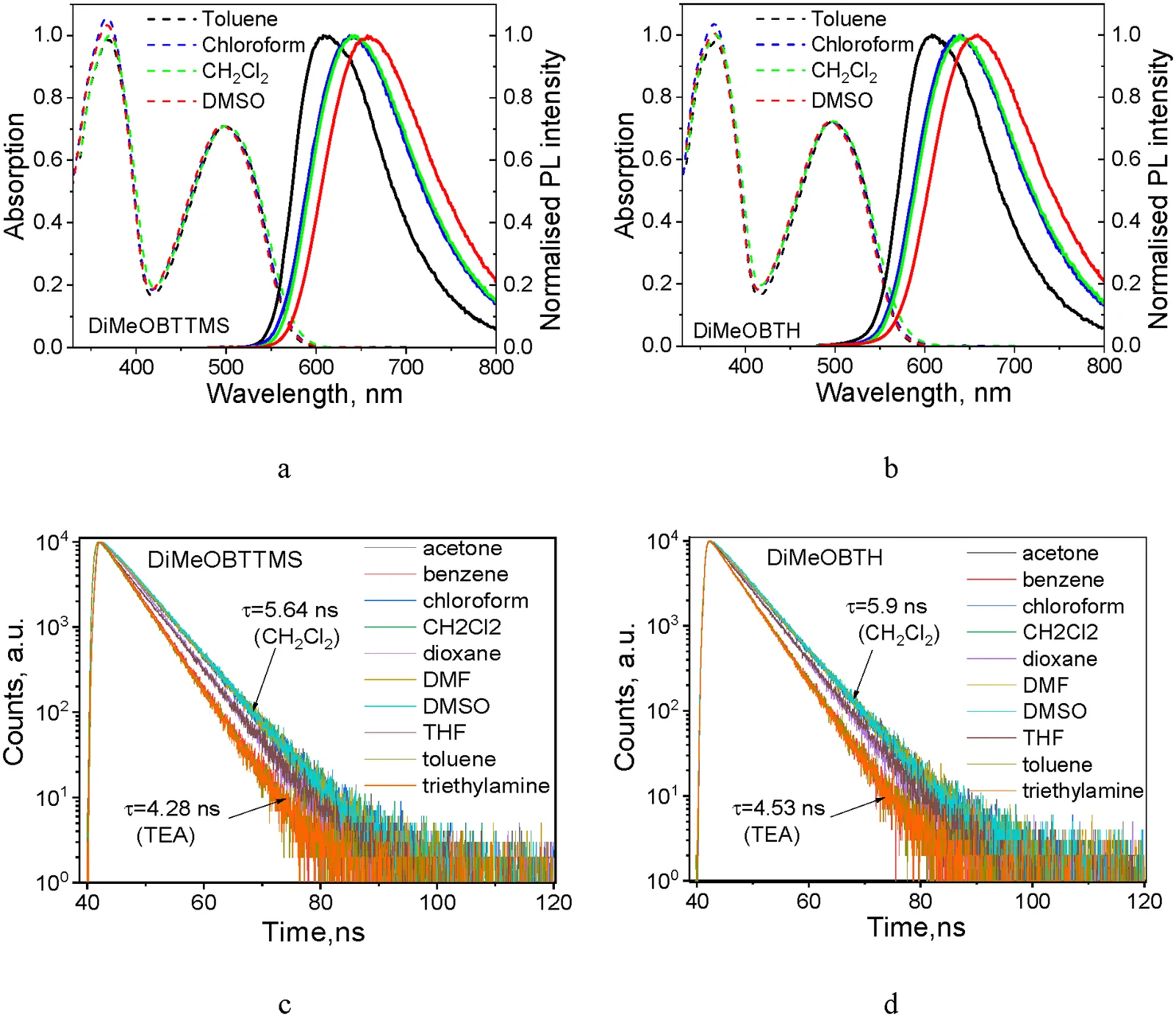
Absorption and PL spectra (a) and (b) and PL decay curves (c) and (d) of dilute solutions of DiMeOBTTMS and DiMeOBTH in various solvents. Absorption spectra are represented by dashed curves, while PL spectra are depicted by solid curves. Absorption and PL spectra of other solutions are provided in Fig. S6.

The absorption spectra of compounds DiMeOBTTMS and DiMeOBTH in various solvents were measured (Fig. 4a, b and Fig. S6a,b). The study was conducted in solvents of different polarity, including acetone, benzene, chloroform, dichloromethane (CH_2_Cl_2_), dioxane, dimethylformamide (DMF), dimethyl sulfoxide (DMSO), tetrahydrofuran (THF), toluene, and triethylamine (TEA). Absorption spectra with two bands were obtained, showing negligible variations in position and relative intensities. The absorption spectra of both dimethoxy-BT compounds revealed intense bands of delocalised-delocalised π–π* (362–368 nm) and delocalised-localised intramolecular charge transfer (ICT) (495–497 nm) transitions.^56^ This is blue-shifted compared to the analogous compound without methoxy groups (BTTMS)^41^ and this trend is observed in the TDDFT results (Table S1), while the similar nature of the ICT band is consistent with the fact the spatial overlap is measured to be similar in both compounds. Overall, the influence of the solvents on the shape of absorption spectra of DiMeOBTTMS and DiMeOBTH is deemed to be minimal. The absorption coefficient (*ε*) values for both compounds are the same, taking into account the errors of the experiment (Table 4).

**Table 4:** Photophysical parameters of toluene and CH_2_Cl_2_ solutions and 1wt% in Zeonex, 1wt% in CBP, and neat solid films of DiMeOBTTMS and DiMeOBTH

Compound	*λ*_abs_,^a^ nm	*ε* 10^4^,^a^ M^−1^ cm^−1^	*λ*_PL_,^a^ nm	PLQY,^a^ %	*τ*_PL_,^a^ ns	*λ*_PL_,^b^ nm	PLQY,^b^ %	*τ*_PL_,^b^ ns
	Toluene/CH_2_Cl_2_					1 wt% in Zeonex/1 wt% in CBP/neat film		
DiMeOBTTMS	498/494	4.08 ± 0.12/4.07 ± 0.17	605/640	93.74/92.34	4.37/5.64	596/621/638	65/46/36	4.6/2.33, 5.09/0.92, 2.05
DiMeOBTH	496/496	4.29 ± 0.84/4.22 ± 0.07	605/633	98.44/94.18	4.59/5.9	594/620/635	71/48/15.1	4.23/2.7, 5.37/1.17, 2.07

a The values are separated by the slashes for toluene and CH_2_Cl_2_ solutions of DiMeOBTTMS and DiMeOBTH, respectively.

b The values are separated by the slashes for 1 wt% in Zeonex, 1 wt% in CBP, and neat solid films of DiMeOBTTMS and DiMeOBTH, respectively.

In contrast to the absorption spectra, the shapes and positions of the photoluminescence (PL) spectra of various solutions of DiMeOBTTMS and DiMeOBTH were sensitive to the solvent medium (Fig. 4a, b and Fig. S6c, d). For example, the PL maxima of ten solutions of DiMeOBTTMS ranged from 584 nm (for TEA) to 659 nm (for DMSO) (Table S1). The full width at half maximum (FWHM) values in these solutions ranged from 81 nm for low-polarity TEA (dielectric constant (*ε*) of *ca.* 2.42) to 138 nm for high-polarity DMSO (*ε* of *ca.* 46.7). This observation suggests that the emission band originated from ICT transitions because polar solvents stabilise excited states of DiMeOBTTMS and DiMeOBTH more effectively than their ground states. Such positive solvatochromism is typically observed for donor–acceptor-type organic dyes.^57^ The fairly broad PL spectra of DiMeOBTTMS in different media indicate it is suitable for white electroluminescence applications. A similar wavelength range of PL maxima (from 583 nm for TEA to 659 nm for DMSO) and variation of FWHM values (between 82 nm for TEA and 137 nm for DMSO) were observed for ten solutions of DiMeOBTH (Table S1). When the polarity of the surrounding solvents increases, similar PL shifting, broadening of the PL spectra and increasing of FWHM values were observed for both compounds DiMeOBTTMS and DiMeOBTH, apparently because of their similar dipole moments. To support this assumption, the Lippert–Mataga dependences of the Stokes shifts on the orientational polarisability of the solvent were analysed to evaluate their dipole moments in the excited and ground states (Fig. S7). The slopes of the Lippert–Mataga plots correspond directly to the difference in the molecule's dipole moments between the excited and ground states. The similar Lippert–Mataga slope values for DiMeOBTTMS and DiMeOBTH were 4552 cm^−1^ and 4765 cm^−1^, respectively. Such values are typically observed for compounds with low dipole moments.^58–61^ On the other hand, high charge carrier mobilities can be expected for compounds with low dipole moments.^62,63^ Indeed, hole mobility values of DiMeOBTTMS and DiMeOBTH exceeded 1 × 10^−4^ cm^2^ V^−1^ s^−1^ at high electric fields, as demonstrated in the next section.

**Fig. 5:**
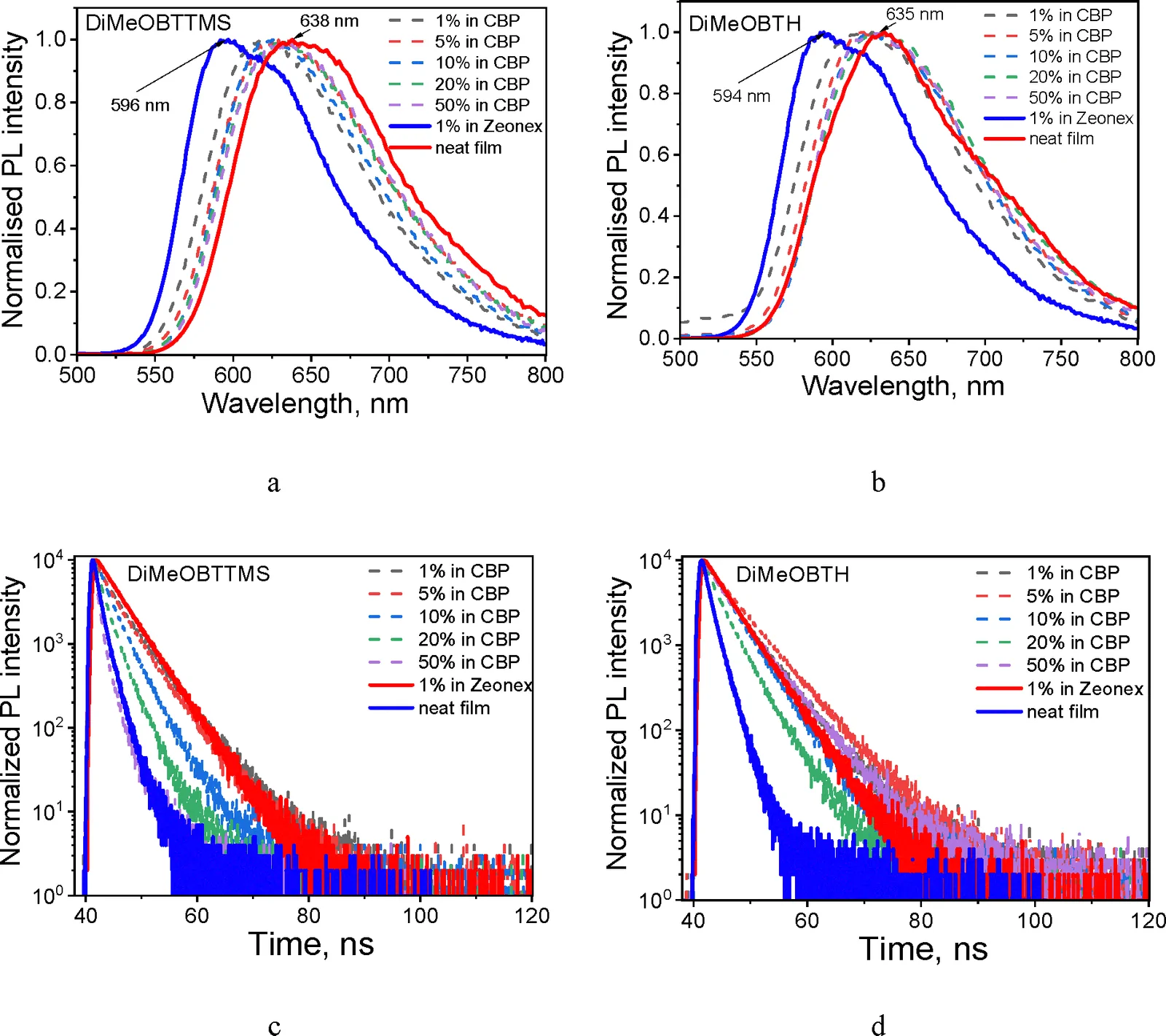
PL spectra (a) and (b) and PL decay curves (c) and (d) of host-based (Zeonex or CBP) and neat films of compounds DiMeOBTTMS and DiMeOBTH.

The photoluminescence decay curves of DiMeOBTTMS and DiMeOBTH solutions were accurately fitted by a monoexponentially decay function with low error (1 > *χ*^2^ > 1.3) as described in the SI, section “Spectroscopic Measurements and Analyses”. This fitting shows fluorescence lifetimes in the range of 4.28–5.64 ns and 4.53–5.9 ns for solutions of DiMeOBTTMS and DiMeOBTH, respectively (Fig. 4c, d and Table S2). The exponential decay of fluorescence intensity corresponds to the typical radiative relaxation process of singlet excited states. Long-lived emission was not detected for DiMeOBTTMS and DiMeOBTH, neither in oxygenated nor in deoxygenated solutions (Fig. S8). The fluorescence lifetimes of DiMeOBTTMS and DiMeOBTH are weakly sensitive to solvent polarity. Nonetheless, the photoluminescent quantum yields (PLQYs) of solutions of DiMeOBTTMS and DiMeOBTH exceeded 90% (Table 4). Aggregation-caused quenching (ACQ) was observed for DiMeOBTTMS and DiMeOBTH, resulting in relatively low PLQY values of 36% and 15.1% in their neat films, respectively (Table 4). ACQ is likely to be the consequence of dimer formation in the solid phase due to strong dipole–dipole interactions, which would lead to π–π interactions, quenching the photoluminescence. Using host materials facilitated in part to overcome the ACQ of DiMeOBTTMS and DiMeOBTH. For example, PLQY values of 65% and 71% were obtained for DiMeOBTTMS and DiMeOBTH, respectively, when dispersed (1 wt%) in an inert, low-polarity polymeric matrix, Zeonex (*ε* of *ca.* 2.3).^64,65^ The maximum PLQY values of 46% and 48% were obtained for DiMeOBTTMS (1 wt%) and DiMeOBTH (1 wt%) dispersed in a host 4,4′-bis(9-carbazolyl)-biphenyl (CBP), respectively (Table 4 and Table S2). The increasing concentration of guests in CBP-based films led to a slight decrease in PLQY values, as observed with ACQ (Table S3). The PL maxima of films of DiMeOBTTMS and DiMeOBTH were in the ranges of 596–638 nm and 594–635 nm, respectively (Fig. 5a and b). Thus, the polarity of the surrounding hosts Zeonex and CBP or neat films affects the emission properties of DiMeOBTTMS and DiMeOBTH. Because of the low dielectric constant of Zeonex, the PL spectra of Zeonex-based films were the most blue-shifted. The PL spectra of the neat films were the most red-shifted, apparently due to the higher *ε* values of DiMeOBTTMS and DiMeOBTH in comparison to that of Zeonex and CBP. This observation is similar to solvatochromic effects. Similarly, varying the concentrations of DiMeOBTTMS and DiMeOBTH caused shifts in the PL spectra and changes in the FWHMs of CBP-based films, because the different concentrations of DiMeOBTTMS and DiMeOBTH induce different *ε* values in CBP-based films (Fig. 5a, b and Fig. S8). Thus, the emission colours of DiMeOBTTMS and DiMeOBTH can be tuned as a function of the concentrations of the composite film.

PL spectra and PL decay curves of thin Zeonex and CBP-containing films of the compounds DiMeOBTTMS and DiMeOBTH were recorded under vacuum and air conditions. PL intensities of Zeonex and CBP films were largely independent of the presence of oxygen, indicating that triplets were not emissively relaxed (Fig. S8 and Table S3). As a result, PL decay curves were obtained in the nanosecond range (Fig. 5c and d). The analysis of fluorescence parameters showed that the average fluorescence lifetime (*τ*_PL_) for DiMeOBTTMS (1 wt%) dispersed in Zeonex was 4.16 ns, whereas for DiMeOBTH (1 wt%) dispersed in Zeonex, it was 4.23 ns (Table 4 and Table S3). Those values are comparable with those of low-polarity TEA solutions of DiMeOBTTMS and DiMeOBTH (Table S1). Due to ACQ, smaller τ_PL_ values were obtained for CBP-based and neat films compared to Zeonex-based films. Moreover, PL decay curves were well-fitted (1 > *χ*^2^ > 1.3) when the double exponential decay formula was used. Nevertheless, to harvest triplets of DiMeOBTTMS and DiMeOBTH under electrical excitations, these red emitters should be used in combination with triplet-harvesting emitters, such as TADF compounds,^66,67^ as the singlet–triplet gap for DiMeOBTTMS and DiMeOBTH is too large for reverse intersystem crossing of the triplet states, as shown from TDDFT results (Table 3).

### 2.4 Energy levels and charge-transporting properties of vacuum-deposited films

**Fig. 6:**
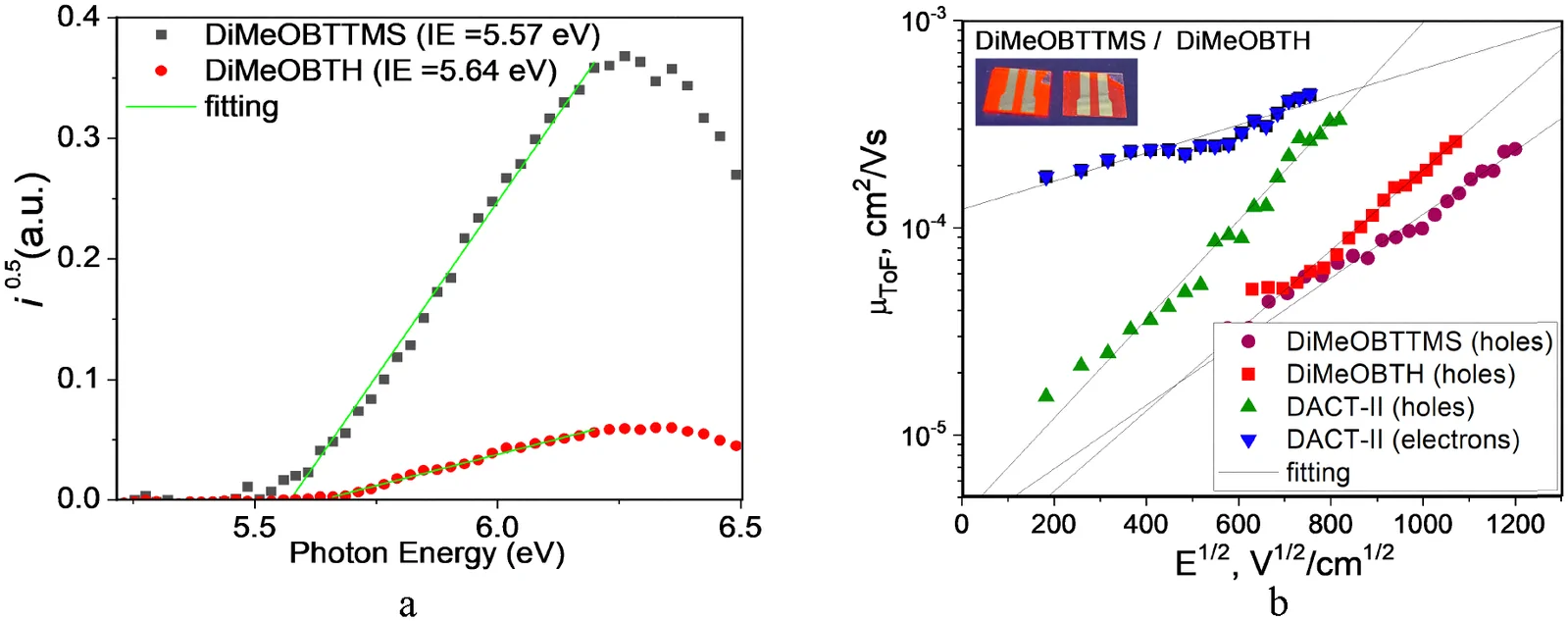
Photoelectron emission spectra (a) and electric field dependences of hole mobilities (b) of vacuum-deposited layers of DiMeOBTTMS and DiMeOBTH. The inset shows TOF samples with the structure ITO/film/Al under UV excitation.

Electrons emitted from films of DiMeOBTTMS and DiMeOBTH under ultraviolet excitation with photon energy above *ca.* 5.6 eV were detected in air at the counter electrode, resulting in current (*i*) in the electric circuit. This detection allowed us to record photoelectron emission spectra and obtain similar ionisation energies (IE) for DiMeOBTTMS and DiMeOBTH at 5.57 eV and 5.64 eV, respectively (Fig. 6 and Table S4). These IE values for DiMeOBTTMS and DiMeOBTH were used to plot the equilibrium energy diagram of the developed OLEDs, which is discussed in the subsequent section. The electron affinities (EA) of 3.4 eV for DiMeOBTTMS and 3.46 eV for DiMeOBTH were obtained by subtracting their optical band gaps from their ionisation energies (Table S4). These EA values suggest that electrons can be injected into films of DiMeOBTTMS and DiMeOBTH without energy barriers.

The injected holes can be effectively transported through the layers of DiMeOBTTMS and DiMeOBTH, with high hole mobility values (*µ*_*h*_) of 1.5 × 10^−4^ cm^2^ V^−1^ s^−1^ and 2.6 × 10^−4^ cm^2^ V^−1^ s^−1^ at the electric field (*E*) of 1.2 × 10^6^ V cm^−1^, respectively (Fig. 6b). Such hole mobility values are among the most exemplary for OLED materials.^68^ The electric field dependence parameter (*β*) for hole mobility of DiMeOBTTMS (3.5 × 10^−3^ (cm V^−1^)^0.5^) is lower than that of DiMeOBTH (4.5 × 10^−3^ (cm V^−1^)^0.5^) according to the Poole–Frenkel fitting (Fig. S9). This observation can be attributed either to less dispersive hole transport or lower trap effects in DiMeOBTTMS than in DiMeOBTH.^69^ The hole mobility values of DiMeOBTTMS and DiMeOBTH were obtained at different electric fields from the time-of-flight (TOF) experiment, which measured transit times (*t*_tr_) at different voltages for layers of DiMeOBTTMS and DiMeOBTH with thicknesses close to 2 µm (Fig. S10a and S11). The *t*_tr_ values were obtained from current transients plotted on log–log scales. The TOF measurements do not support electron transport in DiMeOBTTMS and DiMeOBTH, as no transit times were observed from corresponding current transients (Fig. S10). This outcome can be attributed to hole–electron recombination occurring at the interface between the light-emitting layer and the electron-transporting layer within the OLED structure, as discussed in greater detail below.

In addition, we studied charge-transporting properties of the commercially available TADF emitter 9-[4-(4,6-diphenyl-1,3,5-triazine-2-yl)phenyl]-*N*,*N*,*N*′,*N*′-tetraphenyl-9*H*-carbazole-3,6-diamine (DACT-II),^40^ the molecular structure of which is shown in Fig. S10. Bipolar transport of DACT-II was observed using the TOF method. At the relatively low electric field of 2.2 × 10^5^ V cm^−1^, DACT-II is characterised by higher electron mobility values (*µ*_e_) of 2.5 × 10^−4^ cm^2^ V^−1^ s^−1^ compared to its *µ*_h_ of 5 × 10^−5^ cm^2^ V^−1^ s^−1^ (Fig. 6b). The differences between the *µ*_e_ and *µ*_h_ values of DACT-II decrease with increasing *E*, approaching balanced hole and electron mobilities at high electric fields. This is possible because of the different *β* values of 1.6 × 10^−3^ (cm V^−1^)^0.5^ and 5 × 10^−3^ (cm V^−1^)^0.5^ for electron and hole mobilities of DACT-II, respectively (Fig. S10). Based on these measurements, we predict that emitter DACT-II can be used not only as the TADF emitter but also as the TADF host in OLEDs. This prediction is validated in the following section, where we studied the electroluminescent (EL) properties of DiMeOBTTMS and DiMeOBTH and attempted to develop doping-free white OLEDs.

### 2.5 Electroluminescence properties

The compounds DiMeOBTTMS and DiMeOBTH show great potential for use as OLED emitters due to their high PLQY values. To validate this potential, we fabricated single light-emitting layer (EML) OLEDs (devices A and B) based on neat EMLs of DiMeOBTTMS or DiMeOBTH, and host CBP-containing OLEDs (devices C and D) based on EMLs of DiMeOBTTMS (10 wt%):CBP or DiMeOBTH (10 wt%):CBP, respectively. In addition, we developed double-EML-based OLEDs (devices E and F) using two neat EMLs, where EML-1 is DiMeOBTTMS or DiMeOBTH, and EML-2 is DACT-II. Devices A and B had the multilayer structure of ITO/CuI/TAPC/TCTA/neat EML/TSPO1/TPBI/Ca/Al. Devices C and D had the multilayer structure ITO/CuI/TAPC(40 nm)/TCTA/host-based EML/PO-T2T/Ca/Al. Devices E and F had the multilayer structure of ITO/CuI/TAPC/TCTA/neat EML-1/neat EML-2/TPBI/Ca/Al. The functional layers of the devices were deposited using commercial materials, CBP as OLED host,^70^ copper(i) iodide (CuI) as the hole-injection material,^71^ 4,4′,4′′-tris(carbazole-9-yl)triphenylamine (TCTA) and 4,4′-cyclohexylidenebis[*N*,*N*-bis(4-methylphenyl)benzene amine] (TAPC) as hole-transporting materials,^72^ diphenylphosphine oxide-4-(triphenylsilyl)phenyl (TSPO1),^73^ (1,3,5-triazine-2,4,6-triyl)tris(benzene-3,1-diyl)tris(diphenylphosphine oxide) (PO-T2T)^74^ and 2,2′,2″-(1,3,5-benzenetriyl)-tris(1-phenyl-1-H-benzimidazole) (TPBi)^75^ as electron-transporting or hole-blocking materials. Additional details are provided in the experimental section of the SI.

**Fig. 7:**
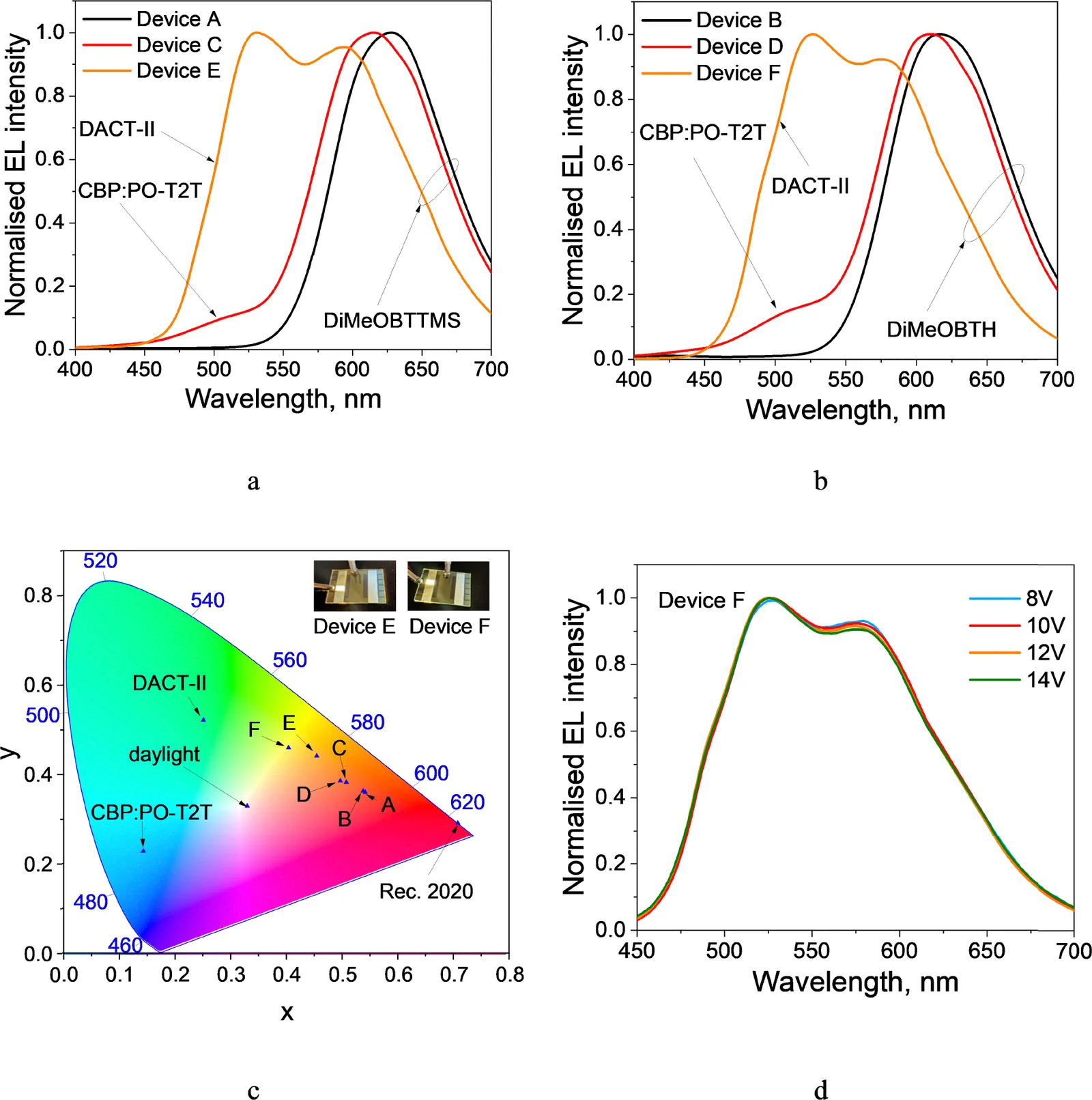
EL spectra (a) and (b) at 10 V and corresponding CIE1931 colour diagram (c) of devices containing DiMeOBTTMS (a) and (c) and DiMeOBTH (b) and (c). The CIE1931 colour diagram contains CIE1931 colour coordinates of high-energy TADF emitters CBP:PO-T2T and DACT-II taken using their PL spectra. EL spectra (d) of device F at different voltages.

The equilibrium energy diagrams of devices A–F indicate that hole–electron recombination zones are located within EMLs (Fig. S12). Consequently, the EL spectra correspond to the emission originating from the EMLs of the devices. For example, devices A and B emit red electroluminescence with spectra similar to the PL spectra of the corresponding emitters DiMeOBTTMS or DiMeOBTH. The neat EML-based devices A and B exhibit relatively low maximum external quantum efficiencies of 3.3% and 1.5%, respectively, which are characteristic of fluorescent OLEDs, wherein the energy of triplet excitons is dissipated as heat^76^ (Fig. 7a, b and Table 5). Device A reached the maximum EQE at a lower brightness of 55 cd m^−2^, compared with the maximum EQE of device B, observed at 1260 cd m^−2^. This observation can be explained by the lower sensitivity of hole mobility to electric fields in DiMeOBTTMS than in DiMeOBTH (Fig. 6b). Devices A and B are characterised by relatively high turn-on voltages of 5 V and 5.2 V because DiMeOBTTMS and DiMeOBTH reach high hole mobility values at high electric fields, respectively (Fig. 6b).

**Table 5:** EL output parameters

Device	Single or double EML	*V*_on_, V	*L*, cd m^−2^	CE, cd A^−1^	EQE, %	PE, lm W^−1^	*λ*_EL_, nm	CIE1931, (*x*, *y*)	CRI	CCT, K
A	DiMeOBTTMS	5	12 050	4.45	3.3	2.4	627	0.54, 0.36	—	1264
B	DiMeOBTH	5.2	7320	2.32	1.5	0.7	619	0.54, 0.36	—	1402
C	DiMeOBTTMS/CBP:PO-T2T	3.2	21 700	14.3	8.5	14	615, 511*	0.51, 0.38	—	1743
D	DiMeOBTH/CBP:PO-T2T	3.4	41 200	5.8	3.3	2	610, 513*	0.5, 0.38	—	1874
E	DiMeOBTTMS/DACT-II	5.4	7604	24.2	7.2	8.2	530, 594	0.4, 0.43	61	3840
F	DiMeOBTH/DACT-II	5.6	24 182	20	8.3	5.5	526, 577	0.38, 0.43	55	4345

The EL spectra of devices A and B remain the same at different voltages (Fig. S13). These spectra are relatively broad, showing full width at half maxima (FWHM) of 96 nm for device A and 98 nm for device B. This observation is typical of CT-emitting emitters.^77^ As a result, their CIE 1931 (*x*, *y*) chromaticity coordinates are relatively far from the television standard for red, *e.g.* Rec. 2020 (BT.2020) standard with CIE 1931 coordinates *x* of 0.708 and *y* of 0.292 (Fig. 7c and Table 5). On the other hand, efficient red emitters with a high FWHM can be used to develop white OLEDs.^77^ We proposed two approaches for white electroluminescence using emitters DiMeOBTTMS or DiMeOBTH. These approaches include the overlap between the red fluorescence of DiMeOBTTMS or DiMeOBTH and the high-energy TADF of the interface exciplex emitter CBP:PO-T2T, or the use of an additional EML based on the conventional TADF emitter DACT-II. These approaches not only enabled us to obtain whitish EL but also to enhance the EQE of DiMeOBTTMS- and DiMeOBTH-containing OLEDs.

**Fig. 8:**
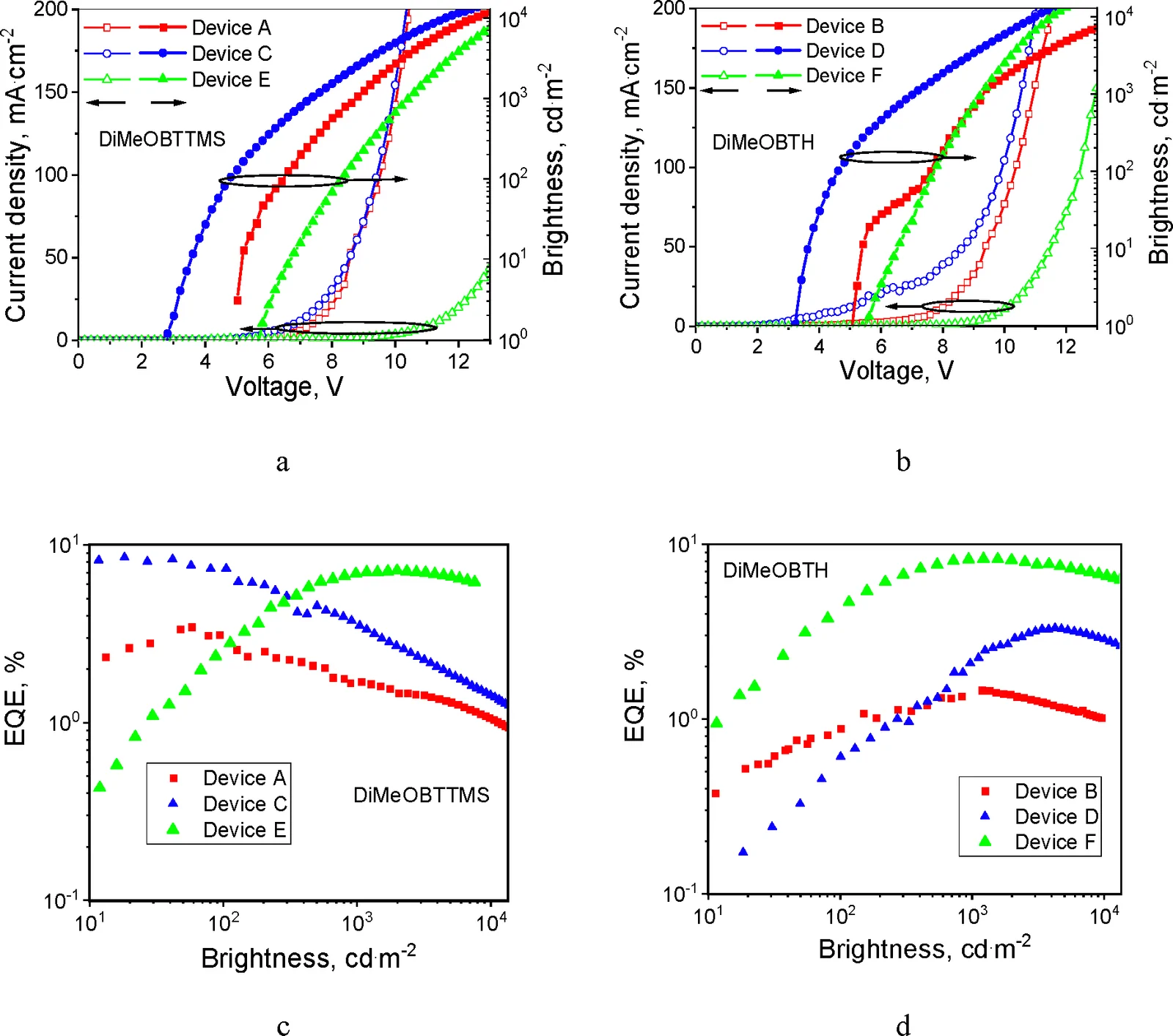
Current density–brightness–voltage (a) and (b) and EQE–brightness (c) and (d) dependences of devices containing DiMeOBTTMS (a) and (c) and DiMeOBTH (b) and (d).

Two approaches were used in the development of white OLEDs. The first approach to develop white OLEDs was based on mixing the red emissions of DiMeOBTTMS or DiMeOBTH with the interface-sky-blue exciplex emission formed between the host CBP and the electron-transporting layer PO-T2T of devices C and D (Fig. S12). The EL spectra of devices C and D are characterised by hypsochromic shifts of 39 nm and 43 nm, respectively, in the emission of DiMeOBTTMS or DiMeOBTH when dispersed in the host CBP, compared with the corresponding bands in the EL spectra of devices A and B. This behaviour is typical for EL spectra of donor–acceptor type red emitters, which are characterised by sensitivity to the polarity of the surrounding medium.^78^ FWHM values of 103 nm were obtained for spectra of devices C and D, which is wider than the value observed for devices A and B. In addition, the EL spectra of devices C and D are characterised by the high-energy band at approximately 450–550 nm. This band originates from the interface exciplex CBP:PO-T2T, which is characterised by efficient TADF, as studied elsewhere.^79,80^ However, white electroluminescent colours were not observed for these OLEDs (Fig. 7c). The overlap between the absorption spectra of DiMeOBTTMS or DiMeOBTH and the PL spectrum of the exciplex CBP:PO-T2T facilitates energy transfer *via* Förster resonance energy transfer (FRET).^81^ As a result, the maximum EQEs of 8.5% and 3.3% were achieved for devices C and D, respectively. Devices C and D exhibited low turn-on voltages of 3.2 V and 3.4 V, respectively, due to bipolar transport in CBP, which exhibits high hole and electron mobilities at low electric fields.^82^ In addition, the interfacial exciplex CBP:PO-T2T was included in the device structure of devices C and D (Fig. S13). The interfacial exciplex can act as an “active heterostructure”.^83^ The “active heterostructure” effectively reduces the energy barriers to charge injection, thereby lowering the turn-on voltages of devices C and D compared to those of devices A, B, E, and F, which contain similar functional layers but lack the interfacial exciplex (Fig. 8a, b and Table 5).

The second approach to develop white OLEDs was based on mixing the red emissions of DiMeOBTTMS or DiMeOBTH with the bluish-green emission of DACT-II in double light-emissive layers of devices E and F (Fig. S13). The EL spectra of devices E and F are characterised by spectral maxima in the high-energy region at 530 nm and 526 nm, respectively (Table 5). These peaks are consistent with the PL maximum of vacuum-deposited films of DACT-II, whose emission mechanism involves triplet states.^84^ The 594 nm and 577 nm emission bands in the EL spectra of devices E and F originate from DiMeOBTTMS or DiMeOBTH. The overlap of emission spectra from two emitters (DiMeOBTTMS or DiMeOBTH and DACT-II) caused spectral shifts that are not observed in the photoluminescence of neat films. Notably, the developed OLEDs with double light-emitting layers exhibit stable emission colours across different voltages, with CIE coordinates of (0.39, 0.43) and (0.38, 0.43) (Fig. 7c and Table 5). The EL colours of devices E and F meet the requirements of warm-white light,^85^ and are therefore classified as warm-white OLEDs. This claim is in good agreement with the colour rendering indexes (CRI) and correlated colour temperature (CCT) of corresponding devices (Table 5).

**Fig. 9:**
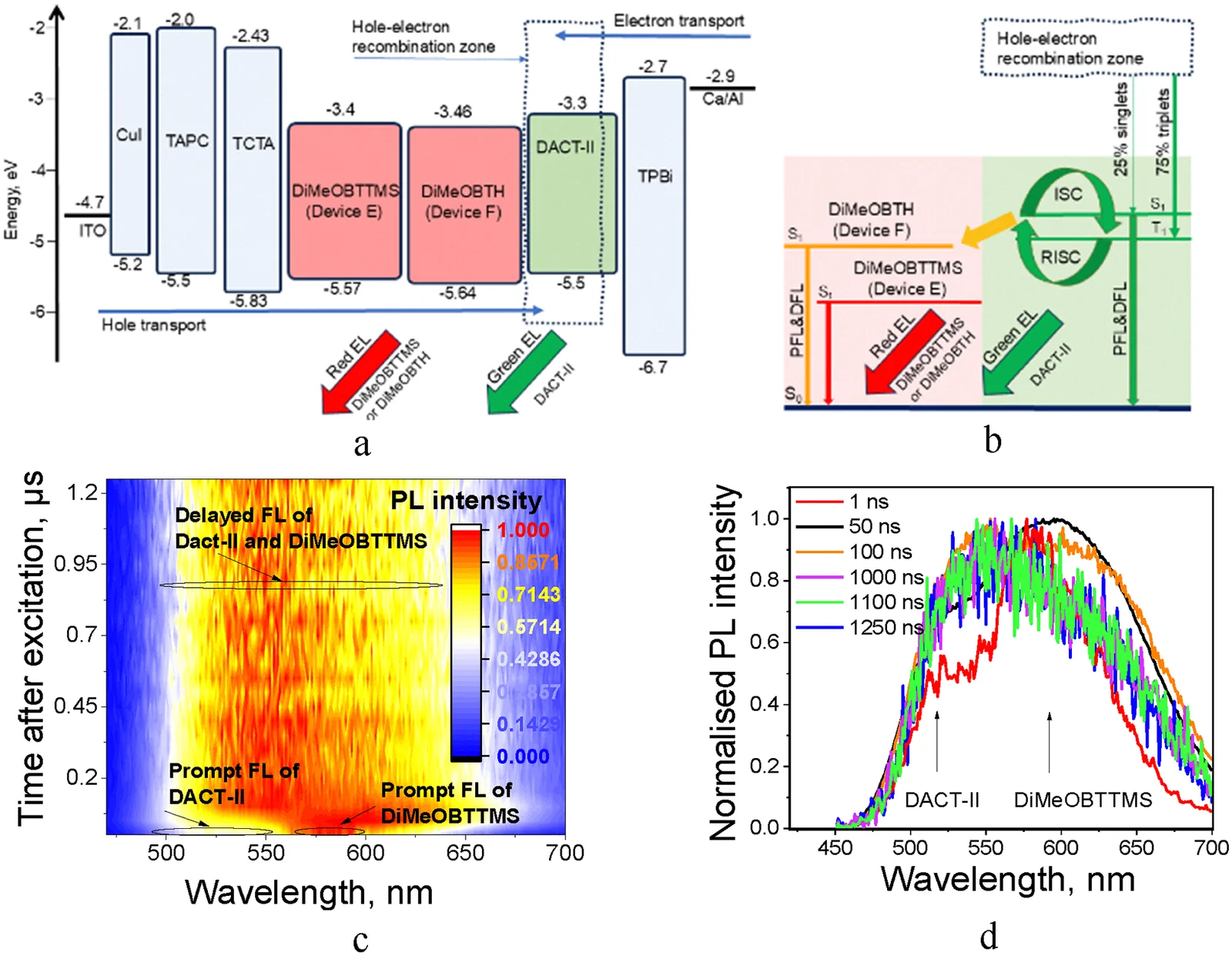
Equilibrium energy diagram (a) of devices E and F and schematic illustration of Jablonski energy diagram of DiMeOBTTMS/DACT-II and DiMeOBTH/DACT-II light-emitting systems (b). PL intensity as the 2D map (c) and the corresponding PL spectra (d) at different times after excitation of the DiMeOBTTMS/DACT-II film.

Due to the hole-transporting properties of DiMeOBTTMS and DiMeOBTH and higher electron mobilities of DACT-II than its hole mobilities at the same electric fields (Fig. 6b), the recombination zone of hole–electron pairs is located in the light-emitting layer DACT-II near the interface DiMeOBTTMS/DACT-II for device E or DiMeOBTH/DACT-II for device F (Fig. 9a). Then, energy transfer from DACT-II to DiMeOBTTMS or DiMeOBTH occurs (Fig. 9b), resulting in EL spectra with two representative green and red emission bands (Fig. 7a and b). Since the recombination zone of hole–electron pairs is not distributed within two ELMs, the shapes of the EL spectra of devices E and D remain practically unchanged at different voltages (Fig. 7d and Fig. S13). When the hole–electron recombination zone is distributed across double light-emitting layers, it typically leads to strong variations in EL colours of white OLEDs with increasing applied voltage.^85,86^ Thus, the proposed approach concerns the location of the hole–electron recombination zone within the light-emitting layer of DACTII. The TADF properties of DACTII with small singlet–triplet splitting allow the harvesting of both singlet and triplet excitons *via* reverse intersystem crossing (RISC), as shown in Fig. 9b. Part of the singlet excitons leaks to the light-emitting layers DiMeOBTTMS or DiMeOBTH*via* singlet–singlet energy transfer. As a result, prompt and delayed fluorescence (PFL and DFL) is observed not only for green DACTII emission but also for red DiMeOBTTMS or DiMeOBTH emission (Fig. 9b). This exciton harvesting mechanism leads to the voltage independent EL spectra of devices F and E. Nonetheless, a small difference in the corresponding EL spectra of devices F and E at different voltages can be observed. This observation is due to the extension of the hole–electron recombination zone within the DACTII light-emitting layer, leading to a slight increase in DACTII emission intensity relative to DiMeOBTTMS or DiMeOBTH (Fig. 7 and Fig. S13). To support the statement on the singlet–singlet energy transfer from DACT-II to DiMeOBTTMS, we recorded time-resolved emission spectra (TRES) of the corresponding EML (Fig. 9c, d and Fig. S14). The green band at *ca.* 530 nm is characterised by short- and long-lived fluorescence (prompt FL and delayed FL) caused by TADF of DACT-II.^40^ In addition, long-lived emissions are observed for DiMeOBTTMS, which were not identified in their photophysical investigations. These long-lived fluorescence processes are a good indication of energy transfer from DACT-II to DiMeOBTTMS. As a result, not only is stable warm-white electroluminescence across different voltages achieved, but also maximum EQE values of 7.2% and 8.3% were achieved for devices E and D, respectively, from doping-free layers DiMeOBTTMS or DiMeOBTH and DACT-II. In comparison to devices A–D, devices E and F showed higher *V*_on_ values and lower current densities at the same driving voltages (Fig. 8a and b). This result can be explained by the higher total thickness of 150 nm, compared with 120 nm in other devices, but not by the device structure. Enhanced EQEs and tuning of EL colours are predicted by replacing the green TADF emitter DACT-II with a blue TADF emitter and further optimising the thicknesses of the OLED functional layers. These investigations fall outside the scope of the current study. Nonetheless, compounds DiMeOBTTMS and DiMeOBTH can be selected for the development of warm-white OLEDs with stable colour coordinates at different EL brightnesses (Fig. 7).

## 3 Conclusions

The novel donor–acceptor compounds DiMeOBTTMS and DiMeOBTH based on dimethoxybenzothiadiazole were synthesised, and their properties were investigated both experimentally and theoretically. These molecules exhibit emission in the red region and are characterised by pronounced fluorescence with PLQY above 90% in toluene and CH_2_Cl_2_. Electrochemistry of both compounds in CH_2_Cl_2_, compared to the cyclic voltammetry of their parent compound BTTMS, suggests increased coupling between the central acceptor BT core and adjacent thiophene donor units which is rationalised by planarisation of the Th–BT–Th unit, due to non-covalent interactions (NCI). The effect of S⋯O and N⋯H NCI is confirmed by theoretical calculations. Using an exciplex host, hyperfluorescence from DiMeOBTTMS and DiMeOBTH was observed, yielding an EQE of 8.5%. The combination of dual emission from the newly synthesised compounds together with the TADF green emitter DACT II enabled the fabrication of OLEDs with warm-white emission. The best white-emitting device demonstrated a maximum current efficiency of 24.25 cd A^−1^, a power efficiency of 7.15 lm W^−1^, and an external quantum efficiency of 8.23%. The developed devices with double light-emitting layers exhibit stable warm-white electroluminescent colours across different voltages, with CIE coordinates of (0.39, 0.43) and (0.38, 0.43).

## Author contributions

This manuscript was written with contributions of all authors. All authors have given approval to the final version of the manuscript.

## Conflicts of interest

There are no conflicts to declare.

## Supplementary Material

TC-014-D6TC01121G-s001

## Data Availability

The data supporting this article has been included as part of the supplementary information (SI). Supplementary information is available. See DOI: https://doi.org/10.1039/d6tc01121g.
