# Supplementary material for: Efficiently red-emitting dimethoxybenzothiadiazoles for organic doping-free devices enabling stable warm-white electroluminescence
Source: J Mater Chem C Mater. 2026 Jul 16;14(36):16900–12. doi: 10.1039/d6tc01121g (PMC13613325; doi:10.1039/d6tc01121g)
Supplement: Supplementary file 1 [file TC-014-D6TC01121G-s001.pdf]

Supplementary Information (SI) for Journal of Materials Chemistry C.
This journal is © The Royal Society of Chemistry

Supporting Information

Efficiently red-emitting dimethoxybenzothiadiazoles for organic doping-free devices enabling stable warm-white electroluminescence

Stepan Kutsiy,[1] Lyudmyla Kanibolotska,[2] Alexander Kanibolotsky,[2,3] Mykhailo Shchetinin,[1] Khrystyna Ivaniuk,[1] Pavlo Stakhira,[1] Asta Dabuliene,[4] Dmytro Volyniuk,[4*] Joseph Cameron,[2] Juozas V. Grazulevicius,[4*] Iryna Yaremchuk,[1*] Peter J. Skabara[2*]

[1] *Department of Electronic Engineering, Lviv Polytechnic National University, Sviatoho Yura sq. 1, Lviv 79013, Ukraine. E-mail stepan.a.kutsii@lpnu.ua*

[2] *School of Chemistry, University of Glasgow, Glasgow, G12 8QQ, United Kingdom*

[3] *Institute of Physical-Organic Chemistry and Coal Chemistry, 02160 Kyiv, Ukraine*

[4] *Department of Polymer Chemistry and Technology, Kaunas University of Technology, Barsausko 59, LT-51423, Kaunas, Lithuania. E-mails: dmytro.volyniuk@ktu.lt, juozas.grazulevicius@ktu.lt*

Contents

1 General information 2
2 The synthesis of diMethoxyRedBT compounds 6
3 DFT calculations of diMethoxyRedBT 14
4 Thermal properties 15
5 Photophysical characteristics and parameters 16
6 Energy level and charge transport data 23
7 OLEDs 27

1 General information

All the commercially available reagents were purchased from Sigma-Aldrich. Tetrakis(triphenylphosphine)palladium(0) ($Pd(PPh_3)_4$) was prepared according to known procedure [1].

Dry solvents were obtained from a solvent purification system (SPS 400 from Innovative Technologies) using alumina as the drying agent.

Spectroscopic Measurements and Analyses

UV-Vis Spectroscopy: UV-Vis spectra were recorded using a Shimadzu UV-2600 Spectrophotometer in the range 200-800 nm, using a 0.1 and 1 cm path length quartz cells. Solution state measurements were performed using dry solvent.

Fluorescence Spectra were obtained using HORIBA Duetta's dual fluorescence and absorbance spectrometer and FS5 Spectrofluorometer from Edinburgh Instruments.

Fluorescence decay curves were recorded using the Edinburgh Instruments FLS980 spectrometer equiped by a PicoQuant laser PDL 820 with the emission wavelength of 374 nm.

The fittings of their fluorescence decay curves were provided using software of the Edinburgh Instruments FLS980 spectrometer, according to the following formulas

R(t) =A1·exp(-t/τ1) (S1)

R(t) =A1·exp(-t/τ1) + A2·exp(-x/τ2) (S2)

Where, t is time, A1, and A2 are the fractional intensities of the time components τ1, and τ2, respectively. If the error fitting factor χ^2 was not in the range 1□□2<1.3 using formula (S1), formula (S2) was selected. The fitting data were tabulated.

NMR Spectroscopy: NMR analysis was conducted on a Bruker AVIII 400MHz NMR or a Bruker DRX 500 apparatus. Chemical shifts are given in ppm; all *J* values are in Hz. The spectra were calibrated by residual proton shift of the $CDCl_3$ (7.26 ppm) for ^1H NMR and the carbon nucleus shift of 77.16 ppm for ^{13}C NMR spectra respectively [2]. The splitting of the proton signals in ^1H NMR is denoted as m (multiplet), s (singlet), d (doublet), dd (doublet of doublets), ddd (doublet of doublets of doublets), t (triplet), td (triplet of doublets).

Mass Spectrometry: The mass spectrometry utilises **Shimadzu MALDI-TOF (MALDI8030)** for rapid analysis of high-mass species up to m/z 500000.

Thermogravimetric analysis (TGA) was performed using a PerkinElmer Thermogravimetric Analyzer TGA7 under a constant flow of argon. Differential scanning calorimetry (DSC) was recorded on a TA Instruments DSC QC1000 under nitrogen gas.

Melting points were obtained on a Cole-Parmer Stuart SMP50 automatic melting point apparatus.

Elemental Analysis was conducted on an Exeter CE-440 Elemental Analyser.

Cyclic voltammetry was recorded on a CH Instruments 660A electrochemical workstation with *iR* compensation at a scan rate of 100 mV∙s^{-1} using anhydrous dichloromethane as the solvent. The electrodes were glassy carbon, platinum wire and silver wire as the working, counter and pseudo-reference electrodes, respectively. All solutions were degassed with argon and contained the analyte in concentrations of *ca.* 10^{-4} M, together with 0.1 M tetrabutylammonium hexafluorophosphate as the supporting electrolyte. All measurements were referenced against the $E_{1/2}$ of the

Fc/Fc^+ redox couple. The HOMO (LUMO) was calculated by subtracting the half-wave potential of oxidation (reduction) from −4.8 eV [3].

Device fabrication. To demonstrate the potential of compounds DiMeOBTTMS and DiMeOBTH as red emitters and co-emitter for white OLED, six types of OLED devices were fabricated. The devices A-F have the following structures:

A)

ITO/CuI(8nm)/TAPC(40nm)/TCTA(10nm)/DiMeOBTTMS(30nm)/TSPO1(10nm)/TPBI(30nm)/Ca(15 nm)/Al(120nm).

B)

ITO/CuI(8nm)/TAPC(40nm)/TCTA(10nm)/DiMeOBTH(30nm)/TSPO1(10nm)/TPBI(30nm)/Ca(15nm)/Al(120nm).

Host CBP-containing OLEDs

□)

ITO/CuI(8nm)/TAPC(40nm)/TCTA(10nm)/DiMeOBTTMS(ca.10wt.%):CBP(30nm)/PO-T2T(40nm)/Ca(15nm)/Al(120nm).

D)

ITO/CuI(8nm)/TAPC(40nm)/TCTA(10nm)/DiMeOBTH(ca.10wt.%):CBP(30nm)/PO-T2T(40nm)/Ca(15 nm)/Al(120nm).

E)

ITO/CuI(8nm)/TAPC(40nm)/TCTA(10nm)/DiMeOBTTMS(20nm)/DACT-II(20nm)/TPBI(30nm)/Ca(15nm)/Al(120nm).

F)

ITO/CuI(8nm)/TAPC(40nm)/TCTA(10nm)/DiMeOBTH(20nm)/DACT-II(20nm)/TPBI(30nm)/Ca(15nm)/Al(120nm).

Devices were fabricated on glass substrates coated with indium tin oxide (ITO) and 100 nm thick. Before evaporation, the substrates were washed with distilled water, then with acetone, followed by sonication in acetone. The layers were thermally deposited using a vacuum chamber at a pressure of 10-5 Torr. The fabricated devices had a pixel area of 2 × 2 mm. The devices were characterized using a Hewlett-Packard Semiconductor parameter analyser 4145A and a calibrated photodiode. The spectra were measured on an Ocean Optics USB2000 spectrometer.

2 The synthesis of diMethoxyRedBT compounds

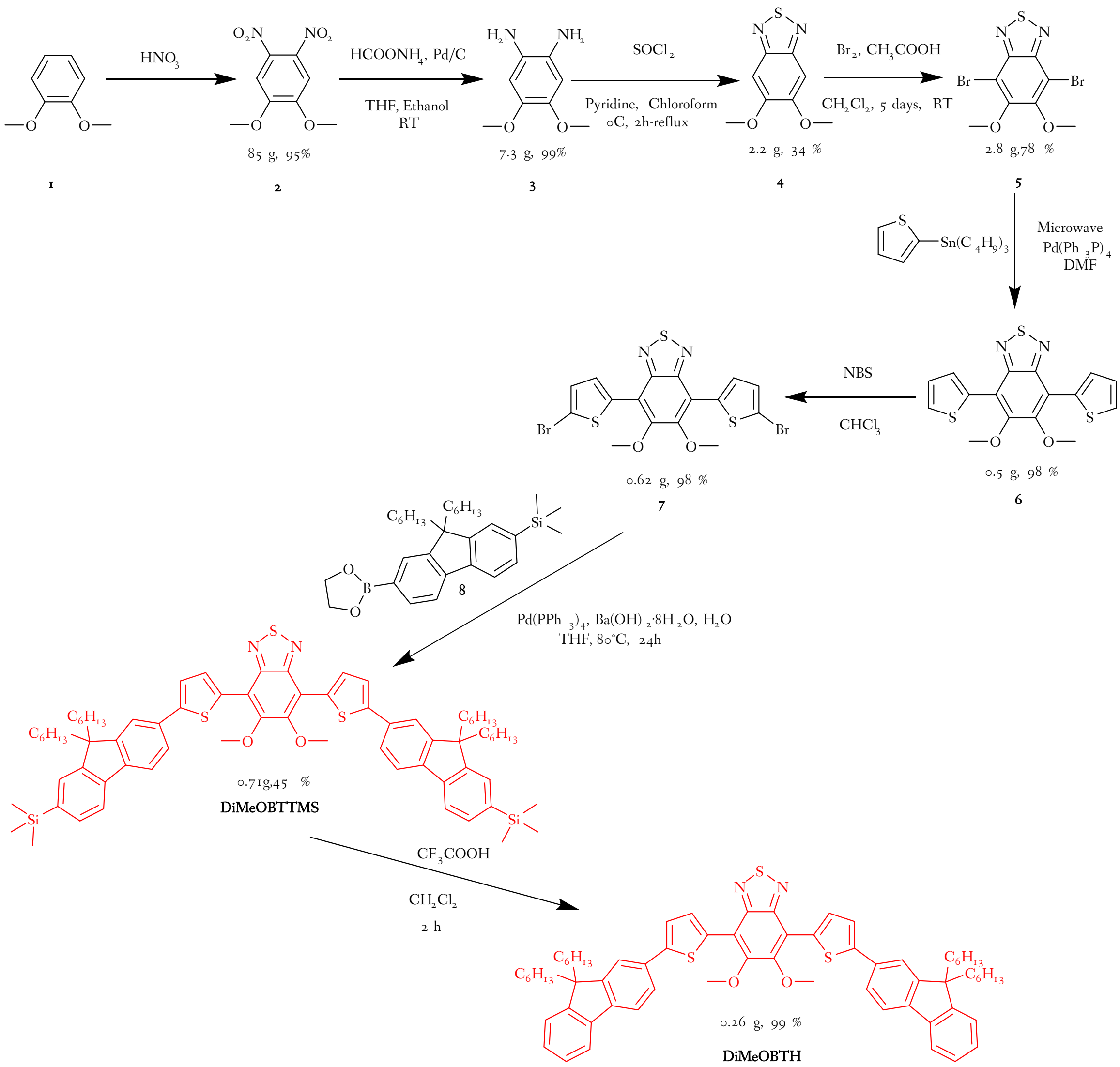

Scheme S1. The synthetic route of diMethoxyRedBT compounds.

Compound 2. ***1,2-Dimethoxy-4,5-dinitrobenzene.*** In a two neck 1 L round bottom flask 1,2- dimethoxybenzene **(1)** (50 mL, 391 mmol) was added to concentrated nitric acid (70%, 300 mL) dropwise (over 15 min) under vigorous stirring at room temperature. (CAUTION! the reaction is very exothermic upon addition of 1,2-dimethoxybenzene and reflux conditions are reached. A large amount of nitrogen dioxide is released during the reaction). This reaction was stirred for approximately 20

min until it reached room temperature. Ice-water slowly was added to the reaction, and a green amorphous solid product was recovered by vacuum filtration and washed with copious amount of water. Ice-water slowly was added to the reaction, and a green amorphous solid product was recovered by vacuum filtration and washed with water. Yield 85 g, 95% [4].

Compound 3. ***1,2-Diamino-4,5-dinitrobenzene.*** 1,2-Dimethoxy-4,5-dinitrobenzene **(2)** (10 g, 43.8 mmol) and Pd/C catalyst (1.5 g, 15%) were dispersed in ethanol (450 mL). Hydrazine monohydrate (21.3 mL, 438 mmol) was added dropwise to the mixture over an ice-water bath. The mixture was refluxed for 30 min at 80 °C. Upon completion, the mixture was filtered through Celite, and the residual material was rinsed with small portions of hot ethanol. The filtrate was collected and the off-white, crystalline product was obtained by removing the solvent. The product was dried and stored under nitrogen (7.3 g, 99% yields) [5].

Compound 4. ***4,7-Dimethoxy-2,1,3-benzothiadiazole.*** To a mixture of the 1,2-Dimethoxy-4,5-diaminobenzene **(3)** (7 g, 41.6 mmol) and pyridine (5.0 mL, 62.1 mmol) in anhydrous chloroform (100 mL) was added dropwise thionyl chloride (4.6 ml, 1.03 mol) at 0 °C for 20 min. The mixture was heated at the refluxing temperature for 2 h under an argon atmosphere. After the reaction mixture cooled down to room temperature, it was quenched by addition of 1 M sodium hydrogen carbonate aqueous solution (100 mL) and acidified by addition of 1.2 M hydrochloric acid aqueous solution (pH 4∼5). The resulting mixture was extracted with dichloromethane. The

combined organic layer was washed with brine and water, dried over anhydrous magnesium sulphate, and evaporated in vacuo to dryness. The residue was purified by silica gel column chromatography eluting with hexane/ethyl acetate (4:1, v/v) to give BTD(OMe) in 34% (2.2 g). An analytical sample was obtained by recrystallization from hexane/chloroform as colourless needles. Mp 160–161 °C [6].

Compound 5. ***4,7-Dibromo-5,6-dimethoxy-2,1,3-benzothiadiazole.*** To a solution of 4,7-Dimethoxy-2,1,3-benzothiadiazole **(4)** (2.0 g, 10.2 mmol) in dry dichloromethane (190 mL) was added acetic acid (93 mL) and then was added bromine (3.65 ml, 71.4 mmol) under an argon atmosphere. The mixture was stirred at room temperature for 24 h under dark conditions. After the reaction mixture was quenched by addition of cold water and 1 M aqueous solution of sodium hydrogen sulphite, it was extracted with chloroform. The combined organic layer was washed with 1 M aqueous solution of sodium hydrogen sulphite and water, dried over anhydrous magnesium sulphate, and evaporated in vacuo to dryness. The residue was purified by silica gel column chromatography eluting with hexane/CH_2Cl_2 (1:1, v/v) to give 4,7-dibromo-5,6-dimethoxy-2,1,3-benzothiadiazole in 78% yield (2.8 g). An analytical sample was obtained by recrystallization from hexane/chloroform as colourless needles [6].

Compound 6. ***5,6-Dimethoxy-4,7-di(thiophen-2-yl)benzo[c][1,2,5]thiadiazole.*** 4,7-Dibromo-5,6-dimethoxy-2,1,3-benzothiadiazole **(5)** (500 mg, 1.412 mmol, 1.0 eq.) and tetrakis(triphenylphosphine) palladium (0) (1.2 g, 1.04 mmol, 0.7 eq.) were charged to a 20 ml high pressure microwave vial with a stirred bar. The vial was then sealed with

a septum and evacuated, and purged with Ar. After freshly distilled DMF was added (10 ml), and then 2-(tributylstannyl)thiophene (2.43 ml, 7.654 mmol, 3.0 eq.) was added. The solution was flushed for 20 minutes with Ar before the reaction was heated gradually to 80° C for 5 minutes, after to 100° C for 5 minutes and to 120° C for 30 minutes. The reaction mixture was allowed to cool to room temperature when the precipitate appeared. The precipitate was filtered and washed with methanol. After then, the precipitate was dissolving in CH_2Cl_2 and passed through silica plug. After evaporation of solvent the resulting orange powder was purified on silica gel, eluting with CH_2Cl_2:hexane 1:10, 1:5, to afford 5,6- dimethoxy-4,7-di(thiophen-2-yl)benzo[c][1,2,5]thiadiazole as an orange powder (500 mg, 98%) [7].

Compound 7. *4,7-Bis(5-bromothiophen-2-yl)-5,6-dimethoxybenzo[c][1,2,5]thiadiazole.* *5,6-Dimethoxy-4,7-di(thiophen-2-yl)benzo[c][1,2,5]thiadiazole* **(6)** (500 mg, 1.39 mmol, 1.0 eq.) was dissolved in CH_2Cl_2 (50 ml) and the reaction flask covered in aluminium foil to ensure the mixture remained dark. N-Bromosuccinimide (543 mg, 3.05 mmol, 2.2 eq.) was added in one portion, after 2 drops of acetic acid were added and the mixture stirred at r.t. under Ar for 72 h. After this time, the reaction mixture was filtered and the resultant orange solid washed with distilled water (20 ml), methanol (150 ml) and chloroform (4 ml). The red powder was dried under vacuum overnight and crystallised from DMF to afford 620 mg, 98% of the product [7].

DiMeOBTTMS. ***4,7-bis[5-(*****9,9-Dihexyl-7-(trimethylsilyl)-9H-fluoren-2-yl*****)-2-thienyl]-5,6-dimethoxy-2,1,3-benzothiadiazole.*** Compound 7 (620 mg, 1.35 mmol, 1.0 eq.), (9,9-Dihexyl-7-(trimethylsilyl)-9H-fluoren-2-yl)boronic acid pinacol ester (1.162 g, 3.38 mmol, 2.5 eq.) [8], tetrakis(triphenylphospine)palladium(0) (313 mg, 0.271 mmol, 0.2 eq.) and barium hydroxide octahydrate (2.13 g, 6.77 mmol, 5 eq.) were charged to a reaction flask, evacuated and purged with Ar). Anhydrous THF (25 ml) and degassed, distilled water (1.8 ml) were added and the mixture heated to reflux for 40 h. The mixture was then cooled, diluted with water (100 ml), then extracted with dichloromethane. The combined organic extracts were dried with $MgSO_4$ and concentrated under vacuum to a dark red residue. Silica gel column chromatography, eluting with dichloromethane/hexane 1/12 to 1/10 (0.71 g, 45 % yield). Mp 93-95 °C. MALDI calc for [M($C_{72}H_{92}N_2O_2S_3Si_2$)] 1169.89, found 1169.79.

^{1}H NMR ($CDCl_3$, 400 MHz, ppm): 8.68 (1H, d, ^3J = 4.0 Hz), 7.76 – 7.67 (4H, m), 7.53 (1H, d, ^3J = 4.0 Hz), 7.51 (1H, dd, ^3J = 7.5 Hz, ^4J = 0.9 Hz), 7.48 (1H, s), 4.15 (3H, s), 2.03 (4H, t, ^3J = 8.24 Hz), 1.23 – 0.98 (12H, m), 0.77 (6, t, ^3J = 6.92 Hz), 0.84 – 0.58 (4H, m), 0.33 (9H, s).

^{13}C NMR ($CDCl_3$, 400 MHz, ppm): 152.23, 151.94, 150.97, 150.25, 146.87, 141.39, 141.14, 139.41, 133.39, 133.37, 132.27, 132.03, 127.73, 125.02, 123.26, 120.37, 119.15, 117.34, 60.97, 55.30, 40.33, 31.52, 29.72, 23.83, 22.64, 14.15, 0.72.

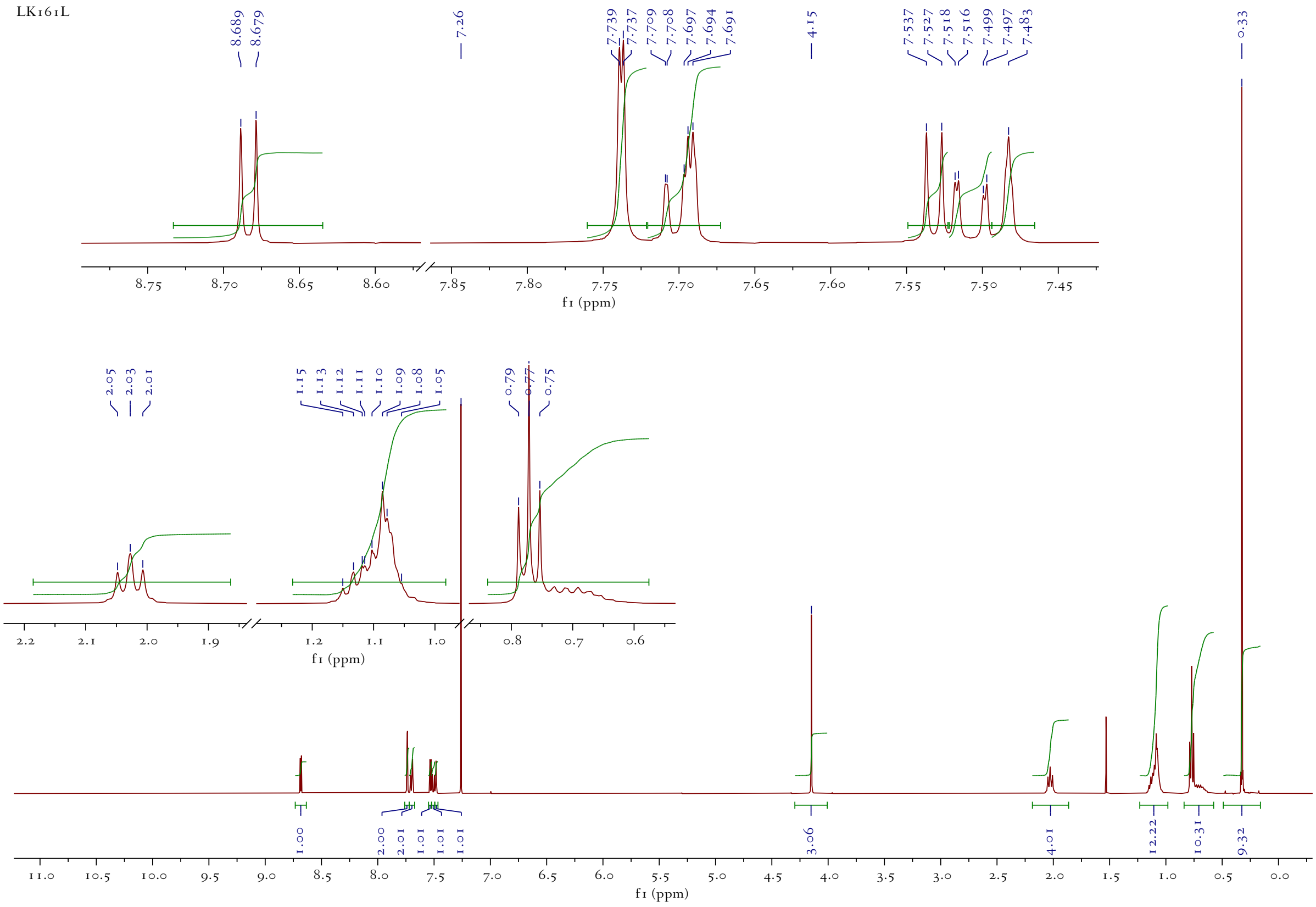

Figure S1. *^{1}H NMR spectrum of 4,7-bis[5-(9,9-Dihexyl-7-(trimethylsilyl)-9H-fluoren-2-yl)-2-thienyl]-5,6-dimethoxy-2,1,3-benzothiadiazole (DiMeOBTTMS).*

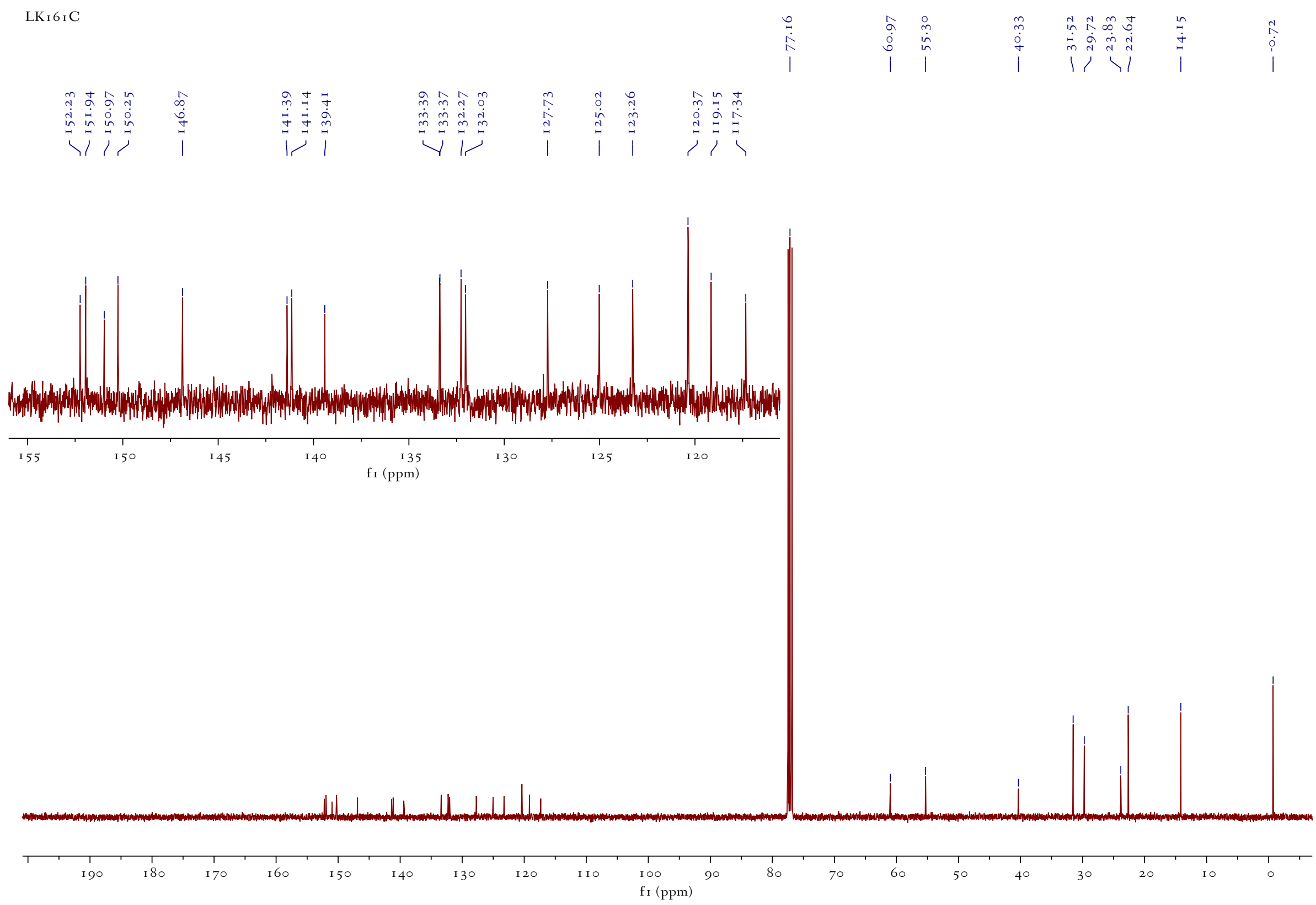

Figure S2. *^{13}C NMR spectrum of 4,7-bis[5-(9,9-Dihexyl-7-(trimethylsilyl)-9H-fluoren-2-yl)-2-thienyl]-5,6-dimethoxy-2,1,3-benzothiadiazole (DiMeOBTTMS).*

DiMeOBTH. ***4,7-bis[5-(9,9-Dihexyl-9H-fluoren-2-yl)-2-thienyl]-5,6-dimethoxy-2,1,3-benzothiadiazole.*** To a solution of the corresponding trimethylsilylated compound (DiMeOBTTMS) in dichloromethane (100 ml per 1 g of the compound) trifluoroacetic acid (1/10 of CH_2Cl_2 volume) was added, and the mixture stirred for another 2 h. Aqueous work up afforded the 4,7-bis[5-(9,9-Dihexyl-9H-fluoren-2-yl)-2-thienyl]-5,6-dimethoxy-2,1,3-benzothiadiazole as red solid, after precipitation dichloromethane/methanol (0.26 g, 99 % yield). Mp 90-92 °C. MALDI calc for [M(C66H76N2O2S3)] 1025.53, found 1025.68.

^{1}H NMR ($CDCl_3$, 400 MHz, ppm): 8.68 (1H, dd, ^{3}J = 4.0 Hz, ^{4}J = 0.5 Hz), 7.80 – 7.65 (4H, m), 7.54 (1H, d, ^{3}J = 4.0 Hz), 7.42 – 7.29 (3H, m), 4.15 (3H, s), 2.22 – 1.85 (4H, m), 1.24 – 0.96 (12H, m), 0.77 (6, t, ^{3}J = 6.96 Hz), 0.84 – 0.56 (4H, m).

^{13}C NMR ($CDCl_3$, 400 MHz, ppm): 152.24, 151.72, 151.10, 150.97, 146.85, 141.16, 140.78, 133.38, 133.25, 132.26, 127.32, 126.99, 125.03, 123.26, 123.04, 120.28, 120.24, 119.89, 117.34, 60.98, 55.37, 40.59, 31.65, 29.86, 23.90, 22.73, 14.16.

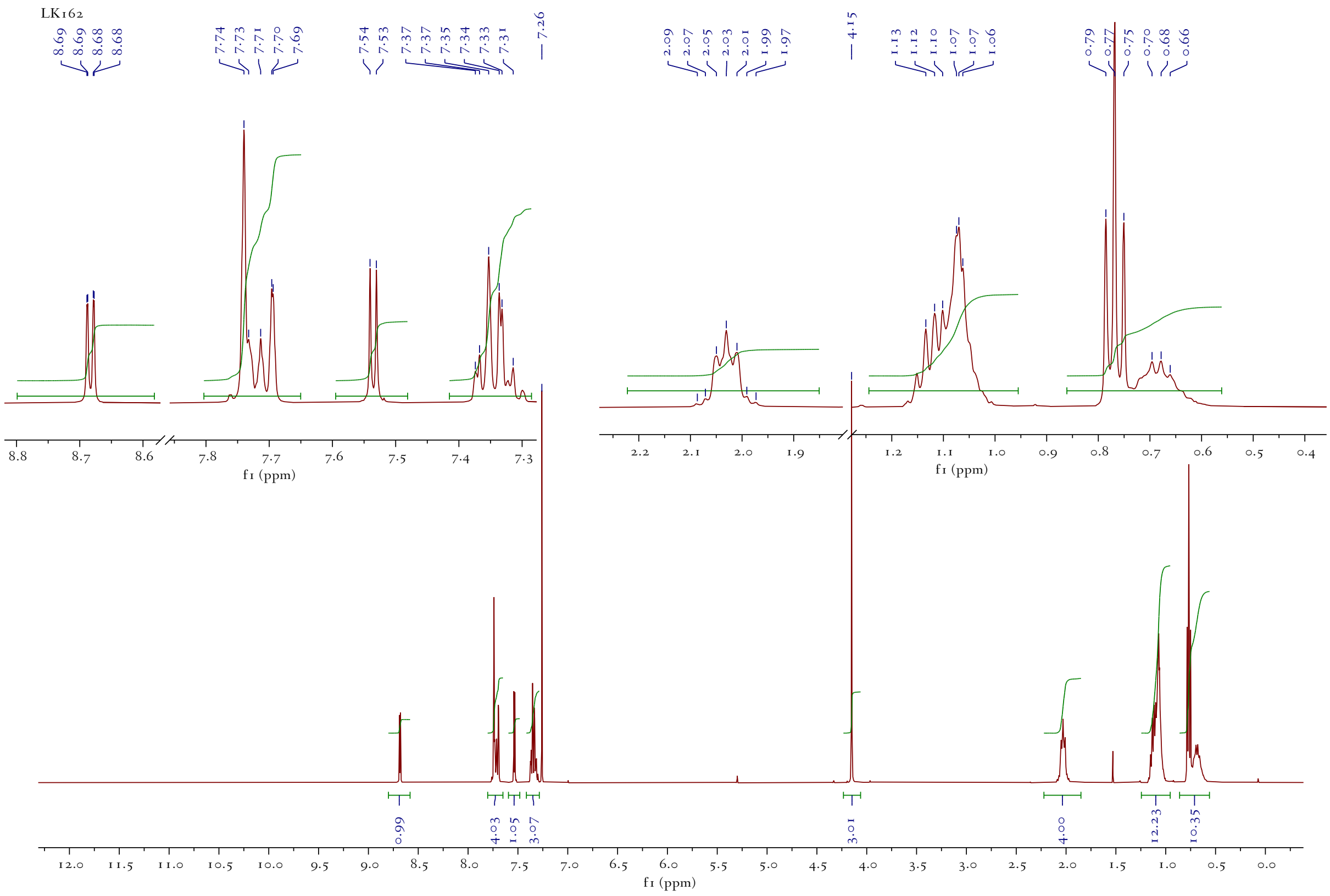

Figure S3. 1H NMR spectrum of 4,7-bis[5-(9,9-Dihexyl-9H-fluoren-2-yl)-2-thienyl]-5,6-dimethoxy-2,1,3-benzothiadiazole (DiMeOBTH).

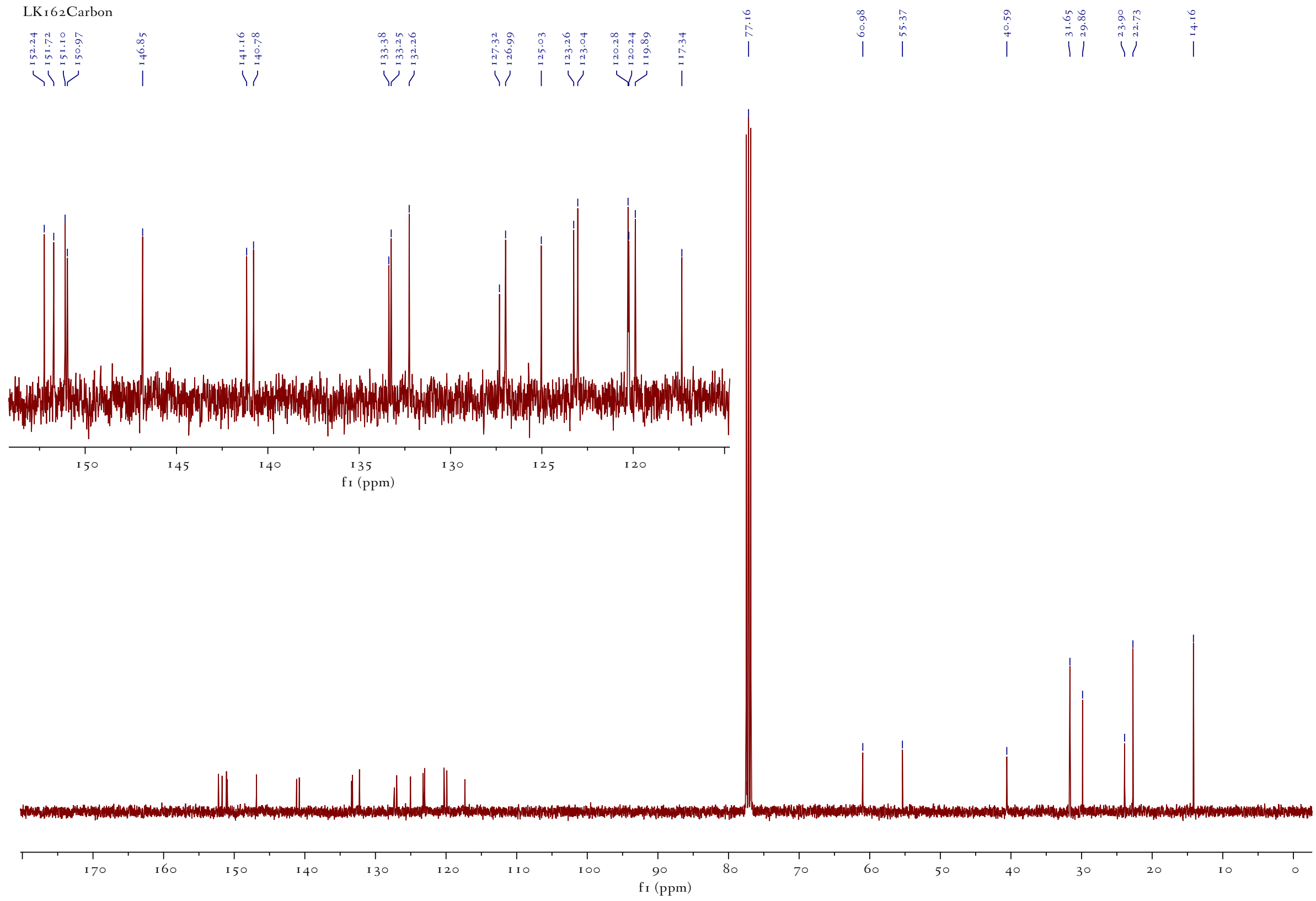

Figure S4. ^{13}C NMR spectrum of 4,7-bis[5-(9,9-Dihexyl-9H-fluoren-2-yl)-2-thienyl]-5,6-dimethoxy-2,1,3-benzothiadiazole (DiMeOBTH).

3 DFT calculations of diMethoxyRedBT

All density functional theory (DFT) and time-dependent density functional theory (TDDFT) calculations were carried out using Gaussian 16 software [9]. Hexyl groups of the molecules studied were shortened to methyl to reduce computational cost. DFT calculations were carried out using PBE0 functional with 6-31G(d,p) basis set. Frequency calculations showed no imaginary frequencies, confirming that the optimised structure was at a minimum on the potential energy surface. TDDFT calculations were carried out using the Tamm-Dancoff approximation, using PBE0

functional and 6,31G(d,p) basis set. Spatial overlap of excitations from TDDFT output was determined using the approach described by Peach *et al.* [10].

4 Thermal properties

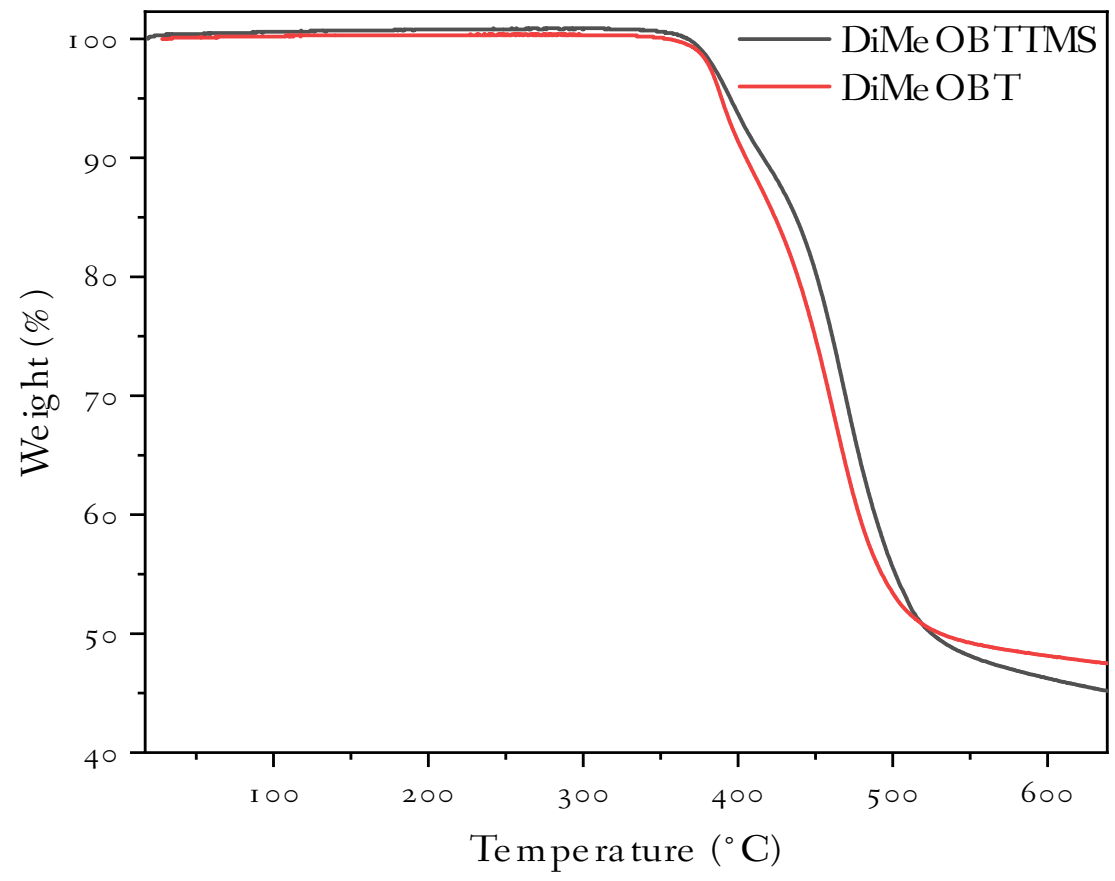

a)

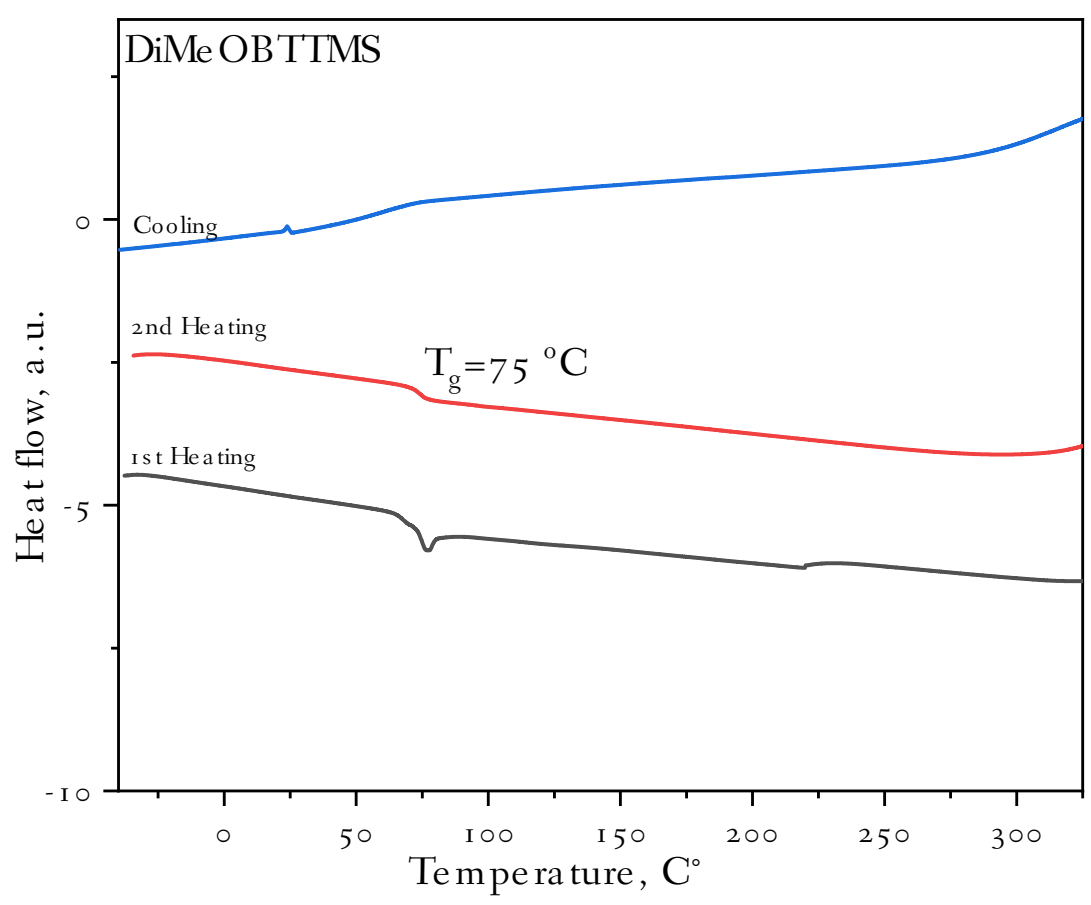

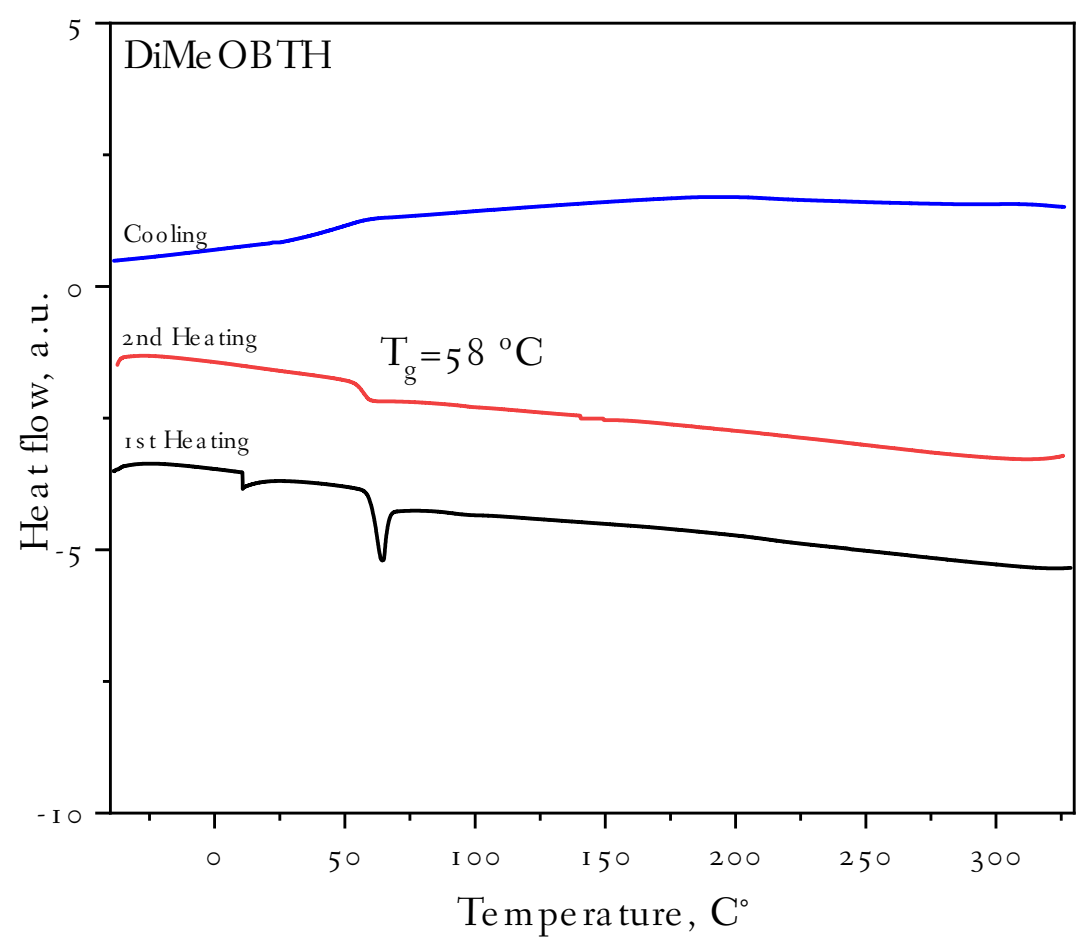

b) c)

Figure S5. TGA (a) and DSC (b, c) curves of compounds DiMeOBTTMS and DiMeOBTH.

5 Photophysical characteristics and parameters

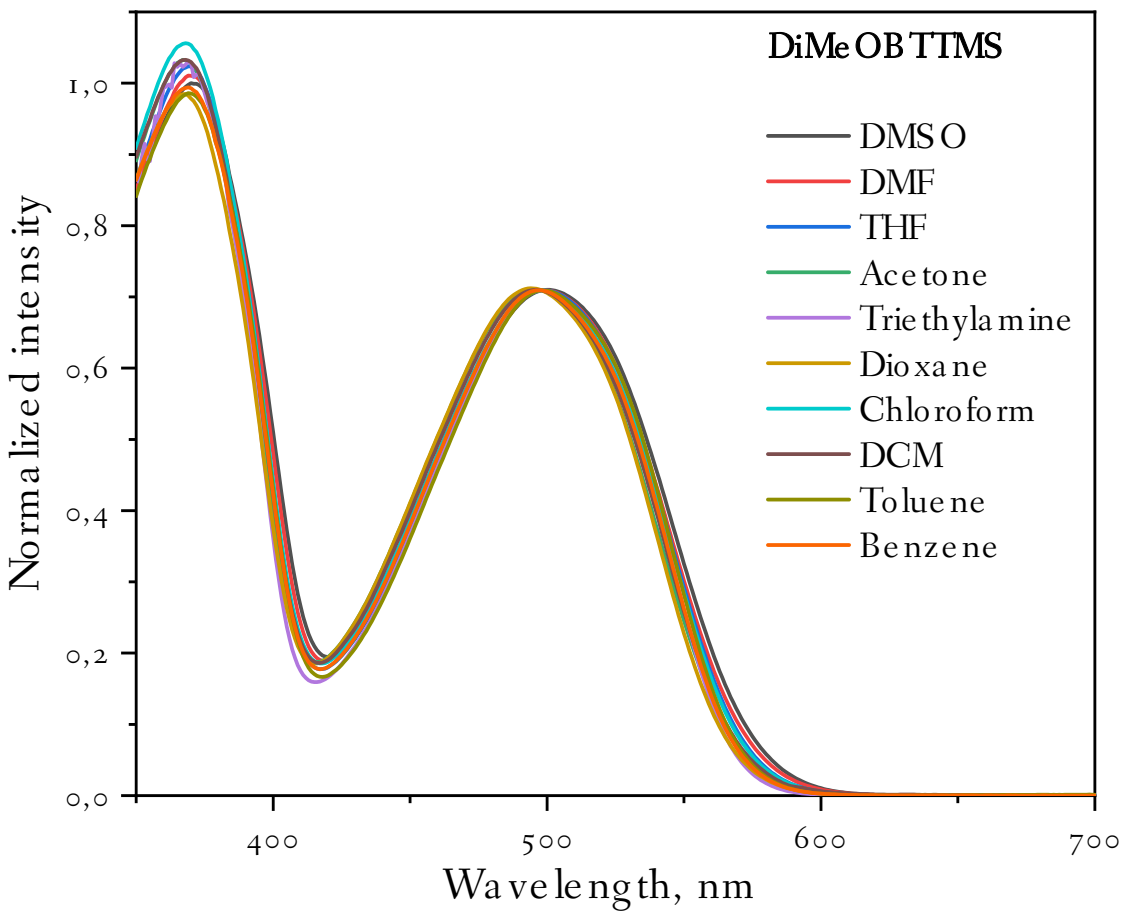

a

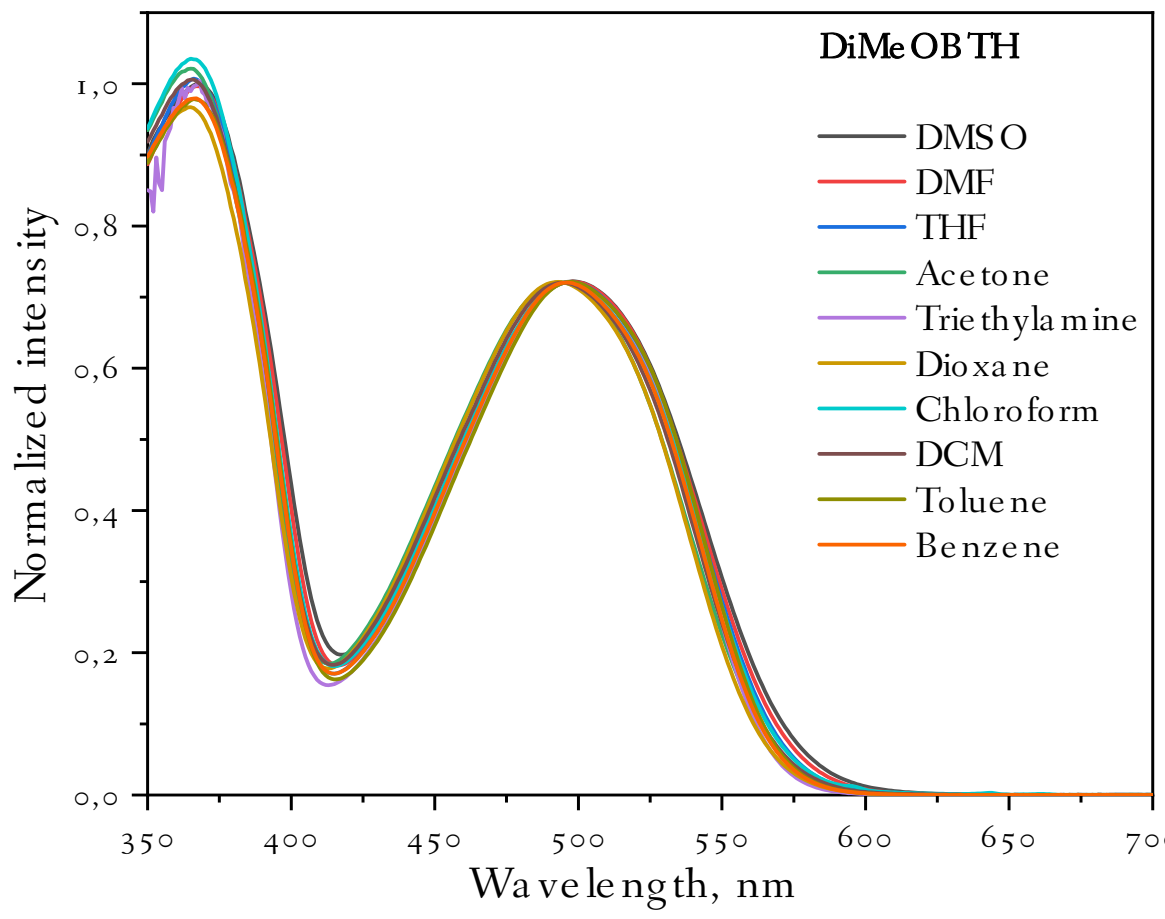

b

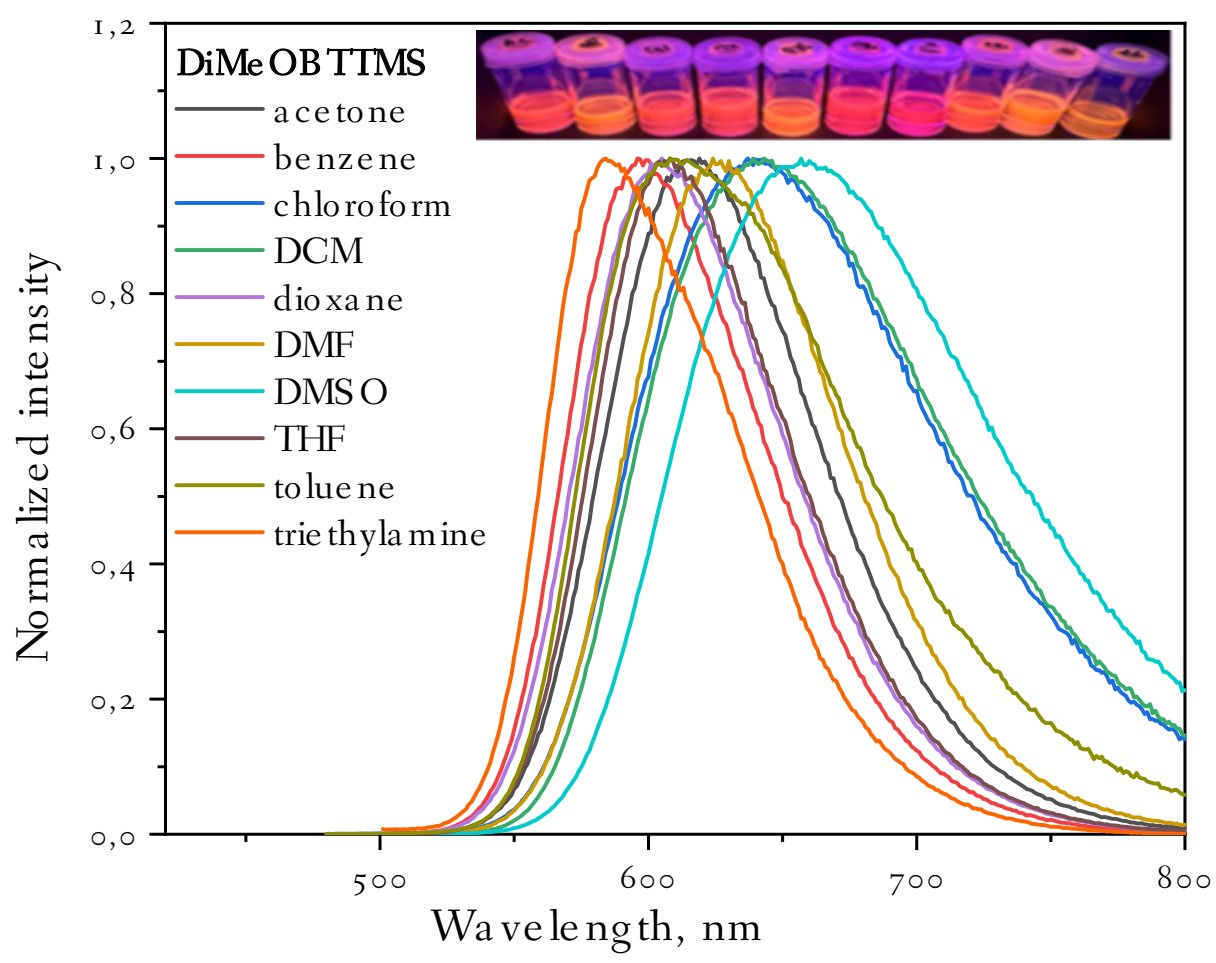

c

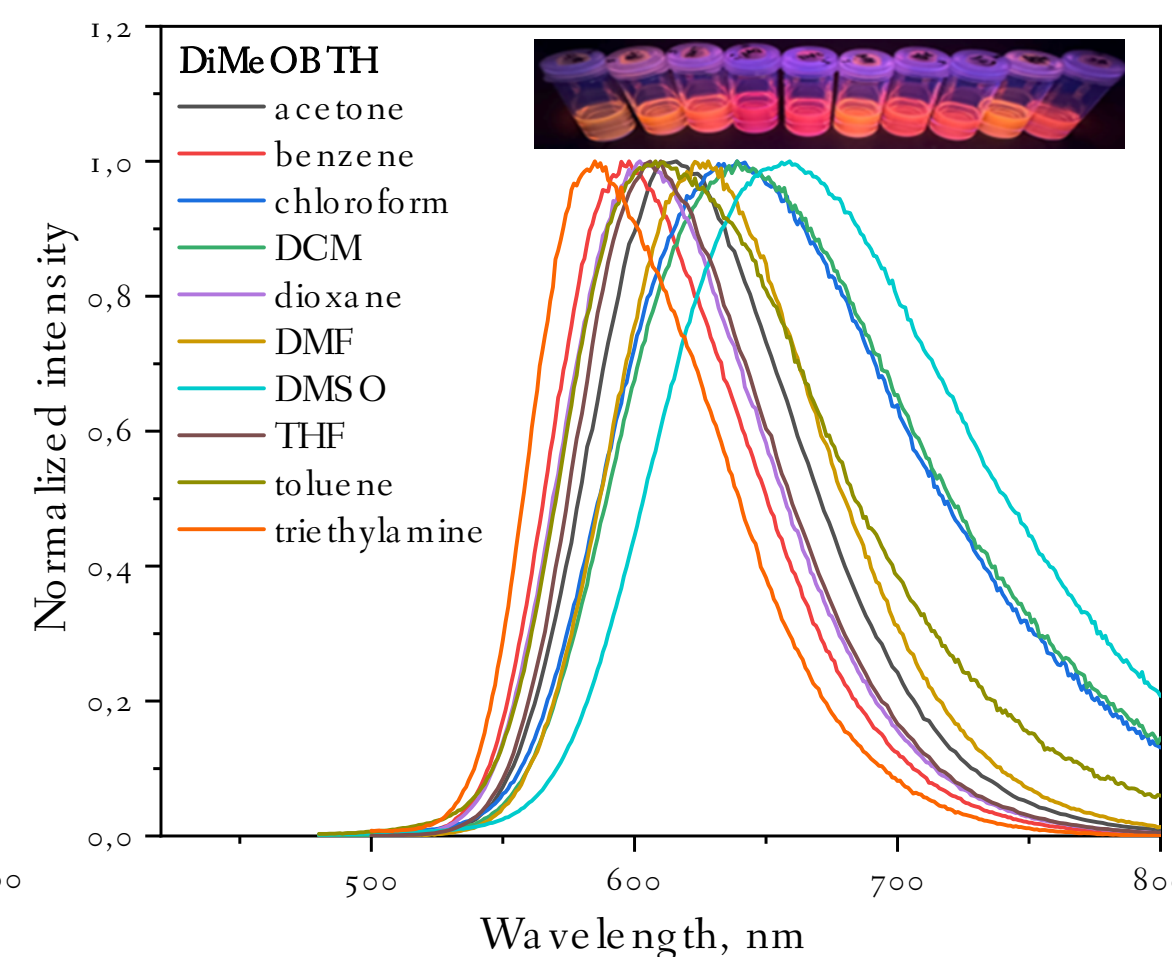

d

Figure S6. Absorption (a, b) and PL (a, b) spectra of the dilute solutions of DiMeOBTTMS (a, c) and DiMeOBTH (b, d) compounds in different solvents.

Table S1. Photophysical parameters of DiMeOBTTMS and DiMeOBTH in different solvents.

Solvent	DiMeOBTTMS				DiMeOBTH			
	λ, nm		FWHM, nm	τ1, ns	λ, nm		FWHM, nm	τ1, ns
	abs	PL			abs	PL		
Acetone	367/494	616	92	5.58	364/493	616	91	5.84
Benzene	368/495	598	85	4.41	366/496	597	85	4.60
Chloroform	367/496	637	130	5.52	365/495	639	131	5.75
CH_2Cl_2	367/496	639	131	5.64	365/494	639	131	5.90
Dioxane	367/494	603	87	4.98	365/492	604	88	5.20
DMF	370/498	625	92	5.53	367/497	626	93	5.75
DMSO	371/500	659	138	5.54	367/498	659	137	5.72
THF	369/498	607	84	5.13	366/495	606	85	5.40
Toluene	369/498	608	114	4.37	366/497	608	114	4.59
Triethylamine	366/497	584	81	4.28	365/495	583	82	4.53

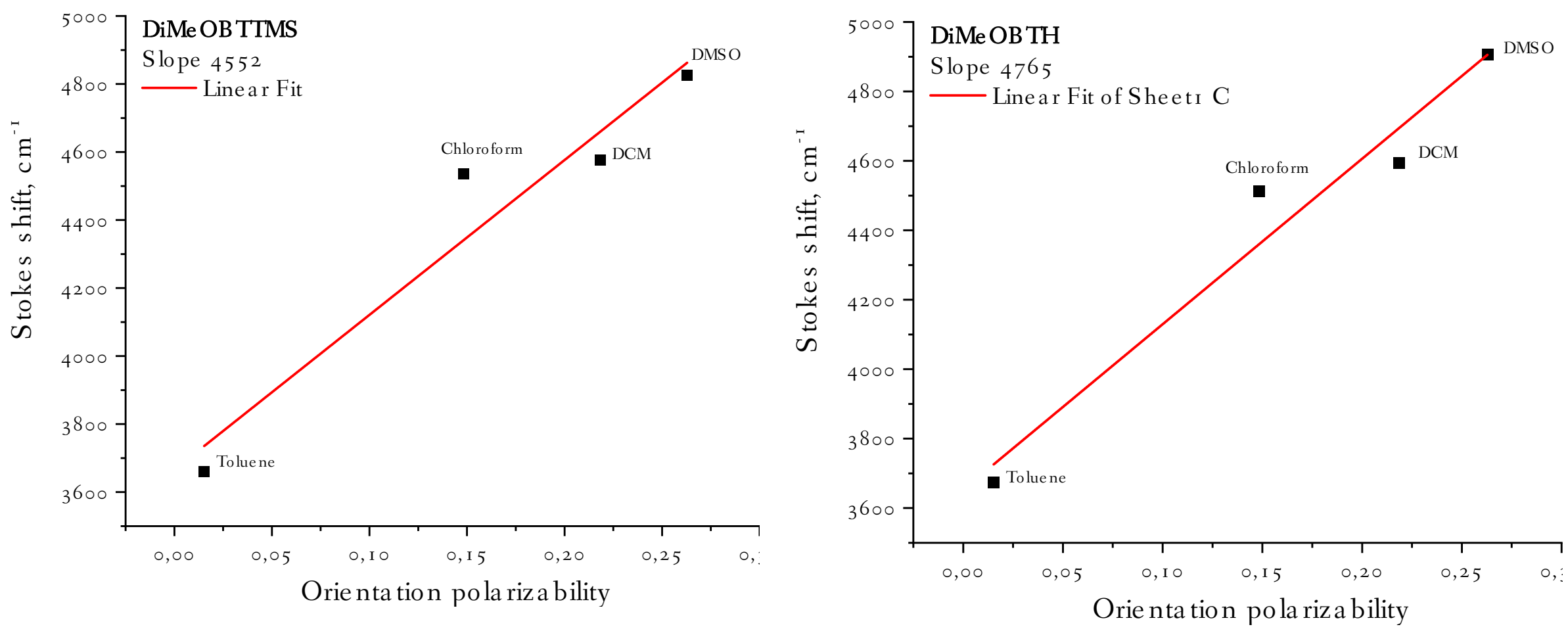

Figure S7. Stokes shift as a function of orientation polarizability (Lippert–Mataga plots) of the different solutions of the compounds DiMeOBTTMS and DiMeOBTH. The Lippert–Mataga plots were plotted according to the formula $\Delta\tilde{\nu} = \frac{2\Delta f}{4\pi\varepsilon_o hca^3}\left(\mu_e - \mu_g\right)^2 + \Delta\tilde{\nu}^o$, where $\Delta\tilde{\nu}$ is the Stokes shift assuming no solvation effect, h is Planck's constant, c is the velocity of

light in vacuum, and $\square_o$ is the permittivity of the free space, and a is the Onsager cavity radius, $\square$f is the orientational polarizability of the solvent. Calculated slopes associated with the change in the dipole moment of the compound in the ground ($\square_g$) and excited ($\square_e$) states. [11].

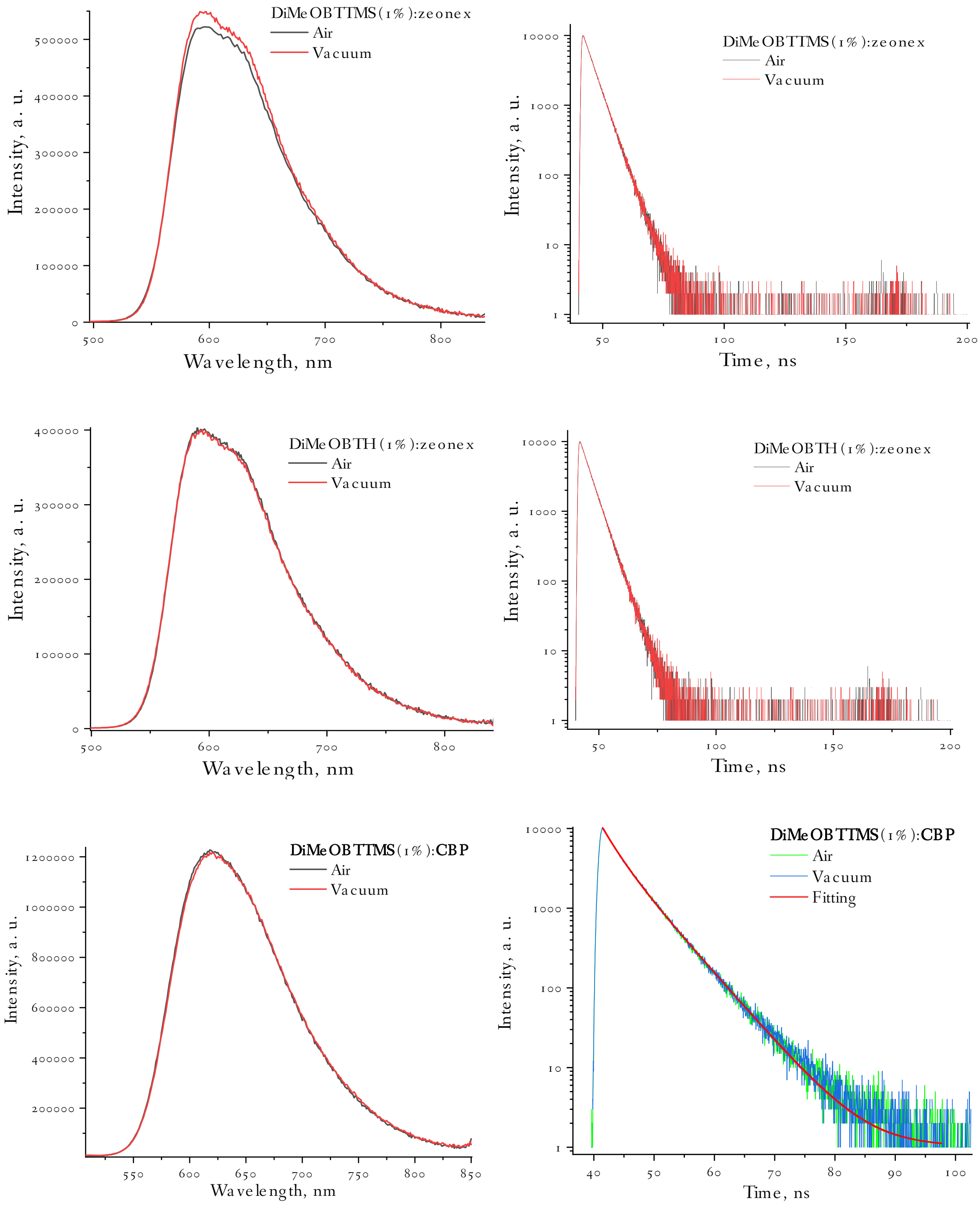

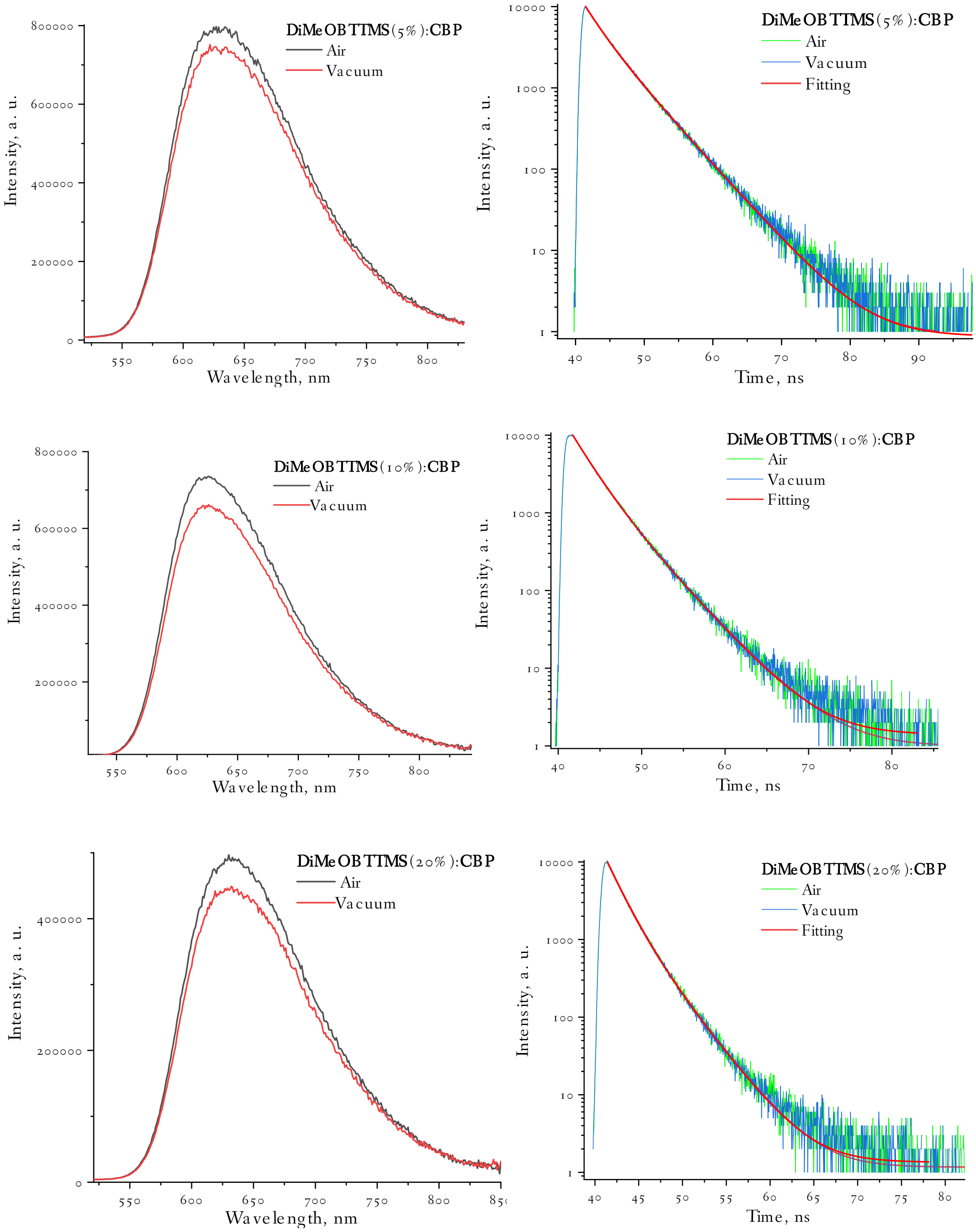

DiMeOBTTMS(5%):CBP
Air
Vacuum
Intensity, a. u.
Wavelength, nm
DiMeOBTTMS(5%):CBP
Air
Vacuum
Fitting
Intensity, a. u.
Time, ns
DiMeOBTTMS(10%):CBP
Air
Vacuum
Intensity, a. u.
Wavelength, nm
DiMeOBTTMS(10%):CBP
Air
Vacuum
Fitting
Intensity, a. u.
Time, ns
DiMeOBTTMS(20%):CBP
Air
Vacuum
Intensity, a. u.
Wavelength, nm
DiMeOBTTMS(20%):CBP
Air
Vacuum
Fitting
Intensity, a. u.
Time, ns

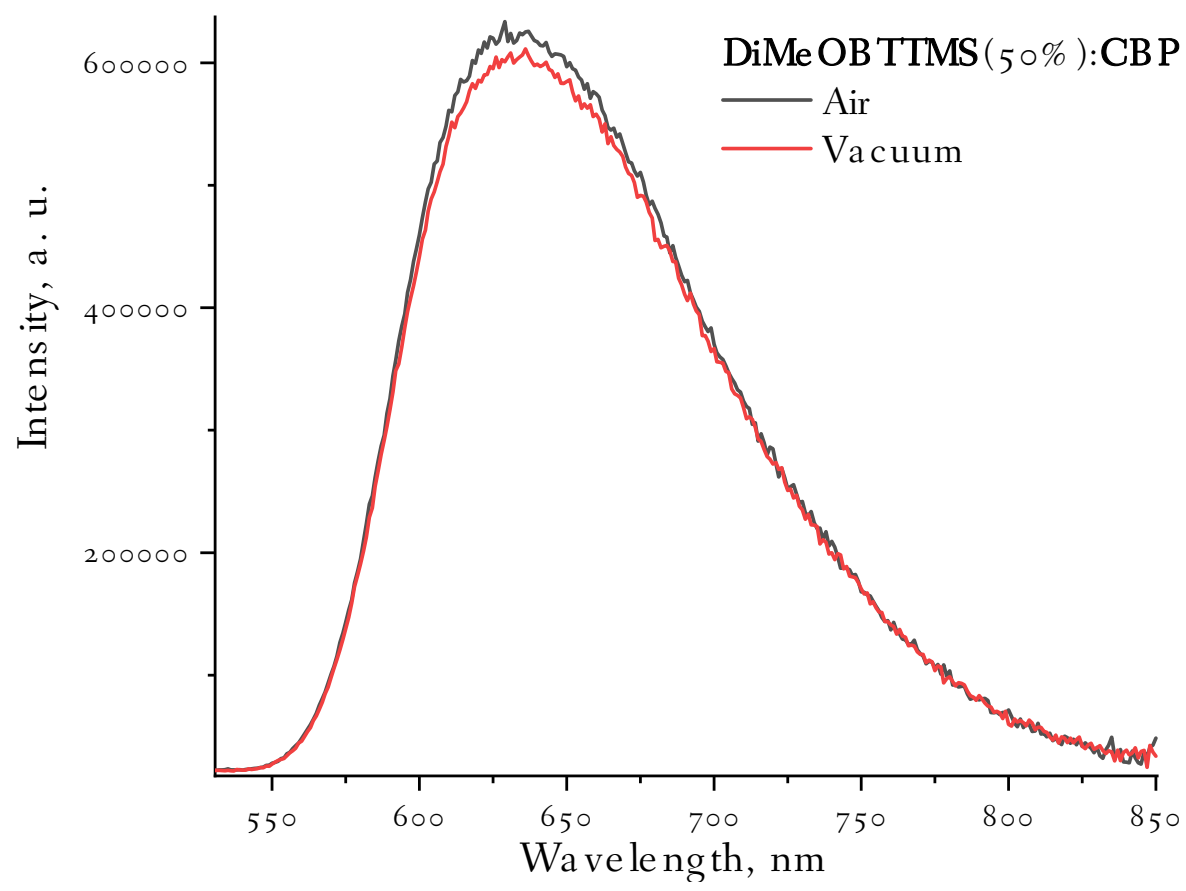
DiMeOBTTMS(50%):CBP
Air
Vacuum
Intensity, a. u.
600000
400000
200000
550
600
650
700
750
800
850
Wavelength, nm

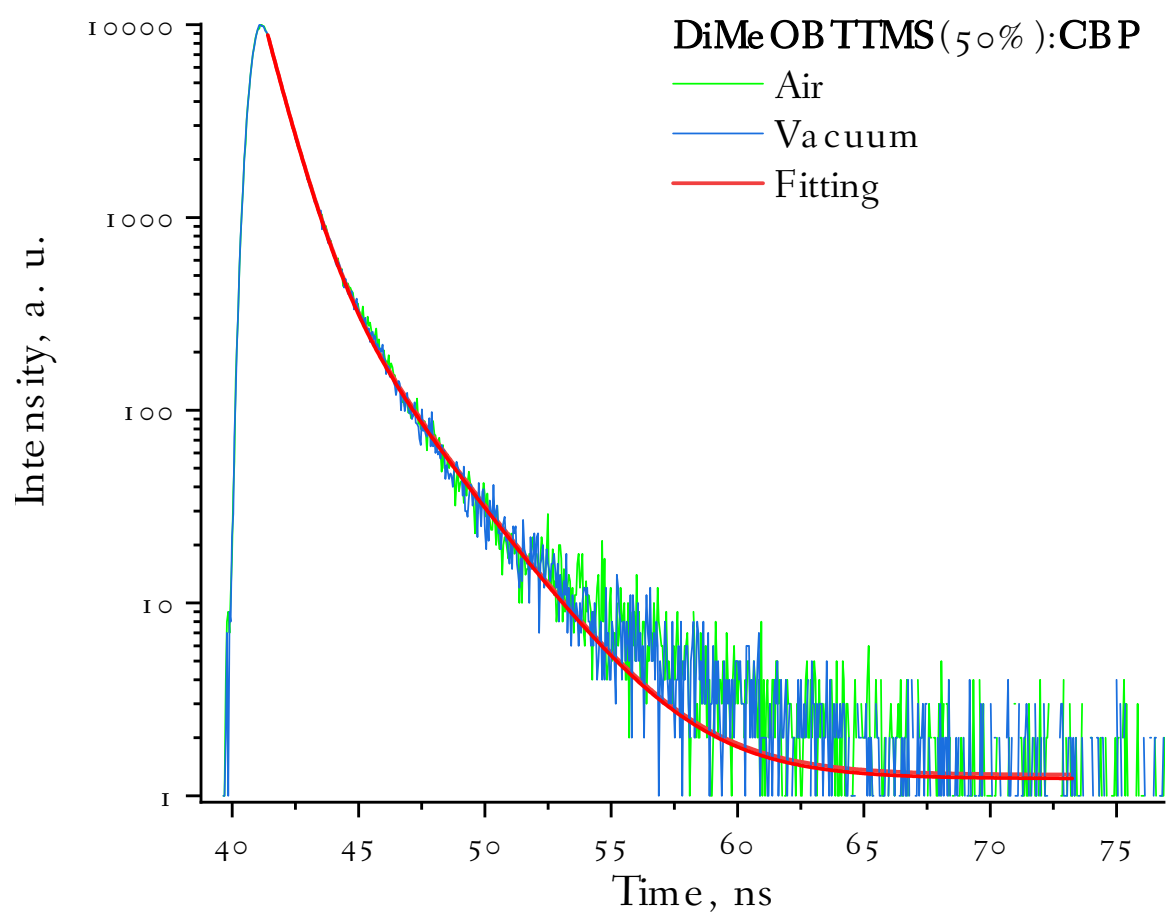
DiMeOBTTMS(50%):CBP
Air
Vacuum
Fitting
Intensity, a. u.
10000
1000
100
10
1
40
45
50
55
60
65
70
75
Time, ns

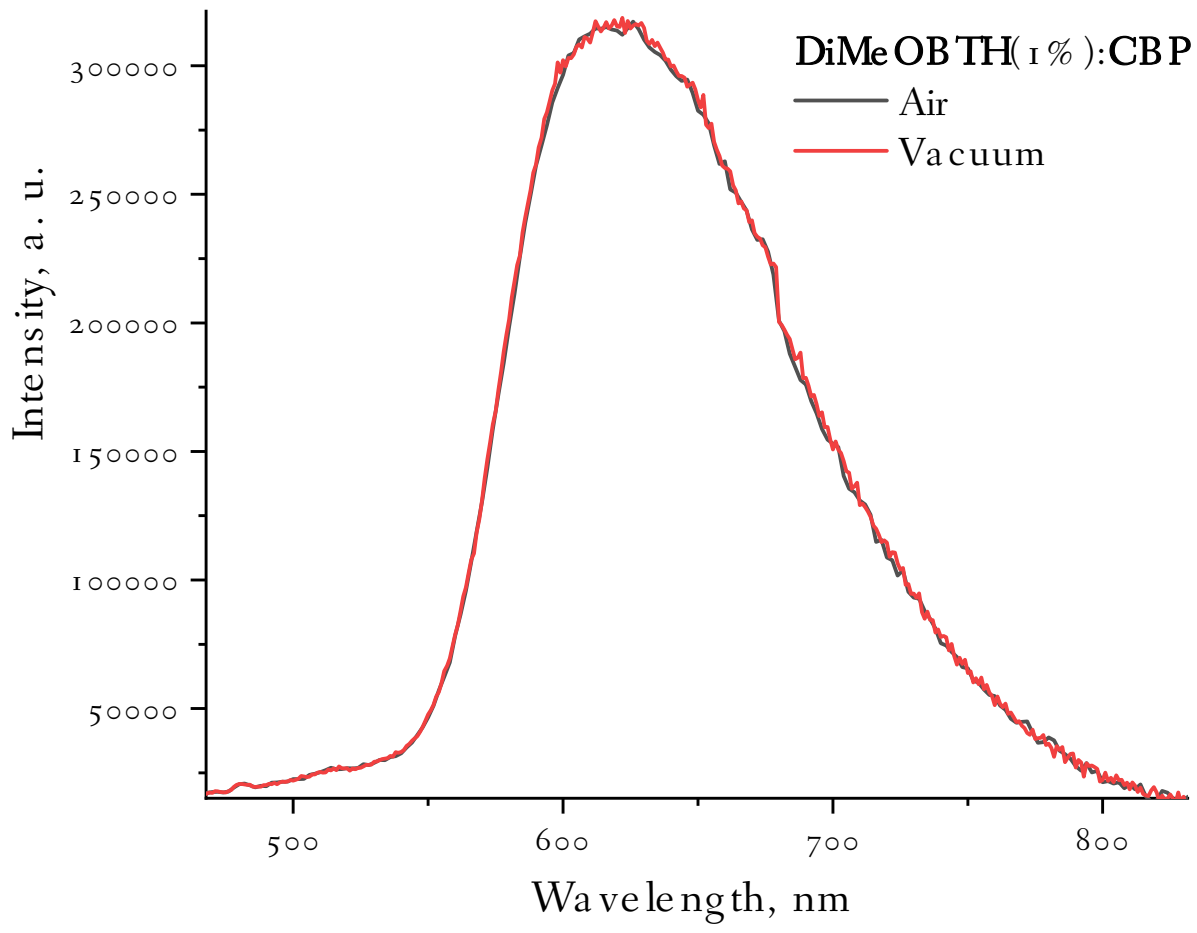
DiMeOBTH(1%):CBP
Air
Vacuum
Intensity, a. u.
300000
250000
200000
150000
100000
50000
500
600
700
800
Wavelength, nm

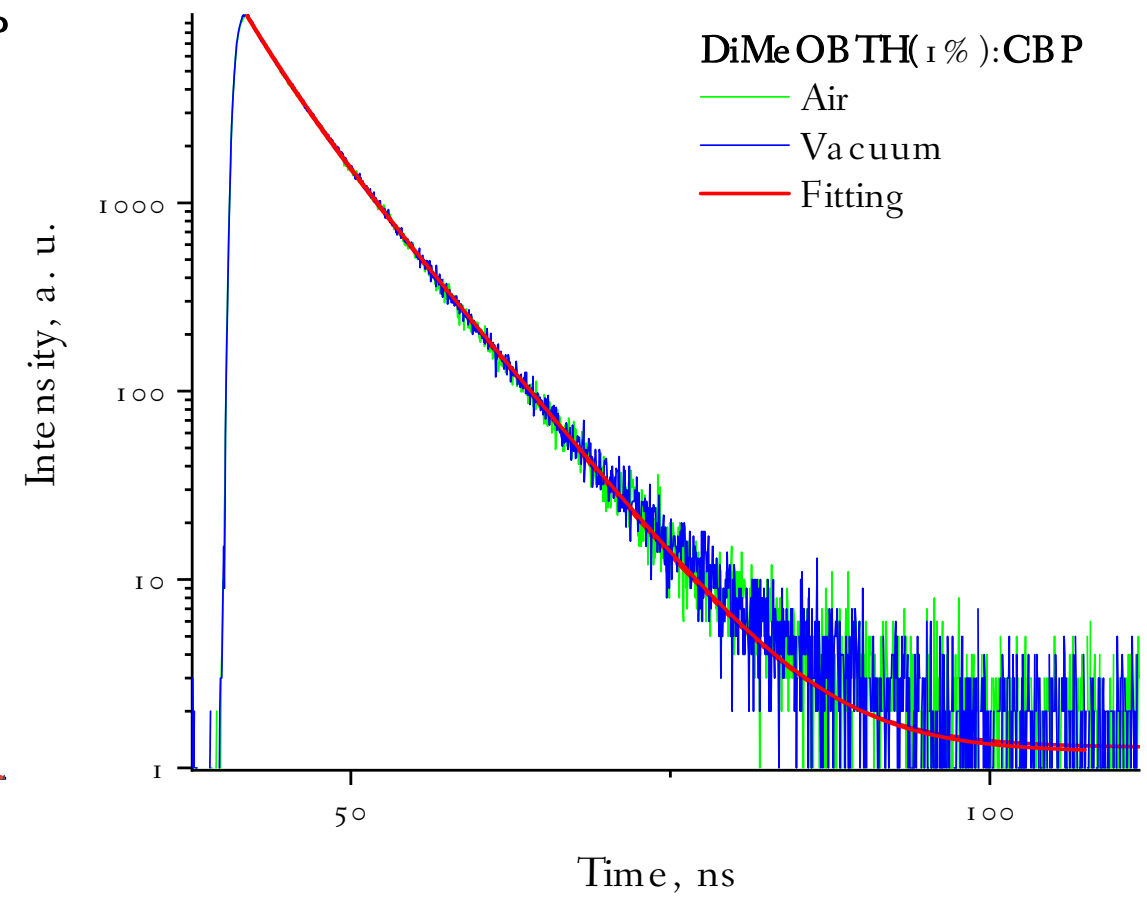
DiMeOBTH(1%):CBP
Air
Vacuum
Fitting
Intensity, a. u.
1000
100
10
1
50
100
Time, ns

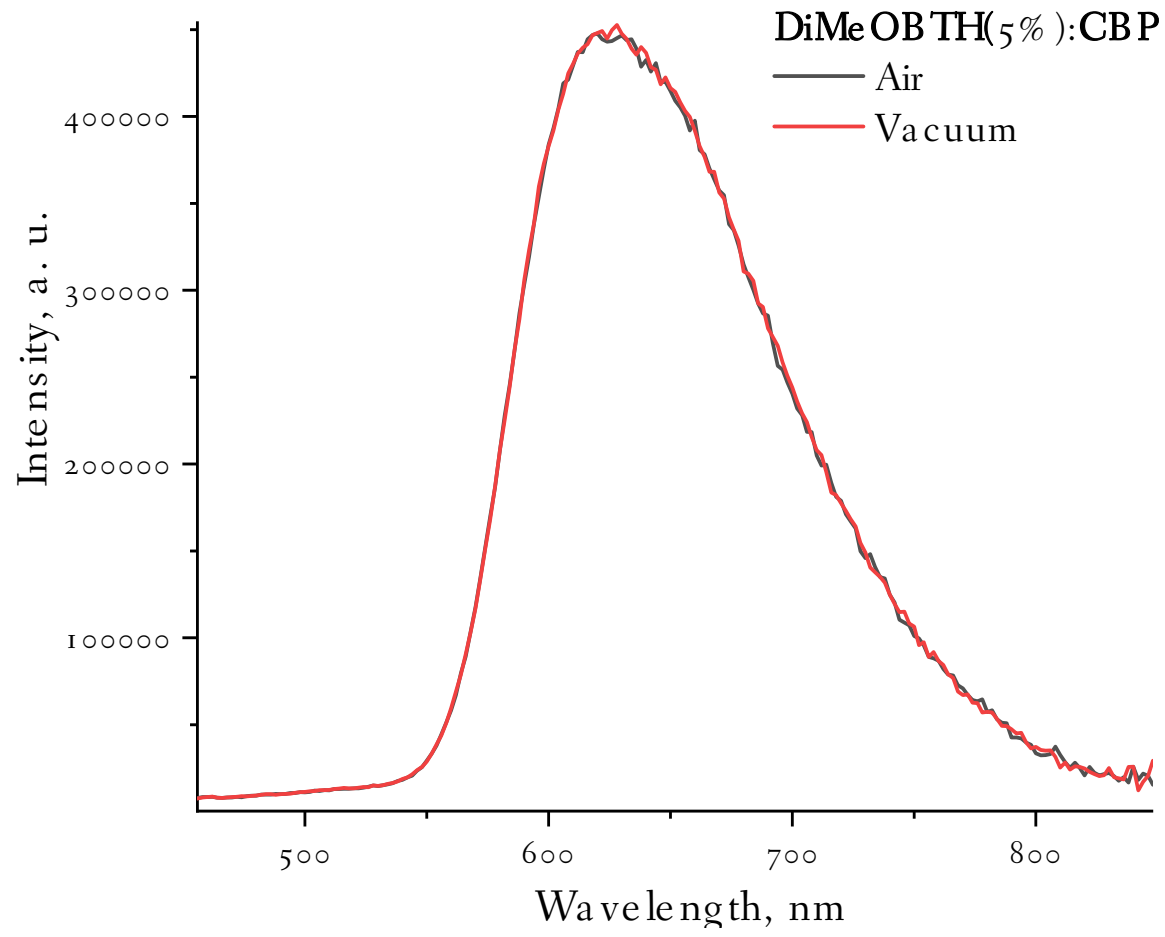
DiMeOBTH(5%):CBP
Air
Vacuum
Intensity, a. u.
400000
300000
200000
100000
500
600
700
800
Wavelength, nm

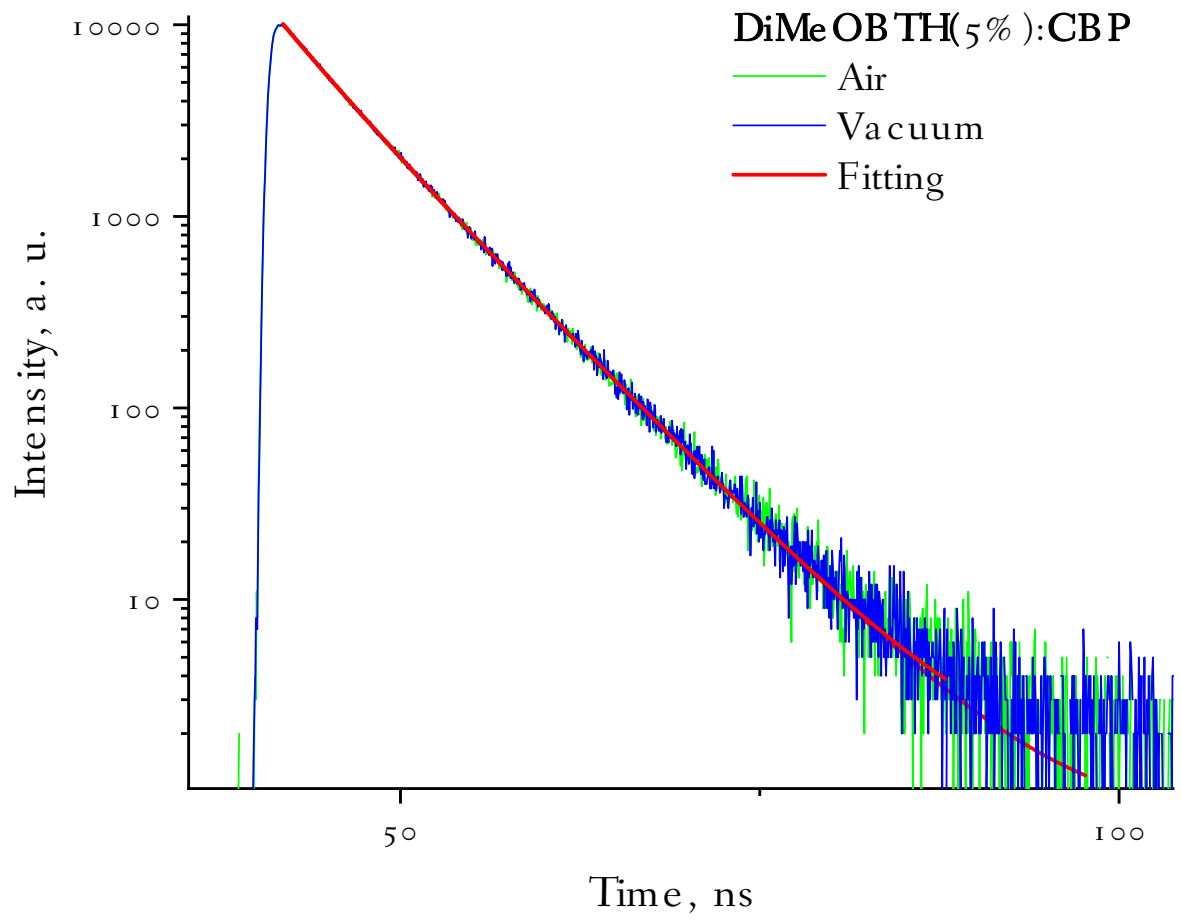
DiMeOBTH(5%):CBP
Air
Vacuum
Fitting
Intensity, a. u.
10000
1000
100
10
50
100
Time, ns

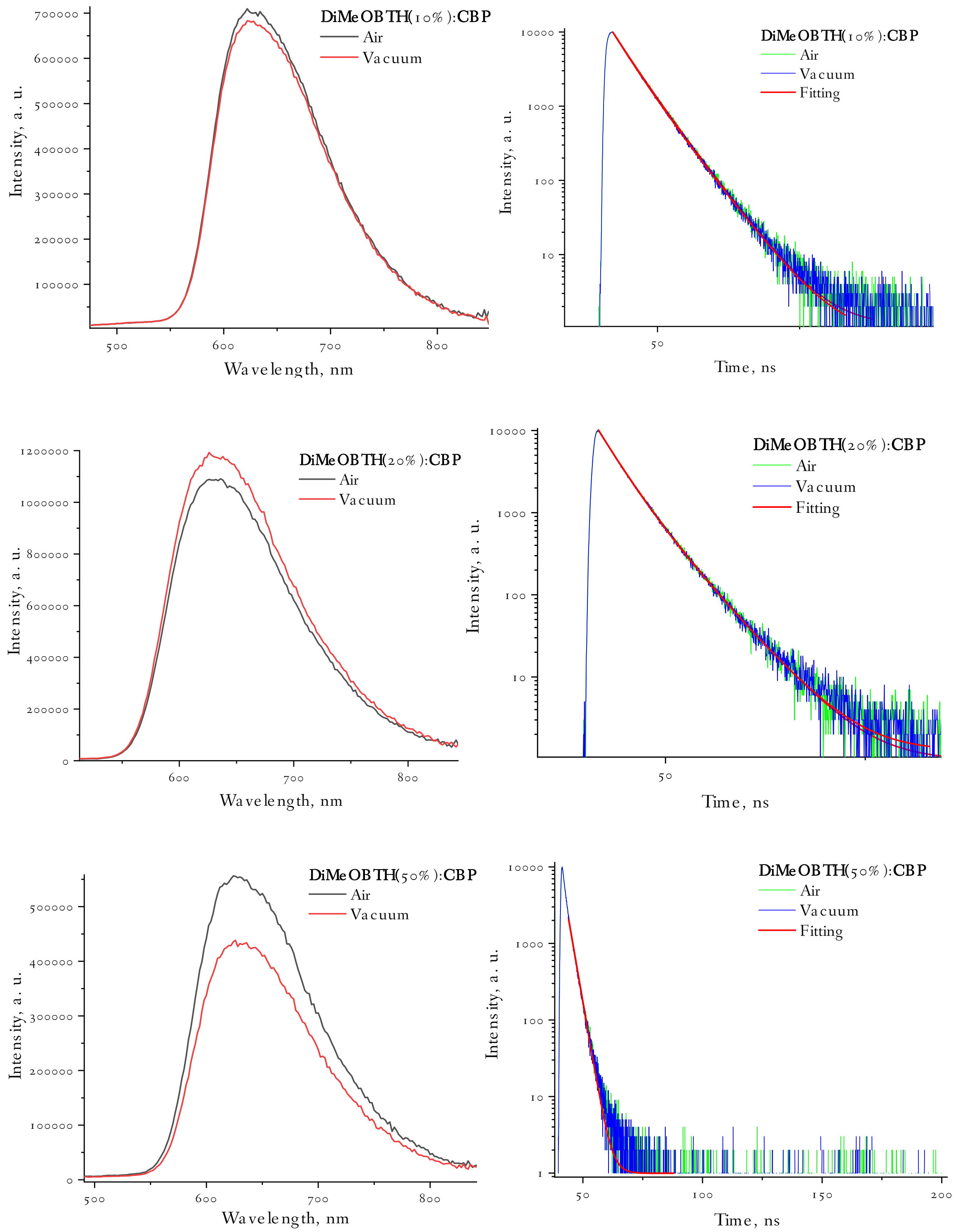

Figure S8. PL spectra (the left) and PL decay curves (the right) of Zeonex or CBP-containing films with different concentrations of DiMeOBTTMS and DiMeOBTH recorded under air or vacuum.

Table S2. Photophysical parameters of films of DiMeOBTTMS and DiMeOBTH.

Film	λ, nm	FWHM, nm	τ1, ns	τ2, ns	PLQY, %
DiMeOBTTMS	643	120	0.92	2.05	36.13
DiMeOBTH	639	122	1.17	2.07	15.08
DiMeOBTTMS : zeonex	594	108	4.16	-	65.03
DiMeOBTH : zeonex	592	105	4.23	-	70.99
DiMeOBTTMS (1%):CBP	617	117	2.20	5.03	45.69
DiMeOBTTMS (5%):CBP	629	121	2.34	4.70	31.47
DiMeOBTTMS (10%):CBP	625	113	2.20	4.05	28.6
DiMeOBTTMS (20%):CBP	631	119	1.60	3.13	15.69
DiMeOBTTMS (50%):CBP	632	120	0.82	2.52	5.67
DiMeOBTH (1%):CBP	617	120	2.60	5.32	48.13
DiMeOBTH (5%):CBP	623	123	3.74	5.96	38.89
DiMeOBTH (10%):CBP	626	120	2.97	4.53	33.34
DiMeOBTH (20%):CBP	631	122	2.38	4.20	25.89
DiMeOBTH (50%):CBP	628	120	2.39	-	4.58

Table S3. Photophysical parameters of films of DiMeOBTTMS and DiMeOBTH under air and vacuum.

% air or vac	$τ_1$, ns[a]	$τ_2$, ns[a]	A1[b]	A2[b]	$χ^{2c}$
DiMeOBTTMS:CBP					
1% air	2.20	5.03	4106.16	6031.53	1.064
1% vac	2.33	5.09	4419.78	5932.22	1.150
5% air	2.34	4.70	4249.68	5764.25	1.039
5% vac	2.32	4.70	4201.72	5974.22	1.061

10% air	2.20	4.05	7458.81	2880.18	1.059
10%vac	2.07	3.83	6410.77	3552.28	1.026
20% air	1.60	3.13	7837.23	2673.47	1.095
20% vac	1.58	3.08	7277.04	2715.88	1.015
50% air	0.82	2.52	7953.86	932.44	1.171
50% vac	0.82	2.52	8433.91	914.99	1.063
DiMeOBTH:CBP					
1% air	2.60	5.32	3642.11	6201.42	1.069
1% vac	2.70	5.37	3901.13	6187.87	1.080
5% air	3.74	5.96	3840.91	6359.29	1.115
5% vac	3.55	5.83	3125.84	7065.14	1.028
10% air	2.97	4.53	4646.55	5383.37	1.071
10%vac	3.07	4.72	6108.88	4037.05	1.007
20% air	2.38	4.20	6533.79	3229.58	1.113
20% vac	2.36	4.21	7126.94	3226.89	1.038
50% air	2.39	-	2222.18	-	1.241
50% vac	2.40	-	2071.09	-	1.267

The photoluminescence decay curves were fitted by mono or double-exponential function $I(t)=A1\exp(-t/τ_1)+A2\exp(-t/τ_2)$, $τ_1^a$ and $τ_2^a$ where emission lifetimes, $A1^b$ and $A2^b$ are the contributions, respectively, of the first and second emission lifetime components, $χ^{2c}$ is the statistical parameter of the quality of fitting experimental data to the used function.

6 Energy level and charge transport data

Table S4. Energy levels of DiMeOBTTMS and DiMeOBTH films obtained by photoelectron emission measurements.

Dye	Eg, eV[a]	I_P, eV[b]	E_A, eV[c]
**DiMeOBTTMS**	2.17	5.57	3.40
**DiMeOBTH**	2.18	5.64	3.46

[a] Calculated from sample by the equation: Eg = 1239.84/λonset.

[b] EA values were calculated from the equation: Eg = EA– Eg.

[c] Ionization energies of solid samples estimated by photoelectron emission in air.

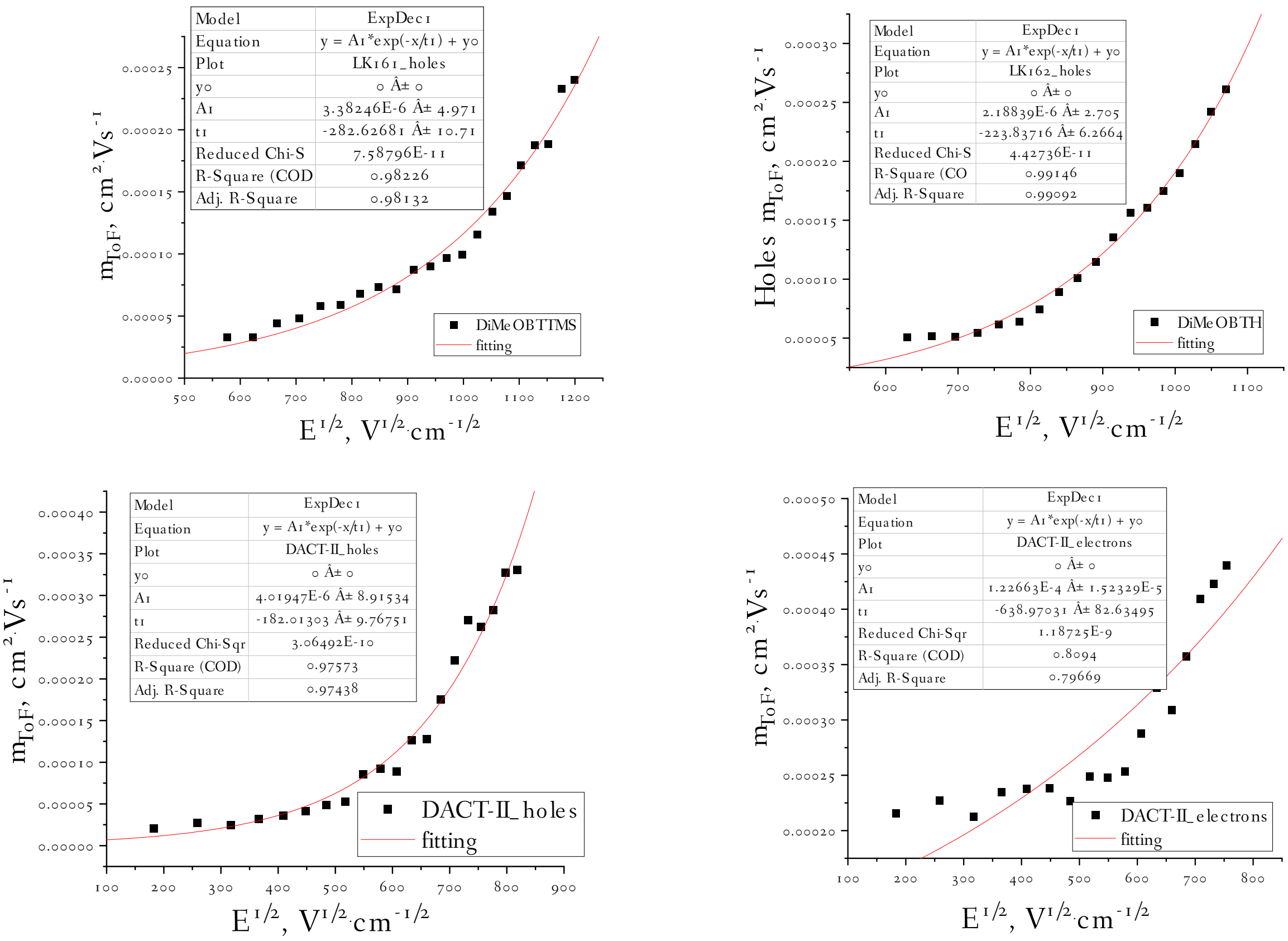

Figure S9. Fitting of electric field dependences of hole mobilities of vacuum-deposited layers of DiMeOBTTMS, DiMeOBTH and DACT-II according to the Poole-Frenkel prediction $\mu h=\mu 0\times\exp(βE1/2)$ [L. Pautmeier, R. Richert, H. Bässler, Poole-Frenkel behaviour of charge transport in organic solids with off-diagonal disorder studied by Monte Carlo simulation, Synthetic Metals 37 (1990) [12].

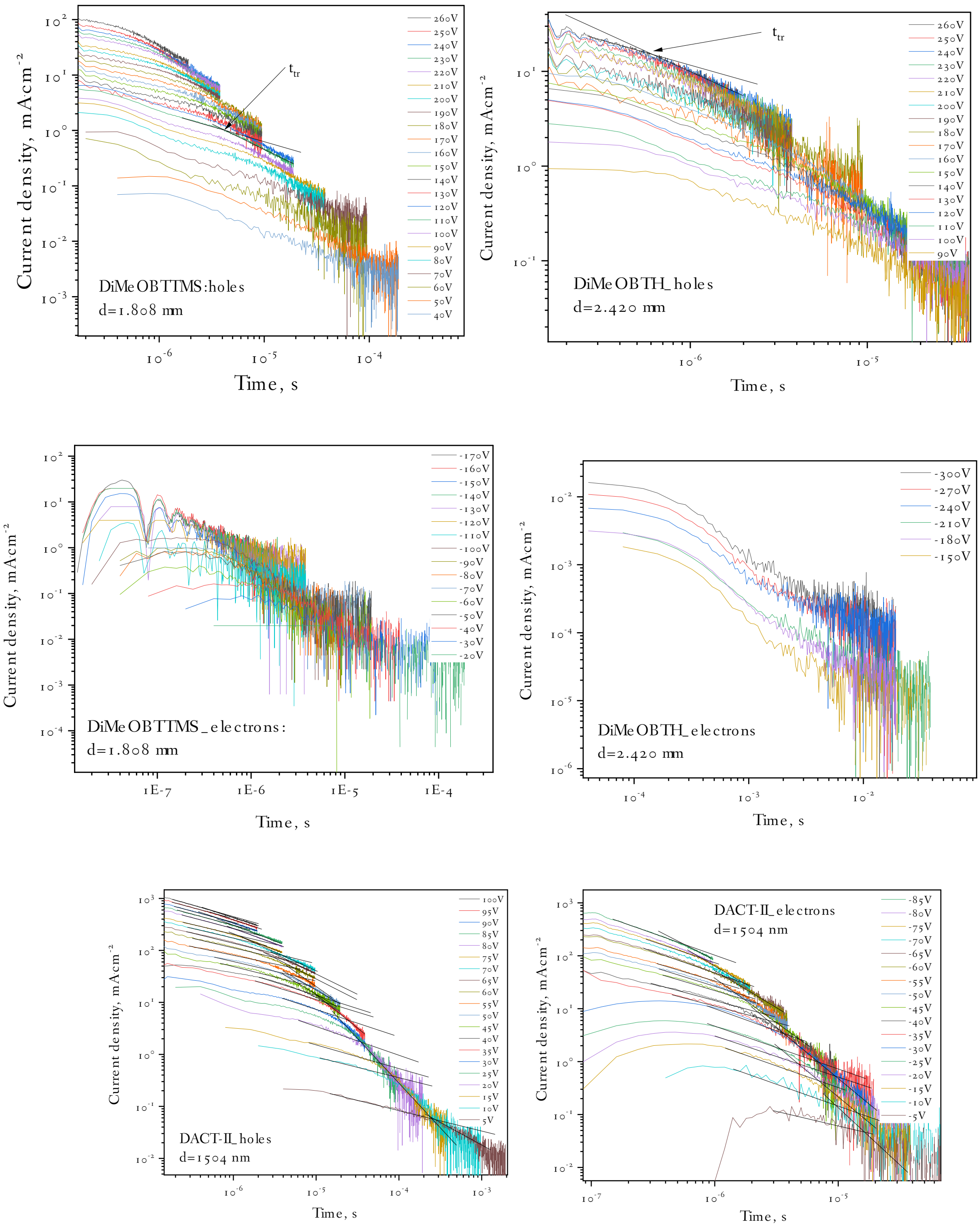

Figure S10. TOF signals for holes and electrons in vacuum-deposited films of DiMeOBTTMS, DiMeOBTH and DACT-II.

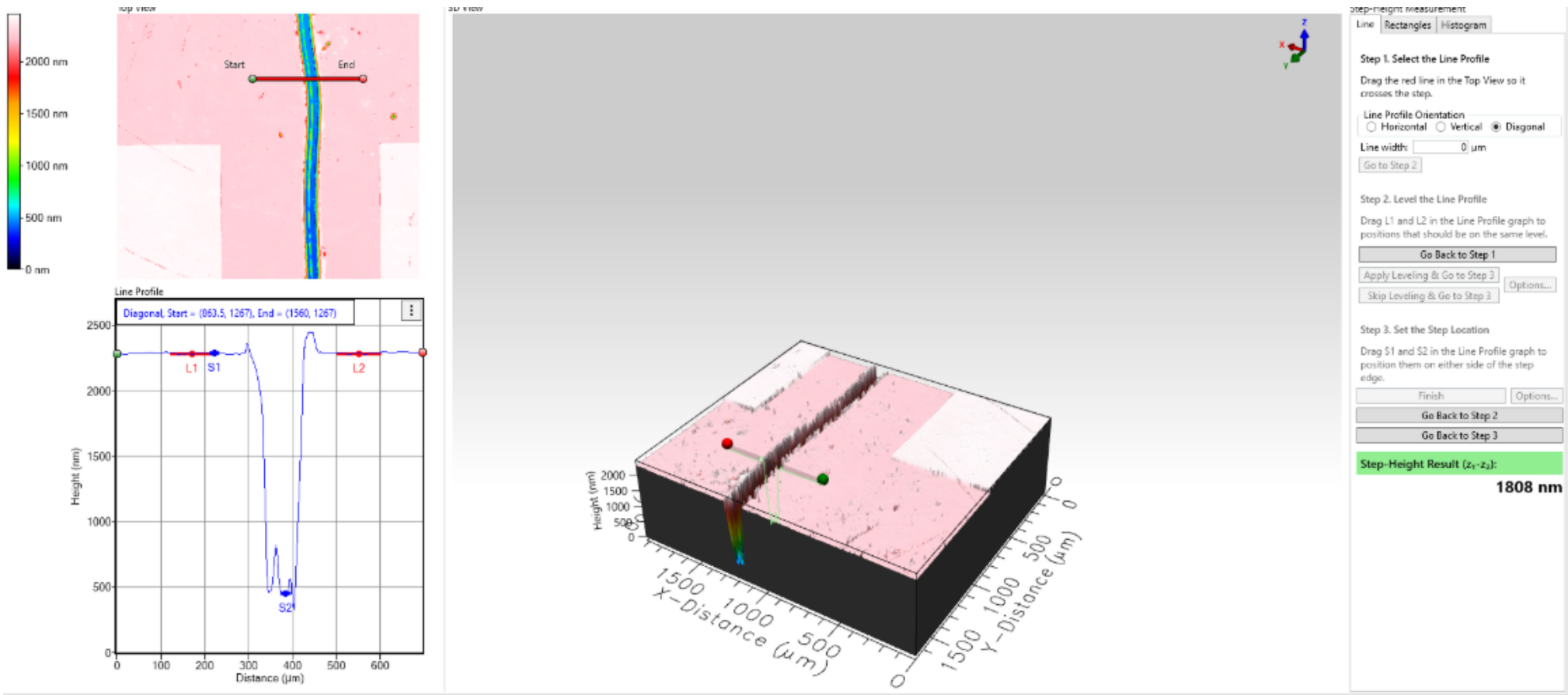

Line Profile
Step 1. Select the Line Profile
Step 2. Level the Line Profile
Step 3. Set the Step Location
Step-Height Result (z1-z2):
1808 nm
Distance (µm)
Height (nm)
X-Distance (µm)
Y-Distance (µm)

a)

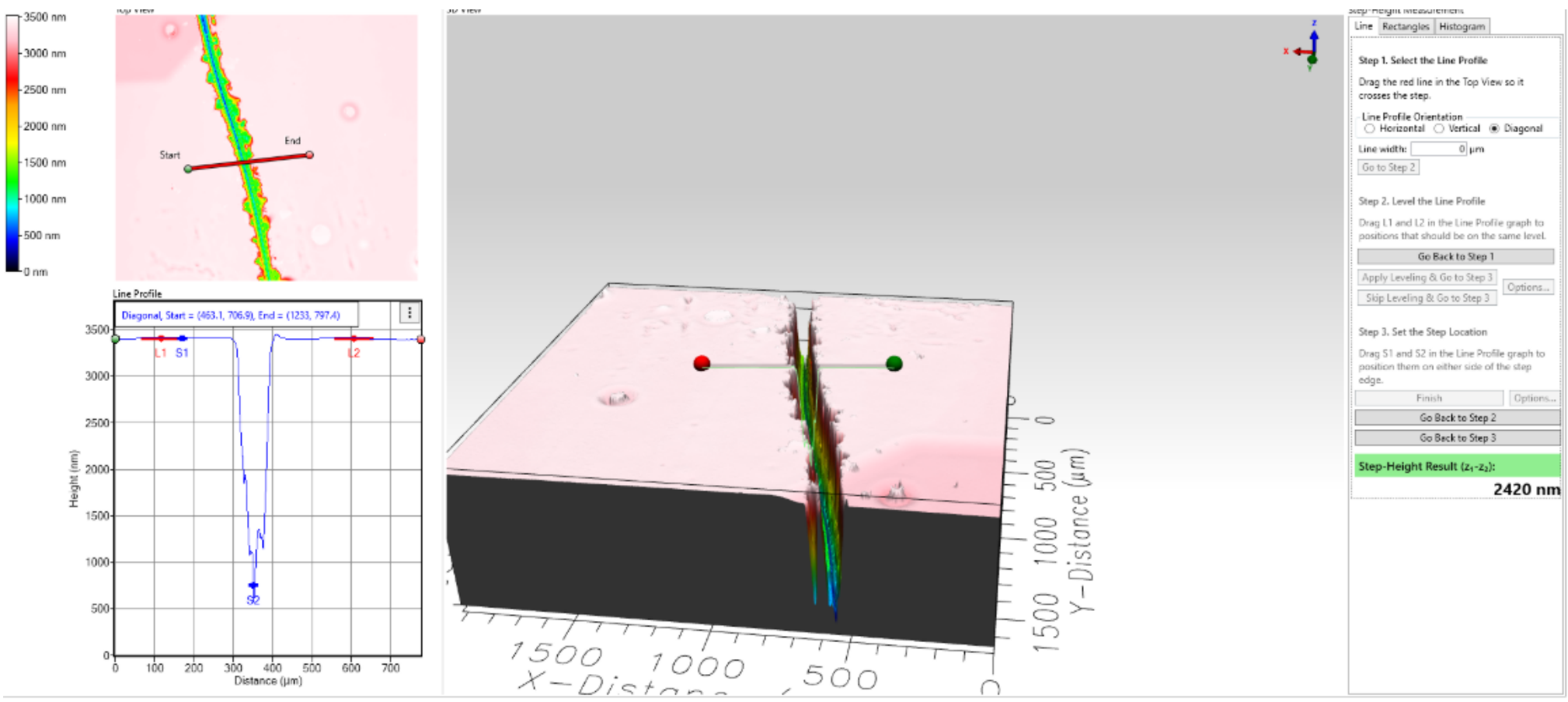

Line Profile
Step 1. Select the Line Profile
Step 2. Level the Line Profile
Step 3. Set the Step Location
Step-Height Result (z1-z2):
2420 nm
Distance (µm)
Height (nm)
X-Distance (µm)
Y-Distance (µm)

b)

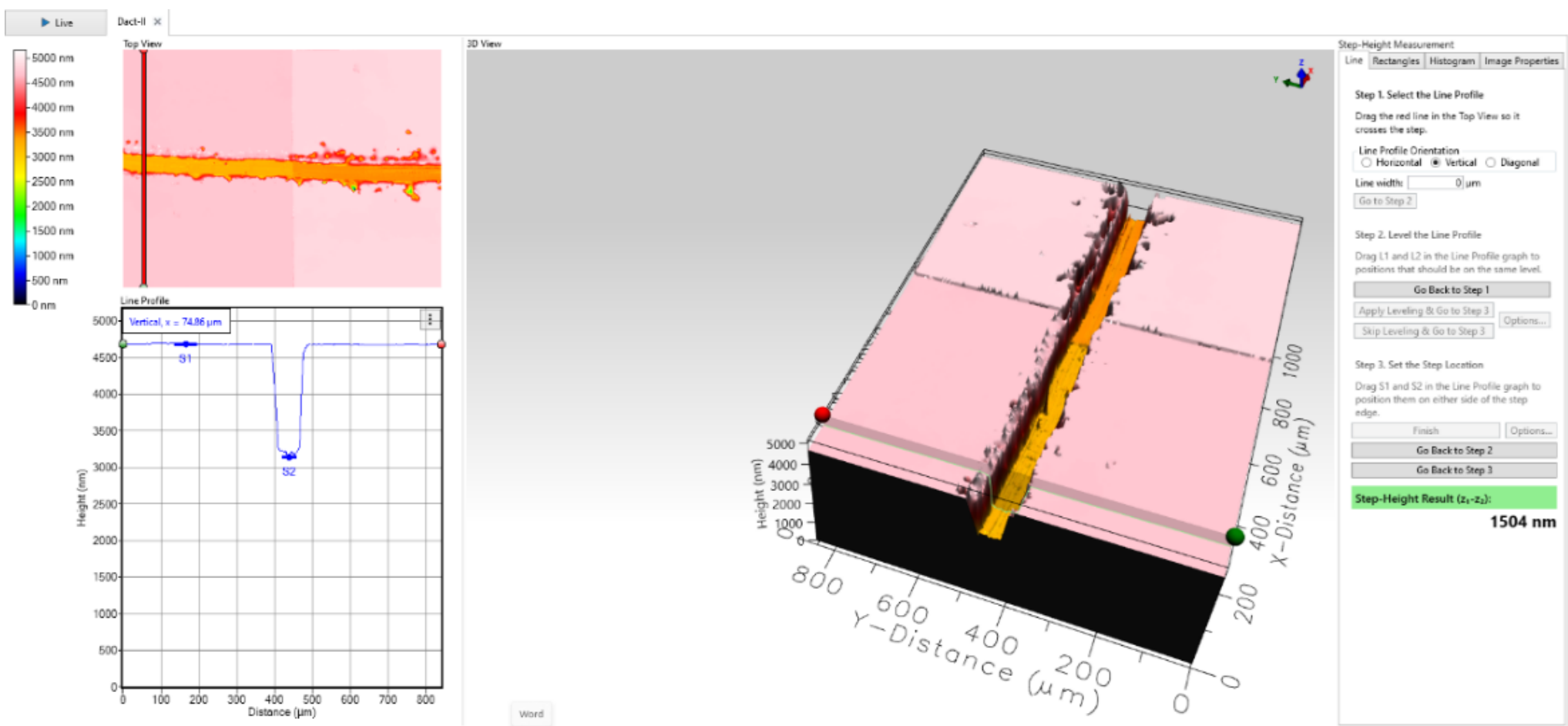

Top View
Line Profile
3D View
Step-Height Measurement
Step 1. Select the Line Profile
Step 2. Level the Line Profile
Step 3. Set the Step Location
Step-Height Result (z1-z2):
1504 nm
Distance (µm)
Height (nm)
X-Distance (µm)
Y-Distance (µm)

c)

Figure S11. Thickness measurements of vacuum-deposited films of DiMeOBTTMS (a) DiMeOBTH (b) and DACT-II (c) using the profilometer Profilm 3D.

7 OLEDs

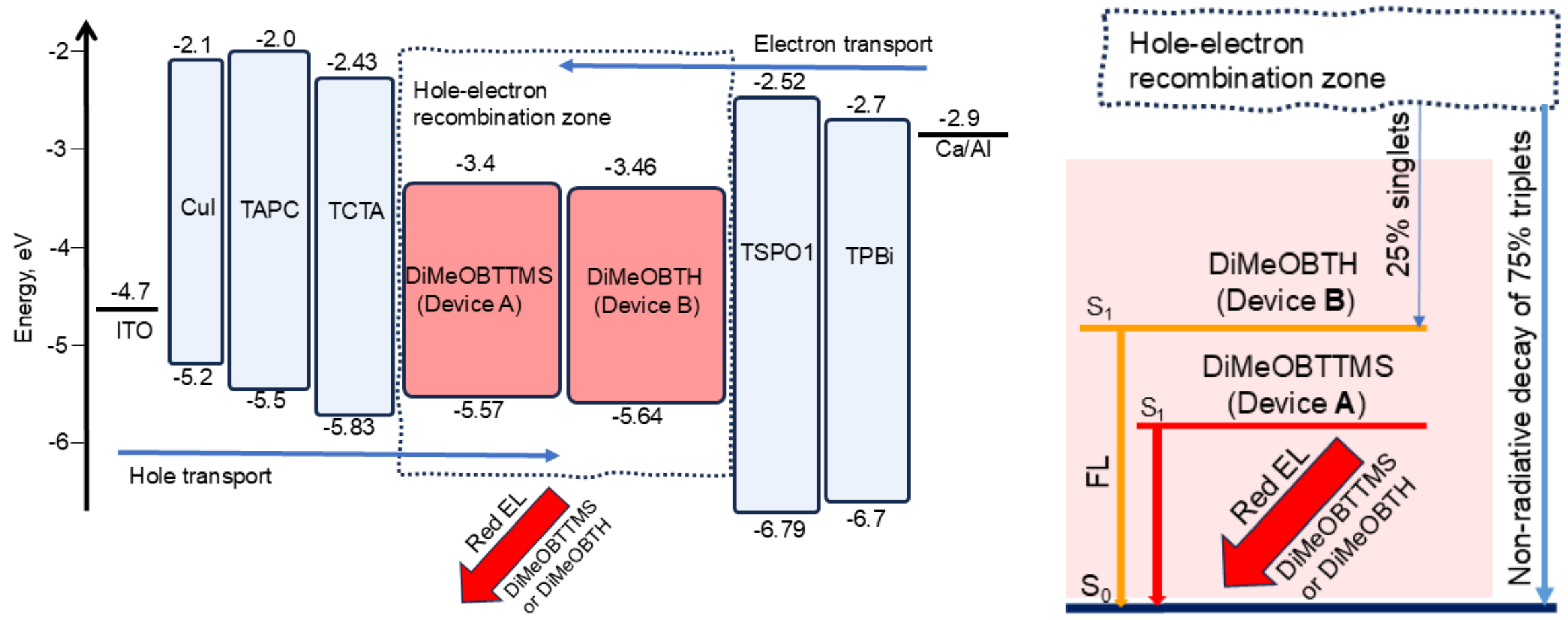

a) b)

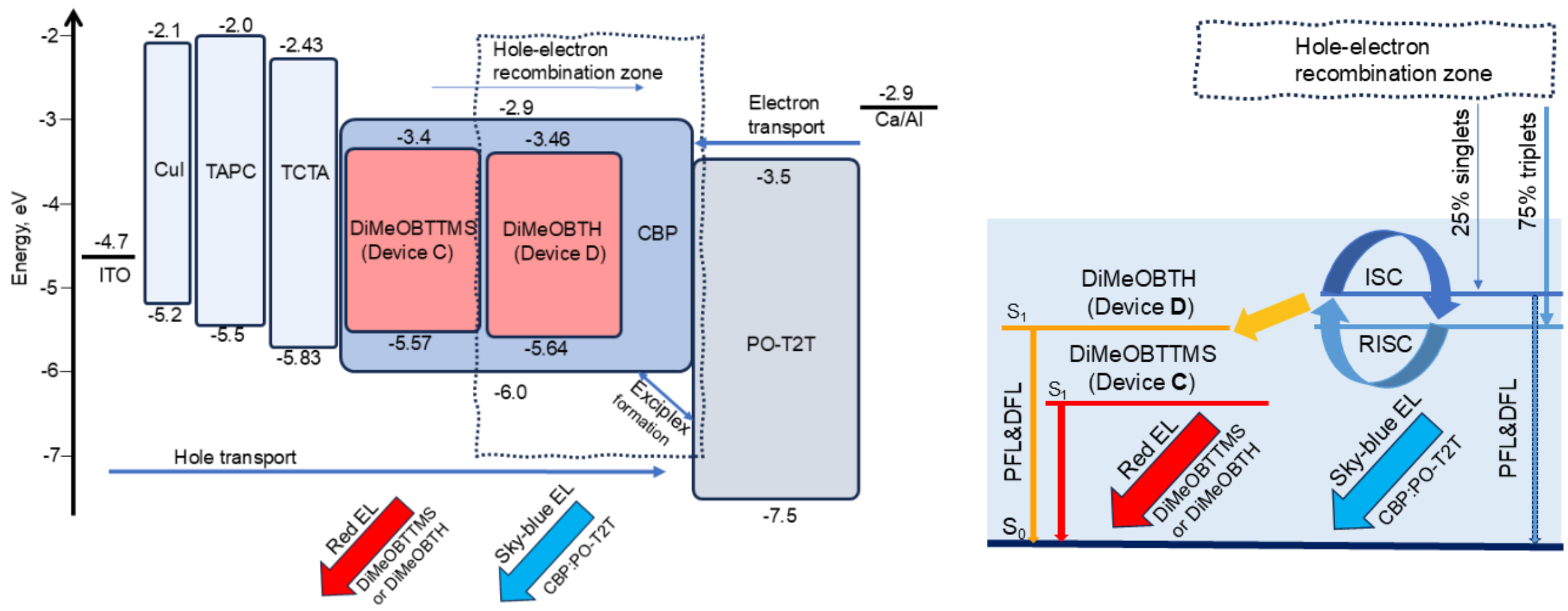

c) d)

Figure S12. Equilibrium energy diagrams (a, c) of devices A-D and schematic illustrations of Jablonski energy diagrams of corresponding light-emitting systems (b, d).

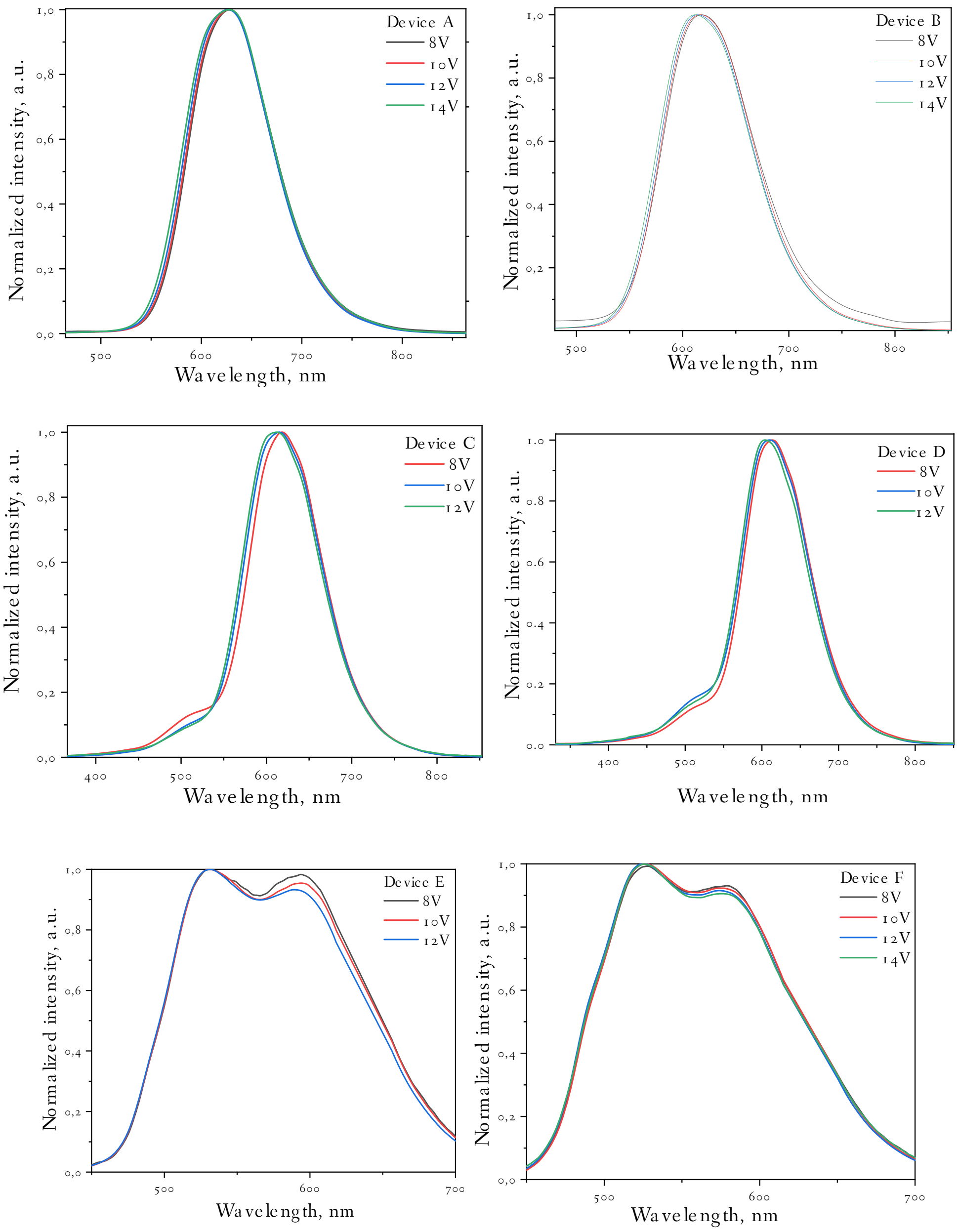

Figure S13. EL spectra of device A-F at different voltages.

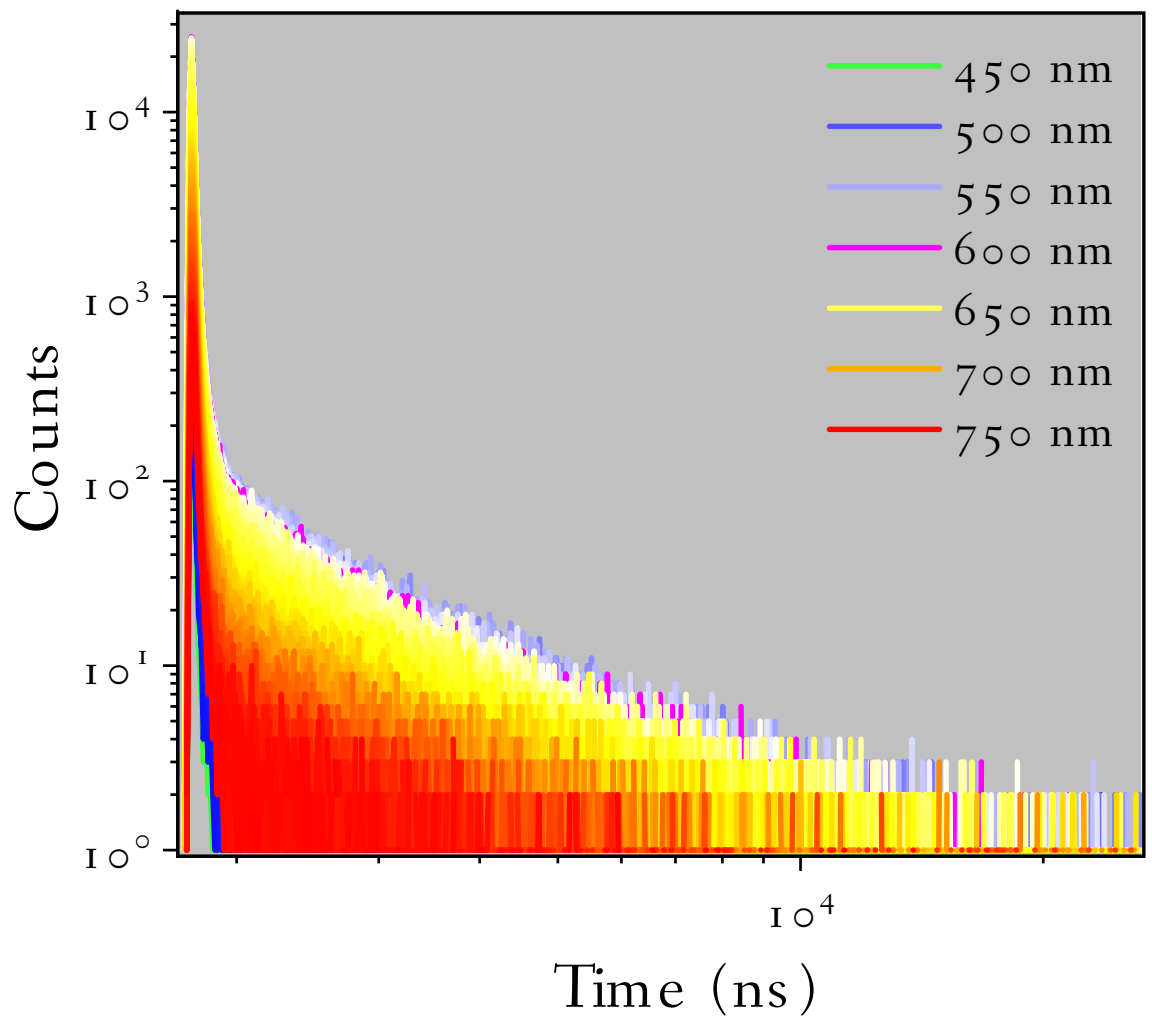

Figure S14. 256 PL decay curves recorded for DiMeOBTTMS/DACT-II-based EML during a constant time of 180 s at wavelengths from 450 to 750 nm with a step of 1 nm.

1. Coulson, D.R., L.C. Satek, and S.O. Grim, *Tetrakis(triphenylphosphine)palladium(0)*, in *Inorganic Syntheses*. 1972, McGraw-Hill, Inc. p. 121–124.
2. Gottlieb, H.E., V. Kotlyar, and A. Nudelman, *NMR Chemical Shifts of Common Laboratory Solvents as Trace Impurities.* The Journal of Organic Chemistry, 1997. **62**(21): p. 7512–7515.
3. Heeney, M., et al., *Regioregular poly(3-hexyl)selenophene: a low band gap organic hole transporting polymer.* Chemical Communications, 2007(47): p. 5061–5063.
4. Castro, E., S. Mirzaei, and R. Hernández Sánchez, *Radially Oriented [n]Cyclo-meta-phenylenes.* Organic Letters, 2021. **23**(1): p. 87–92.
5. Fan, K.W., et al., *Expanding the aqueous-based redox-facilitated self-polymerization chemistry of catecholamines to 5,6-dihydroxy-1H-benzimidazole and its 2-substituted derivatives.* RSC Advances, 2016. **6**(30): p. 25203–25214.
6. Ishi-i, T., et al., *Room temperature phosphorescence in longer-wavelength red light region found in benzothiadiazole-based dyes.* Journal of Materials Chemistry C, 2023. **11**(8): p. 3003–3009.
7. Uddin, M.A., et al., *Giant Thermosalient Effect in a Molecular Single Crystal: Dynamic Transformations and Mechanistic Insights.* Journal of the American Chemical Society, 2024. **146**(40): p. 27690–27700.
8. Belton, C.R., et al., *Location, Location, Location - Strategic Positioning of 2,1,3-Benzothiadiazole Units within Trigonal Quaterfluorene-Truxene Star-Shaped Structures.* Advanced Functional Materials, 2013. **23**(22): p. 2792–2804.
9. Frisch, M.J., et al., *Gaussian 16 Rev. C.01*. 2016: Wallingford, CT.
10. Peach, M.J.G., et al., *Excitation energies in density functional theory: An evaluation and a diagnostic test.* The Journal of Chemical Physics, 2008. **128**(4).

11. Mataga, N., Y. Kaifu, and M. Koizumi, *Solvent Effects upon Fluorescence Spectra and the Dipolemoments of Excited Molecules.* Bulletin of the Chemical Society of Japan, 2006. **29**(4): p. 465–470.
12. Pautmeier, L., R. Richert, and H. Bässler, *Poole-Frenkel behavior of charge transport in organic solids with off-diagonal disorder studied by Monte Carlo simulation.* Synthetic Metals, 1990. **37**(1): p. 271–281.
